# Identification of MBT3T as a new effective therapeutic option in imatinib-resistant gastrointestinal stromal tumors (GISTs)

**DOI:** 10.1186/s13046-026-03698-3

**Published:** 2026-03-21

**Authors:** Gloria Ravegnini, Daniele Esposito, Francesca Gorini, Tainah Dorina Marforio, Emma Coschina, Eva Benuzzi, Antonella Simone, Ahmed Samaha, Aldo Di Vito, Martina Rossi, Patrizia Hrelia, Alessandra Locatelli, Matteo Calvaresi, Nicola Facchinello, Rita Morigi, Sabrina Angelini

**Affiliations:** 1 Department of Pharmacy and Biotechnology, University of Bologna, Bologna, 40126 Italy; 2 Clinical Pharmacology Unit, IRCCS Azienda Ospedaliero-Universitaria di Bologna, Bologna, 40138 Italy; 3 Department of Chemistry “Giacomo Ciamician”, Alma Mater Studiorum - University of Bologna, Bologna, 40126 Italy; 4 Neuroscience Institute, Italian Research Council (CNR), Padua, 35131 Italy; 5 IRCCS Azienda Ospedaliero – Universitaria di Bologna, Preclinical & Translational Research in Oncology lab (PRO), Bologna, 40138 Italy

**Keywords:** GIST, TKI resistance, Tubulin

## Abstract

**Background:**

Gastrointestinal stromal tumors (GISTs) are primarily treated with tyrosine kinase inhibitors (TKIs) such as imatinib. However, the development of drug resistance remains a critical clinical challenge, highlighting the urgent need for novel therapeutic agents with alternative mechanisms of action. This study aimed to identify and characterize new small molecules effective against imatinib-sensitive and resistant GISTs.

**Methods:**

A library of 27 benzo[d]imidazo[2,1-b]thiazole derivatives was synthesized and screened for cytotoxicity against four GIST cell lines, including imatinib-sensitive (GIST-T1, GIST-882) and imatinib-resistant (GIST-48, GIST-48B) models. The most effective compound, MBT3T, was further evaluated for safety on healthy cells (PBMCs, fibroblasts) and mechanism of action using computational docking, immunofluorescence, and flow cytometry. Efficacy was validated both in 3D bioprinted tumor models and in vivo using zebrafish xenografts.

**Results:**

MBT3T exhibited potent nanomolar cytotoxicity across all GIST cell lines while maintaining a favorable safety profile on healthy cells. Mechanistic studies revealed that MBT3T acts independently of the KIT signaling pathway, targeting tubulin at the colchicine-binding site. This interaction caused microtubule disassembly, leading to G2/M cell cycle arrest and caspase-mediated apoptosis. In in vivo zebrafish assays, MBT3T significantly reduced tumor growth in both imatinib-sensitive and resistant xenografts without inducing systemic toxicity.

**Conclusion:**

We identified MBT3T as a novel, selective tubulin polymerization inhibitor. Its ability to bypass TKIs resistance mechanisms suggests it may represent a promising therapeutic candidate for the treatment of refractory GISTs.

**Supplementary Information:**

The online version contains supplementary material available at 10.1186/s13046-026-03698-3.

## Background

Gastrointestinal stromal tumors (GISTs) are rare mesenchymal cancers affecting the gastrointestinal tract, characterized by gain-of-function mutations in tyrosine kinase (TK) receptors KIT or PDGFRA in about 85–90% of the cases [1, 2]. The remaining 10–15% of cases, wild type for KIT/PDGFRA genes are a very heterogeneous group of tumors, showing high variability in driving cancer events [3, 4]. GIST treatment represents the best examples of target therapy in solid malignancies, with patients experiencing an extraordinary improvement in clinical outcome in the early 2000s, after the introduction of TK inhibitors (TKIs). In this context, the standard-of-care for unresectable or metastatic GISTs is the TKI imatinib, a small molecule leading to relevant clinical benefits, becoming the first breakthrough medical treatment for advanced-stage GISTs [5]. Despite the great achievements in targeted therapy for GIST, imatinib resistance occurs frequently and, in most patients, within 18–24 months [6]. Clinical and biomedical investigations showed the occurrence of imatinib-resistant clones and sparked the development of all currently FDA-approved TKIs. Unfortunately, all available therapeutic strategies showed only modest efficacy, highlighting the need for novel approaches (other than TKIs), druggable target, and, therefore, new molecules that could effectively counteract the disease. KIT/PDGFRA oncogenic signaling is mainly sustained by two paths, the PI3K/mTOR and RAS/MAPK cascades, through direct interaction with PI3K and GRB2. These paths are activated regardless of the specific KIT/PDGFRA genetic alterations, and, more important, irrespective of imatinib responsiveness or resistance [7–9]. Activation of the PI3K/mTOR via is pivotal in GIST for its tumor initiation, development, and survival [10]. Preliminary preclinical reports on imatinib-sensitive and resistant GIST models showed pro-apoptotic and anti-proliferative activity after treatment with PI3K inhibitors, both as single treatment as well as in combination with imatinib [7–14].

Small molecule drugs, such as imatinib, have several distinctive advantages, including oral bioavailability, membrane permeability, intracellular target engagement, and versatility in mechanisms of action. In this context, the chemical structures of approved and previously investigated drugs were analyzed to identify novel promising molecules active against GISTs. Our efforts focused on a tricyclic aromatic scaffold, the benzo[d]imidazo[2,1-b]thiazole, which was selected for the construction of a small library of compounds (Figs. S1 and S2), given that the versatile benzo[d]imidazo[2,1-b]thiazole core allows for the insertion of various substituents. Interestingly, this core structure is present in several drugs including therapeutics with antitumor activity [15–21].

## Materials and methods

### Chemistry

Thin layer chromatography was performed on Bakerflex plates (Silica gel IB2-F), the eluent was a mixture of petroleum ether/acetone/methanol in various proportions. The ^1^H NMR and ^13^C NMR spectra were recorded on a Varian MR 400 MHz (ATB PFG probe) and on a Bruker ADVANCE NEO 600 MHz, equipped with liquid nitrogen chilled Bruker Prodigy (1H/19F)-X broadband probe. UHPLC − MS analyses were run on a Waters ACQUITY ARC UHPLC/MS system consisting of a QDa mass spectrometer equipped with an electrospray ionization interface and a 2489 UV/Vis detector. The detected wavelengths (λ) were 254 nm and 365 nm. The analyses were performed on an XBridge BEH C18 column (10 × 2.1 mm i.d., particle size 2.5 μm) with a XBridge BEH C18 VanGuard Cartridge precolumn (5 mm × 2.1 mm i.d., particle size 1.8 μm). The mobile phases were H_2_O (0.1% formic acid) (A) and MeCN (0.1% formic acid) (B). Electrospray ionization in positive and negative mode was applied in the mass scan range 50 − 1200 Da. Analyses were performed using a generic method with a linear gradient: 0–1.5 min, 40% B; 1,5–6 min, 95% B; 6–8 min, 95% B; 8–10 min, 40% B; Flow rate: 0.8 mL/min. The purities of all tested compounds, determined by UHPLC/MS were > 95%. Elemental analyses were within ± 0.4% of the theoretical values. Compounds were named relying on the naming algorithm developed by CambridgeSoft Corporation (Perkin Elmer) and used in Chem-BioDraw Ultra 14.0 (Perkin Elmer). All solvents and reagents, unless otherwise stated, were supplied by Aldrich Chemical Co. Ltd. and were used without further purification.

Synthetic procedures, yields, ^1^H NMR and ^13^C NMR and MS data for each compound are reported in Supplementary Data together with ^1^H NMR, ^13^C NMR and NOE spectra, and UHPLC − MS analyses.

### Cell cultures

Antitumoral activity of the 27 newly synthetized molecules was tested on four different GIST cell lines, GIST-T1, GIST-882, GIST-48, GIST-48B. GIST-T1 (RRID: CVCL_4976) was purchased from COSMO-BIO; it harbors an exon 11 heterozygous deletion (V560-Y579). GIST-48 (RRID: CVCL_7041), GIST-48B (RRID: CVCL_M441) and GIST-882 (RRID: CVCL_7044) were kindly donated by Dr. Fletcher (Harvard Medical School). GIST-48 harbors a primary mutation on KIT exon 11 (V560D) and an additional secondary mutation in the exon 17 (D820A); GIST-48B cells are derived from GIST-48 cells and share the same KIT mutational status, but have lost KIT expression under pharmacological pressure with an HSP-90 inhibitor; GIST-882 harbors a substitution in KIT exon 13 (K642E). GIST-T1 and GIST-882, defined as imatinib-sensitive, were grown in RPMI‐1640 supplemented with 10% FBS while GIST-48 and 48B (defined as imatinib-resistant, due to secondary resistance mechanisms) were grown in IMDM supplemented with 10% FBS. During the whole duration of the study, all lines were repeatedly credentialed by Sanger sequencing evaluations of known mutations. All cultures were shown to be mycoplasma-free. Peripheral blood lymphocytes (PBMCs) isolation was performed as previously described [22]. Authorization for the use of human blood samples (buffy coat) was obtained from AUSL of Bologna, Italy, S. Orsola-Malpighi Hospital -PROT GEN No. 0051937, and written informed consent was obtained from donors for the use of their blood for scientific research purposes. WS1 human fibroblast cell line (ATCC, Cat# CRL-1502, RRID: CVCL_2766) was used as a further adherent non-tumor model. Cells were grown in DMEM supplemented with D-glucose (4.5 g/L), 10% FBS, L-glutamine (2 mM); all media were supplemented with 1% penicillin–streptomycin solution and 1% Amphotericin B (Gibco). PBMCs, GISTs, and WS1 cell lines were incubated at 37 °C in 5% CO_2_ atmosphere.

### Cell viability assay

At day 0, GIST-T1 (5,000 cells/well), GIST-882 (10,000 cells/well), GIST-48 (10,000 cells/well), GIST-48B (10,000 cells/well) and WS1 cells (5,000 cells/well) were plated in a 96-well plate and cultured 24 h with complete medium. All the compounds were dissolved in DMSO just before cell treatment at day 1; cells were treated with increasing concentrations of pharmacological compounds (range: 10 nM-10 µM) and, after 72 h, cell viability was evaluated by MTT assay using a standard protocol. IC_50_ values were estimated using GraphPad Prism version 10.0.0 (RRID: SCR_002798).

IC_50_ values for MBT3T was then confirmed with CellTiter-Glo (CTG) Luminescent Cell Viability Assay (Promega). CTG reagent was added directly to the wells at a 1:1 volume ratio with the culture medium, the plate was shaken on an orbital shaker for 2 min and incubated in the dark for 10 min. Luminescent signals were recorded through GloMax Discover Promega luminometer. MBT3T was also tested on human peripheral lymphocytes: 100,000 cells/well were seeded in a 24-well plate and treated with different concentrations of the compound; after 72 h cell viability was assessed through the Guava ViaCount staining using a flow cytometry approach, as previously described [22]; imatinib, sunitinib, regorafenib were used as a comparison, as they represent standard targeted therapies for GIST while doxorubicin was used as a positive control for toxicity in lymphocytes.

### Western blotting assay

The cultured cells were lysed with NP40 buffer (Thermo Fisher) supplemented with protease inhibitor cocktail (Sigma-Aldrich). Protein lysates were separated by 4% to 20% SDS-PAGE before being transferred to the nitrocellulose membrane (Millipore) and incubated with specific antibodies. Actin antibody (1:5000; Sigma-Aldrich Cat# A1978, RRID: AB_476692) was used as a control. The primary antibodies used were KIT (1:1000; Agilent-Dako Cat#4502), phospo-KIT (CST Cat# 3391, RRID: AB_2131153), MAPK (CST Cat# 9102, RRID: AB_330744), phospo-MAPK (CST Cat# 9211, RRID: AB_331641), AKT (CST, Cat# 9272, RRID: AB_329827) phospo-AKT (CST, Cat# 9271, RRID: AB_329825). The hybridization signals were detected by chemiluminescence and captured using the ChemiDoc Imaging System (Bio-Rad).

### Human phospho-RTK array

To evaluate potential changes in the phosphorylation pattern between MBT3T treated and non-treated cells, the Proteome Profiler Human Phospho-RTK Array Kit (Biotechne, ARY001B) was used to determine the relative levels of tyrosine phosphorylation of 49 distinct RTKs, according to the manufacturer’s protocol. Protein lysates were prepared, and incubated with the array membranes as per the manufacturer’s instructions. Following the addition of chemiluminescent detection reagents, a signal proportional to the amount of protein bound was detected.

### Cell cycle analysis

The Muse Cell Cycle Reagent (Millipore) was used for the detection of cell cycle phase in which the cells are. Briefly, 1 × 10^6^ cells/well in a 6-well plate were treated with different concentrations of MBT3T (200 nM, 500 nM and 1 µM). Imatinib 1 µM (GIST gold standard treatment) was included as control. After 72 h, cells were fixed in 70% ethanol, and incubated with Muse Reagent in the dark for 30 min at 25 °C. The cell cycle was then analyzed on a Muse cell analyzer (EMD Millipore). All measurements were performed in triplicate.

### Apoptosis analysis

MBT3T pro-apoptotic action on GIST cell lines was assessed using the Annexin-V cytofluorimetric assay. Flow cytometry analysis was performed to identify the percentage of apoptotic cells using Guava Nexin Reagent (Catalog No. 4500 − 0450, Millipore). In brief 1 × 10^6^ cells/well in a 6-well plate were seeded and treated with different concentrations of MBT3T (200 nM, 500 nM and 1 µM), imatinib (1 µM), and staurosporine (1 µM) as positive control. After 48 h, cells were harvested, washed twice with PBS, and resuspended in 100 µL of kit buffer, then incubated at room temperature for 20 min in the dark. Apoptotic cells were analyzed using Guava EasyCyte flow cytometer (Guava Technologies). All measurements were performed in triplicate.

### Caspase activation assay

For the caspase activation detection, Caspase-Glo 3/7 assay (Promega) was used. Cells were plated in a 96-well plate (white bottom) at a density of 5,000 cells/well for GIST-T1 and 10,000 cells/well for GIST-882, GIST-48 and GIST-48B. Cells were treated with MBT3T at different concentrations (200 nM, 500 nM and 1 µM) and imatinib 1 µM; staurosporine 1 µM was used as positive control. After 48 h, the plate was removed from the incubator and allowed to equilibrate at room temperature, culture medium was removed, and 80 µL of Caspase-Glo 3/7 reagent was added to each well and incubated for 1.30 h. The luminescence signal, directly proportional to the activity of the caspases, was detected by the luminometer GloMax Discover Promega. The experiment was replicated three times.

### Immunofluorescence

Cells were seeded on 8-well chamber slides (Lab-Tek, Thermo Fisher Scientific) and allowed to adhere overnight (60,000 cells/well for GIST-T1 and 100,000 cells/well for GIST-48B). Cells were treated with MBT3T (1 µM), imatinib (1 µM) and colchicine (1 µM, Sigma-Aldrich, C9754) as a positive control to assess microtubule disruption; DMSO was included as negative control. 24 h after treatment, cells were fixed with 4% paraformaldehyde (PFA) in DPBS for 15 min at room temperature, followed by permeabilization with 0.1% Triton X-100 in DPBS for 10 min. Non-specific binding was blocked with 5% BSA in DPBS for 30 min. Cells were then incubated overnight at 4 °C with the FITC conjugate primary antibody α-tubulin III (1:500, Sigma-Aldrich, Cat. No. F2168, AB_476967) diluted in blocking solution. The day after, nuclei were counterstained with Hoechst (1 µg/mL, Thermo Fisher Scientific). Imaging was taken using a Nikon A1R+ high-resolution laser scanning confocal microscope, equipped with standard fluorescence filter sets and auxiliary modules for multi-channel acquisition. Images were acquired separately for each fluorescence channel and merged to visualize colocalization between the green (FITC) and blue (Hoechst) signals.

### 3D bioprinting

Formulation of optimized bioink was 3.5% w/v alginate, 4.6% w/v mannitol, and 4% w/v gelatin type B from bovine skin, dissolved in DPBS 1X, without CaCl_2_ and MgCl_2_. Sodium alginate (W201502), gelatin type B from bovine skin (G9391), D-mannitol (M4125) were purchased from Sigma-Aldrich (RRID: SCR_008988). After powders dissolvement, the mixture was pasteurized for 1 h at 72 °C and stirred until complete dissolution at room temperature. For crosslinker preparation, calcium chloride (C3306, Sigma-Aldrich, RRID: SCR_008988) was dissolved in sterile water at a final concentration of 100 mM.

The Cellink Inkredible 3D Bioprinter from the BICO Company was used. Square-shaped constructs with dimensions of 10 × 10 × 1.2 mm^3^ were printed with the previously described bioink directly on a 12-well plate. Particularly, cells from 2D cell cultures were harvested and 12.5 × 10^6^ cells were mixed with 1 mL of the alginate-based bioink in a 10:1 ratio (alginate-based bioink: GIST cells suspension). Models for 3D bioprinting were designed with Fusion360 Autodesk software (v.2.0, Autodesk); the procedure code for the printing process was elaborated by employing the open-source slicing software Slic3r (v.1.2.9, open source). Cell printing took place at 24 °C using a plastic tip with a diameter of 22 G at a pressure of 5–10 kPa. Crosslinking of the bioprinted model with 100 mM calcium chloride solution was performed and after quick washing with DPBS, culture medium was added in the wells. The following day, constructs were treated with different concentrations of MBT3T, imatinib, and DMSO as a control; for each condition, 3 different constructs were bioprinted.

The growth medium of 3D constructs was replaced every three days, with re-administration of the drug at each medium change, and constructs were maintained in culture for up to 14 days.

### Cell viability assay in 3D models

Cell viability in 3D models was assessed using Alamar Blue assay (Thermo Fisher Scientific). 10% of Alamar reagent was added to the culture medium in each well and incubated at 37 °C for 24 h. For each well, absorbance was then read in a Tecan plate reader at 570 nM (600 nM reference wavelength), in triplicate.

### Computational studies

#### *Structure similarity search*

The structural similarity search was carried out using the SwissSimilarity web platform. The web-server allows fast similarity searches against multiple chemical libraries using several 2D- and 3D-similarity metrics. MBT3T was uploaded as the query structure, and similarity screening was performed against the ChEMBL (RRID: SCR_014042) database, employing all available similarity metrics. Compounds were ranked by similarity score.

#### *Docking studies*

Molecular docking studies were performed using AutoDock (RRID: SCR_012746) Vina [23, 24].

The molecular structure of MBT3T was optimized prior to docking using the Hartree–Fock (HF) method with the 6-31G* basis set.

Docking calculations were carried out in triplicate, using an exhaustiveness value of 32 and an energy range of 100. Docking results were ranked according to the predicted binding energy and the most favorable pose was retained for subsequent simulations.

#### Molecular dynamic simulations

MBT3T, GTP and colchicine were parameterized following the standard AMBER protocol, which involves geometry optimization at the Hartree-Fock level with the 6-31G* basis set and derivation of restrained electrostatic potential (RESP) atomic charges. Ligand parameterization was performed using Antechamber, with atom types assigned according to the GAFF2 force field [25]; the tubulin protein instead was described using the ff14SB force field [26].

The top-ranked docking pose of MBT3T in the tubulin binding site was subjected to all-atom molecular dynamics (MD) simulations using the Amber22 package [27].

Prior to production runs, the systems underwent energy minimization consisting of 10,000 steps, performed in two phases: the first 5,000 steps using the steepest descent method, followed by 5,000 steps with the conjugate gradient algorithm. Subsequently, equilibration was carried out for 1 ns under constant temperature conditions, gradually increasing the system to 300 K with a Langevin thermostat.

Production MD simulations were run for 100 ns under periodic boundary conditions (PBC), applying particle mesh Ewald (PME) for long-range electrostatics with a 10 Å cutoff for non-bonded interactions.

The binding energies of MBT3T and colchicine in complex with tubulin were estimated using the Molecular Mechanics-Generalized Born Surface Area (MM-GBSA) method [28].

### Zebrafish model

#### *Danio rerio husbandry*

All experiments were performed on zebrafish (*Danio rerio*) (RRID: SCR_007282) embryos and larvae up to 5 days post-fertilization (dpf) in accordance with European Directive 2010/63/EU and Italian legislation on animal welfare. Wild-type zebrafish (Tübingen strain) were maintained at 28.5 °C under a 12 h light/dark photoperiod, following standard husbandry protocols described by Westerfield [29]. Feeding and rearing procedures were conducted according to established developmental guidelines [30]. For anesthesia and euthanasia, tricaine (MS-222) was applied to system water at final concentrations of 0.16 mg mL⁻¹ and 0.3 mg mL⁻¹, respectively.

#### *Xenotransplantation*

At 2 dpf, zebrafish embryos were anesthetized with 0.16 mg mL⁻¹ tricaine and positioned in plastic grooves filled with 2% methylcellulose in PBS. GIST-T1 (imatinib-sensitive) and GIST-48B (imatinib-resistant) cells were labeled for 20 min at 37 °C using Vybrant™ DiI Cell-Labeling Solution (5 µg/mL; Thermo Fisher Scientific), a lipophilic fluorescent dye that uniformly stains cell membranes. After labeling, cells were resuspended in 10 µL PBS at a concentration of 1 × 10⁵ cells/µL, loaded into glass capillary needles, and microinjected into the yolk sac of each embryo (approximately 200 cells per embryo) using a PV830 Pneumatic PicoPump and a micromanipulator (WPI). Following injection, embryos were maintained at 33 °C and monitored daily until 3 days post-injection (dpi), the experimental endpoint. At 1 dpi, embryos showing disseminated cells within blood vessels were excluded to ensure consistent xenograft localization and comparable tumor size across samples. Remaining embryos were randomly assigned to treatment groups and exposed to DMSO (vehicle control), imatinib (30 µM), or MBT3T (0.2 µM). Dead embryos were removed daily throughout the observation period.

#### *Birefringence assay*

Muscle birefringence, an indicator of myofibrillar organization, was analyzed in anesthetized zebrafish larvae mounted in 2% methylcellulose. Larvae were positioned on a glass polarizing filter and covered with a second filter on a Leica M165FC microscope equipped with a DFC7000T digital camera. The upper polarizer was rotated until the characteristic refraction of light through the striated muscle became visible, allowing optimal contrast. Images were captured in bright field, and pixel intensity within the trunk region was quantified using ImageJ software. To account for minor variations in larval size among treatment groups, birefringence values were normalized to the measured area of each sample. Two independent clutches of larvae per treatment were analyzed to ensure reproducibility.

#### *Locomotion assay*

Larval locomotor activity was evaluated using a DanioVision tracking system (Noldus Information Technology). At 5 dpf, individual larvae were transferred to 24-well plates, one larva per well, containing 1 mL of system water. Following a 20-minacclimation period, locomotor behavior was recorded through three consecutive cycles consisting of 10 min of light and 10 min of darkness, following procedures adapted from Brañas et al. [31].

#### *Imaging and data analysis*

High-resolution imaging of zebrafish larvae at 5 dpf was performed using a Leica SP8 confocal microscope. Larvae were mounted in 1% low-melting-point agarose for stabilization during image acquisition. Quantification of fluorescent signals within defined regions of interest (ROIs) was carried out using the open-source ImageJ/Fiji software, following previously established procedures [32]. A threshold was applied to exclude background fluorescence, and only signal-positive pixels above the cutoff were included in the analysis. Fluorescence intensities were normalized and expressed as arbitrary units (A.U.) to enable comparison across experimental replicates. Imaging parameters, including laser excitation power, detector gain, and acquisition settings, were kept constant for all samples to ensure data consistency.

### Statistical analysis

Statistical analyses were performed using GraphPad Prism (version 10). Data distribution was evaluated with the Shapiro–Wilk test for normality (α = 0.05). For normally distributed datasets, parametric tests were applied, including the unpaired Student’s t-test for two-group comparisons and one-way ANOVA for multiple groups. Data are presented as mean values with individual data points, and error bars represent the standard error of the mean (SEM).

## Results

### Synthesis of the new compounds

Synthesis and structural characterization of all the new compounds are described in detail in Supplementary Data. Synthetic schemes are described in Figs. S3 and S4, while the NOE connections determined by 1D-NOESY experiments are described in Fig. S5.

### Cytotoxicity assay

**Fig. 1:**
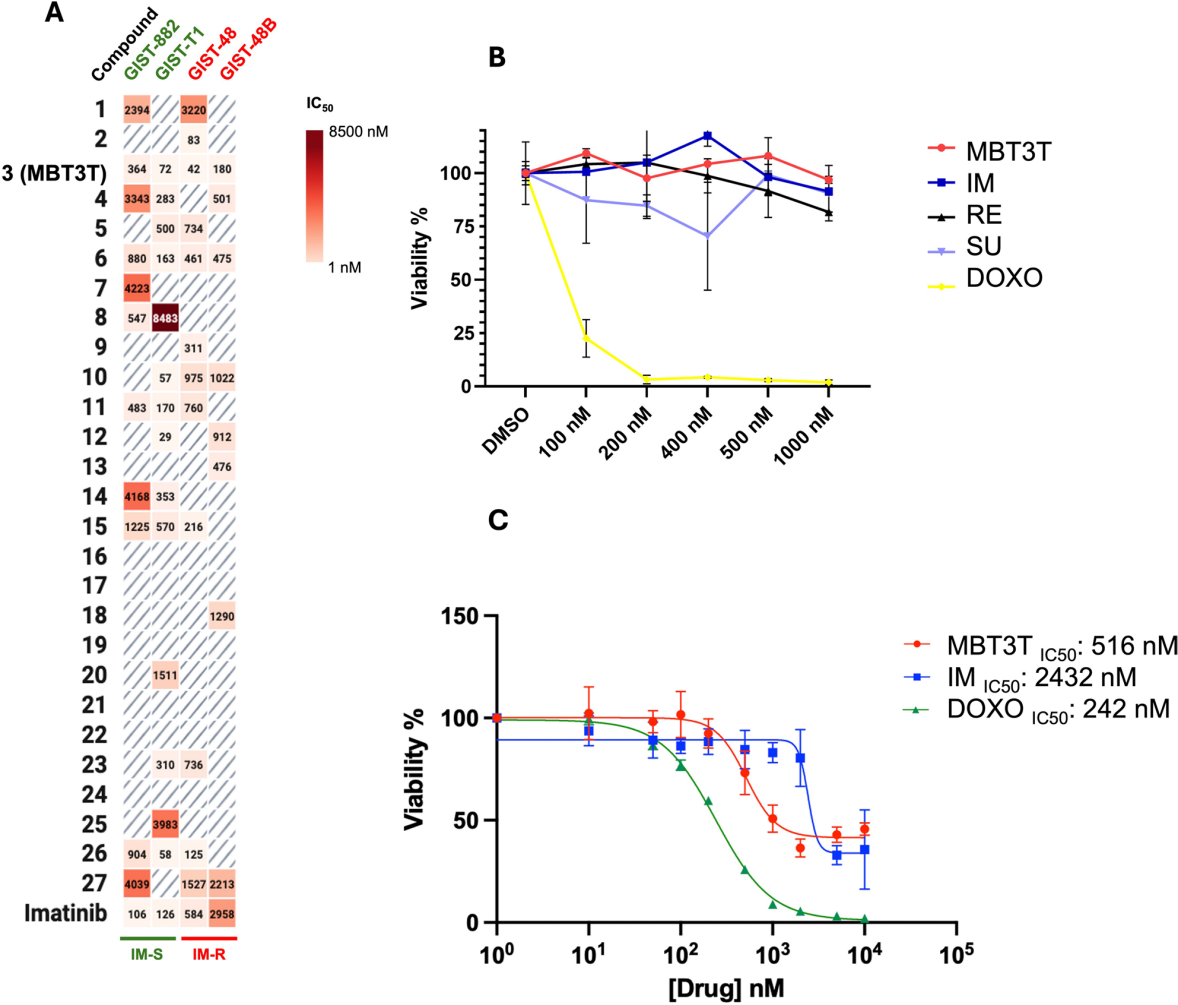
**A** Summary of the results obtained for treatment of GIST cell lines with 27 novel pharmacological compounds. Data are expressed as IC_50_ (nM, concentration inhibiting 50% of cell growth): not effective; IM-S: imatinib-sensitive; IM-R: imatinib-resistant. **B** PBMCs viability after 72 h-treatment with MBT3T, the three approved drugs [imatinib (IM), sunitinib (SU), regorafenib (RE)] and doxorubicin (DOXO) as positive control. **C** Viability after 72 h-treatment with MBT3T, imatinib (IM), and doxorubicin (DOXO) in WS1 cell line

Cytotoxic activity of compounds 1–27 was evaluated after 72 h of treatment through the MTT assay on four different GIST cell lines (GIST-T1 and GIST-882, imatinib-sensitive, GIST-48 and GIST-48B, imatinib-resistant); IC_50_ values are reported in Fig. 1A. Among the tested compounds, the most promising results were obtained with compound 3 (hereafter indicated as MBT3T), which proved to be active against all four GIST cell lines, with IC_50_ values in the nanomolar range. The lack of the methoxy group (compound 1) or the shift to the 5 position (compound 2) led to a drop of activity. Similarly, the substitution of the methoxy group with chlorine (compound 4) or fluorine (compound 5) resulted in a decrease in activity, especially for the chlorine derivative. Based on these results, the methoxy group at position 7 seems to play a key role in determining the cytotoxic effect against GIST cells. However, even the aromatic ring at position 2 appears to influence the activity with the 3-thienyl group, giving the best results. Indeed, while less active than MBT3T, also compounds 2, 4–6, showed some activity against GIST cells. Interestingly, the substitution of the 3-thienyl with a 2-thienyl group or with the bioisostere phenyl, while maintaining the methoxy group at position 7, (compound 10 and 11 respectively), resulted in a loss of activity, especially against GIST-882, GIST-48 and GIST-48B cells, thus suggesting a very specific target interaction.

Based on these results, MBT3T was selected for further studies. Results of MBT3T cytotoxic effect were confirmed with the more sensitive CTG Cell Viability Assay (calculated IC_50_: 188.7 nM, 80.93 nM, 100.5 nM and 233 nM for GIST-882, GIST-T1, GIST-48 and GIST-48B, respectively), Fig. S6.

### Evaluation of MBT3T toxicity on PBMCs and fibroblasts

To assess the specificity and selectivity of MBT3T in GIST cells, we evaluated its toxicity on PBMCs, using TKIs as negative controls and doxorubicin, a well-known unspecific (i.e. not targeted drug) chemotherapy agent, as positive control. PBMCs showed a viability of 96.9% when treated with the highest concentration (i.e. 1000 nM) of MBT3T, similar to imatinib, sunitinib and regorafenib; on the contrary, as expected, doxorubicin showed a strong toxicity already at the lowest concentration of 100 nM **(**Fig. 1B). Activity of MBT3T was additionally evaluated against the WS1 human fibroblast cell line. As shown in Fig. 1C, doxorubicin induced mortality in 50% of cells at a concertation of about 240 nM. MBT3T and imatinib respectively, required more than 500 nM and 3000 nM to achieve the same effect. This was expected for imatinib, considering that normal cells usually do not express KIT at a high level, as GISTs do.

### Evaluation of the KIT signaling cascade and RTK profile

To shed light on the molecular mechanisms beyond the MBT3T effect, we first investigated the KIT downstream signaling cascade. However, we could not observe any consistent inactivation for KIT downstream cascade (Fig. S7). We also evaluated the RTK profile, however, besides the expected phosphorylated RTKs as KIT, we did not observe any alteration after treatment with MBT3T (Fig. S8). This prompted us to infer that MBT3T had a different target.

### Computational studies

**Fig. 2:**
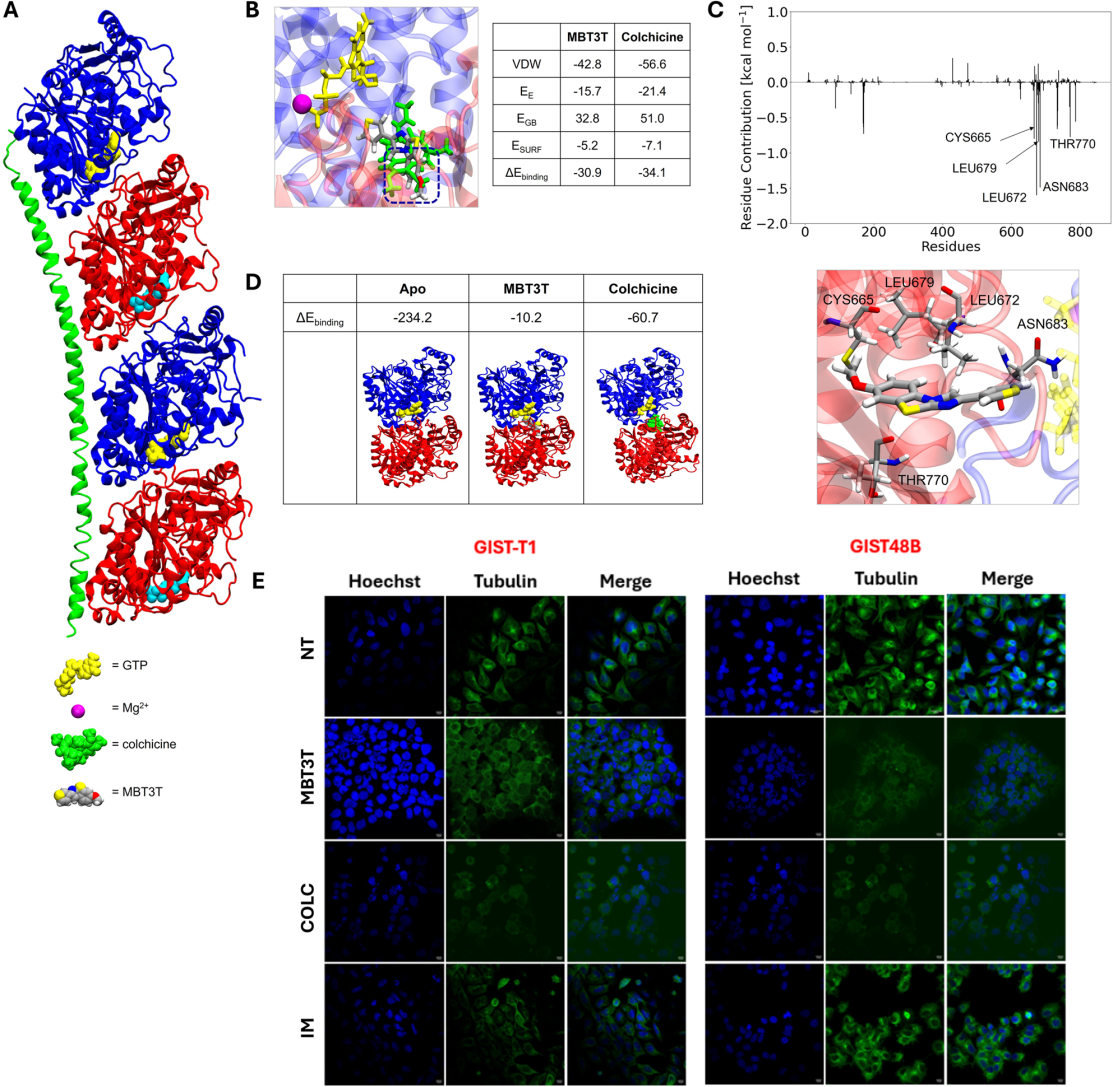
Target prediction and validation. **A** Crystal structure of tubulin (PDB ID: 3E22): α units in blue, β units in red, stathmin-like domain in green, GTP in yellow, GDP in cyan and Mg^2+^ in magenta. **B** Close-up of binding pocket at the α/β interface and superimposition of MBT3T with colchicine (in green); energetic terms of computed binding energy. **C** Fingerprint of protein residue contributions to MBT3T binding on top; detailed 3D representation of most interacting residues with MBT3T on the bottom. **D** Binding affinity for α/β complex formation in the apo system, in the presence of MBT3T and colchicine. **E** Effect of compounds on the microtubule network, assesed by immunofluorescence. GIST-48B and T1 cells, as example, were treated with MBT3T, colchicine and imatinib at 1 µM for 24 h. Physiological microtubule organization can be observed in green in untreated cells (NT); this was found to be disrupted in cells treated with MBT3T. COLC: colchicine; IM: imatinib

To identify the potential protein targets of MBT3T, a ligand-based in silico similarity analysis was carried out using the SwissSimilarity tool [33]. This analysis identified a high degree of structural similarity (similarity index > 0.96) between MBT3T and a series of known bioactive molecules (Fig. S9). Notably, the compound that displayed the highest similarity score (0.992), shares the benzo[d]imidazo[2,1-b]thiazole core with MBT3T and functions as an inhibitor of tubulin polymerization [21]. To evaluate this hypothesis, computational studies were performed. Molecular docking studies on the crystal structure of tubulin (PDB ID: 3E22, Fig. 2A) revealed that MBT3T shares the same binding site of colchicine, a well-known inhibitor of tubulin polymerization **(**Fig. 2B). Molecular dynamic (MD) simulations, followed by an estimation of the binding energies, using MM-GBSA calculations, showed that the binding affinity (ΔE_binding_) of MBT3T to the colchicine-binding site of tubulin is -30.9 kcal mol^− 1^. The ΔE_binding_ of colchicine is -34.1 kcal mol^− 1^ suggesting that MBT3T binds to tubulin, with an affinity comparable to that of colchicine. A per-residue energy decomposition analysis was performed to elucidate the individual contributions of protein residues to ligand binding (Fig. 2C). The results indicate that the interaction of MBT3T with the binding site is predominantly stabilized by non-polar residues, including Leu672 and Leu679, which engage in hydrophobic contacts with the aromatic benzo[d]imidazo[2,1-b]thiazoles core of the ligand (-1.6 and − 0.8 kcal mol^− 1^, respectively). Asn683 is responsible for anchoring the 3-thienyl group by CH-π type interactions (-1.5 kcal mol^− 1^). Additionally, Cys665 and Thr770 contribute both with − 0.8 kcal mol^− 1^ through hydrogen bonding interactions, specifically involving the sulfur atoms of the thiazole ring and the methoxy oxygen at the R_3_ position of MBT3T. A structural superimposition of MBT3T and colchicine (Fig. 2B) shows that the methoxy group of MBT3T overlaps with the corresponding methoxy group of colchicine. These findings highlight the strategic importance of the OCH₃ group and the 3-thienyl substituent in conferring biological activity to MBT3T.

To assess whether MBT3T could effectively inhibit tubulin polymerization, MD simulations were performed to evaluate the stability of the α/β-tubulin dimer interface in physiological condition or in complex with colchicine or MBT3T. In physiological conditions, the binding between the α and β tubulin, in the dimeric structure is -234.2 kcal mol^− 1^, a value consistent with the typical tendency of tubulin to polymerize (Fig. 2D). In contrast, the presence of colchicine significantly weakens the α/β interface interaction, with a ΔE_binding_ of -60.7 kcal mol^− 1^, in line with its known mechanism of destabilizing microtubule polymerization. Strikingly, the tubulin dimer in the presence of MBT3T showed an even more pronounced destabilization, with an α/β binding energy of only − 10.2 kcal mol^− 1^.

### Effect of MBT3T on tubulin organization

Based on the computational findings, experimental validation was undertaken to confirm the predicted inhibitory effect of MBT3T on tubulin polymerization.

As shown in Fig. 2E, MBT3T induced tubulin disassembly after 24 h of treatment at 1 µM, in both imatinib-sensitive (GIST-T1) as well as imatinib-resistant (GIST-48B) cell lines, similarly to the effect of colchicine. As expected, this effect is not observed in cells treated with imatinib.

### MBT3T induced G2/M arrest in GIST cells

**Fig. 3:**
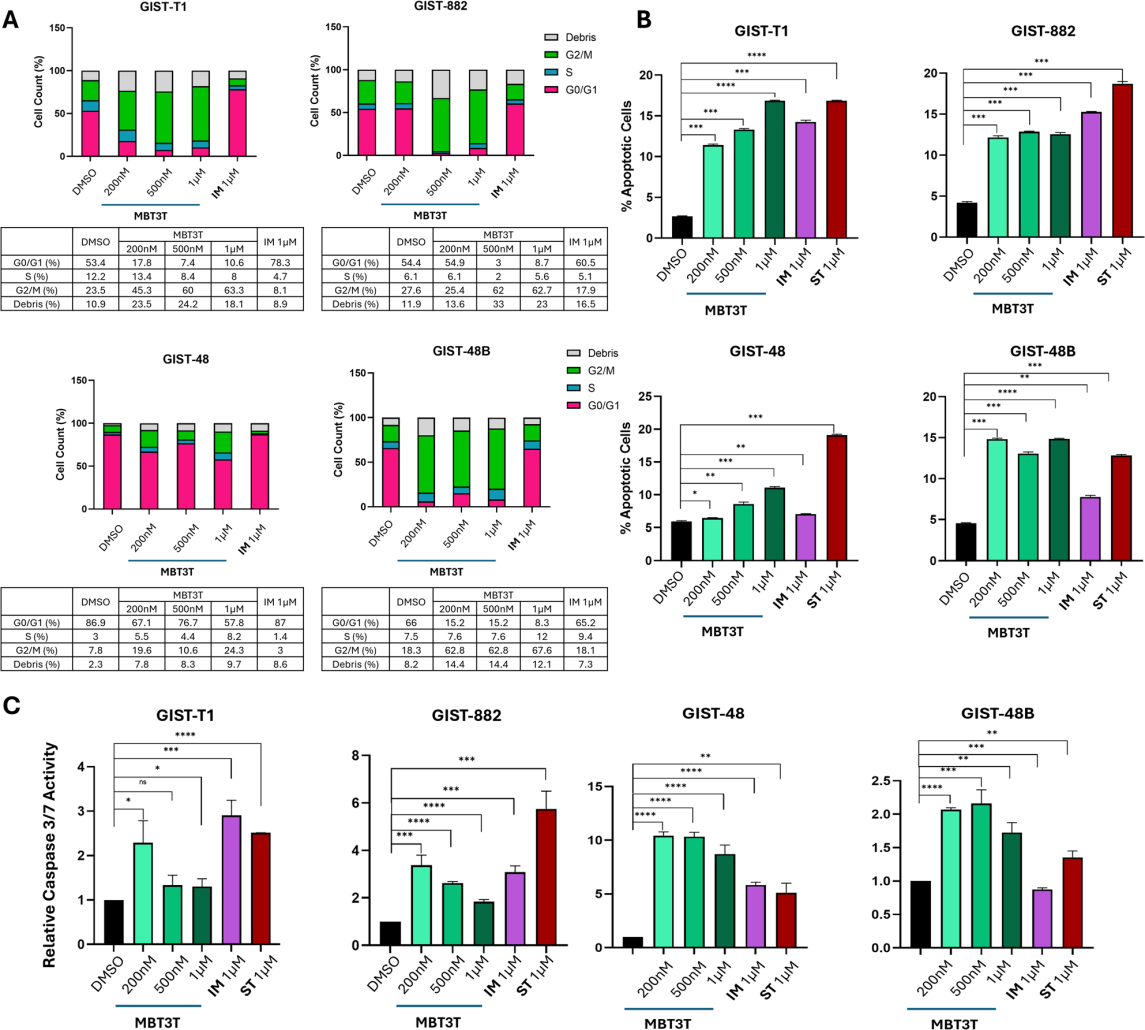
Evaluation of MBT3T effect on cell cycle and apoptosis. **A** Percentage of cells in each cell cycle phase after 72 h-treatment with increasing concentrations of MBT3T and imatinib (IM). **B** Percentage of apoptotic cells after 48 h-treatment with increasing concentrations MBT3T, imatinib (IM) and staurosporine (ST; as positive control). Columns, mean (*n* = 3) **C**. Relative caspase 3/7 activation, normalized on DMSO, after 48 h-treatment with different concentrations of MBT3T, imatinib (IM) and staurosporine (ST; as positive control). Columns, mean (*n* = 3). *: *p* < 0.05; **: *p* < 0.01; ***: *p* < 0.001; ****: *p* < 0.0001

To further demonstrate the effect of MBT3T on tubulin, we investigated the effect of MBT3T on cell cycle progression in GIST cell lines by flow cytometric assay. Indeed, it is well known that tubulin inhibitors cause G2/M phase arrest by disrupting the formation and function of mitotic spindles [34]. The cell cycle distribution was assessed after 24 h of treatment with increasing concentrations of MBT3T and the gold standard imatinib. As shown in Fig. 3A, imatinib treatment, in agreement with available literature, promoted an increase in the percentage of cells in G0/G1 phase. On the contrary, treatment with MBT3T led to accumulation of cells in G2/M phase. This was observed in all four treated GIST cell lines with, in particular, three of them (GIST-T1, GIST-882 and GIST-48B) characterized by more than 60% of cells in G2/M phase, when treated at the highest concentration. In addition, results showed that cell cycle arrest at G2/M phase was dose-dependent in GIST-T1 and GIST-882. Indeed, in the process of increasing the concentration from 200 nM to 1 µM, the proportion of G2/M phase in GIST-T1 and GIST-882 cells increased from 45.3% to 63.3%, and 25.4 to 62.7%, respectively.

### MBT3T induced apoptosis via caspase activation in GIST cells

Apoptosis is a common outcome of cell cycle arrest; therefore, we further evaluated apoptosis induction in GIST cells treated for 48 h with increasing concentrations of MBT3T by flow cytometric assay. As shown in Fig. 3B, MBT3T significantly induced apoptosis in all GIST cell lines, compared to DMSO. In particular, the apoptotic proportion of GIST-T1 and GIST-48 cells in the MBT3T-treated groups increased from 11.40% to 16.85% and 6.45% to 11.10%, respectively, indicating a dose-dependent effect in both cell lines. A significant effect of MBT3T on apoptosis was also observed in both GIST-882 and GIST-48B cell lines, with apoptotic cell percentages of 12.15% and 14.8%, respectively, even at the lowest concentration. Notably, MBT3T induced an even greater effect in GIST-48B cells compared to staurosporine, used as positive control. To confirm induction of apoptosis by MBT3T, caspase 3/7 activation after 48 h was assessed, using imatinib as comparison and staurosporine as positive control for apoptosis induction. As shown in Fig. 3C, MBT3T significantly induced caspase 3/7 activation in all cell lines at concentrations as low as 200 nM, confirming the findings from flow cytometric analysis.

### Efficacy of MBT3T on 3D bioprinted GIST models

Preclinical studies are a critical step in bridging the gap between scientific discovery and clinical application, but their predictive power is often insufficient. The FDA Modernization Act 2.0 aimed to reduce reliance on animal models by encouraging the adoption of alternative preclinical testing methods. In this context, 3D bioprinting is an emerging technique for advanced drug screening, that could pave the way for a reduction in animal tests [35].

For this reason and to better recapitulate the efficacy of MBT3T, we evaluated it on 3D bioprinted GIST models.

**Fig. 4:**
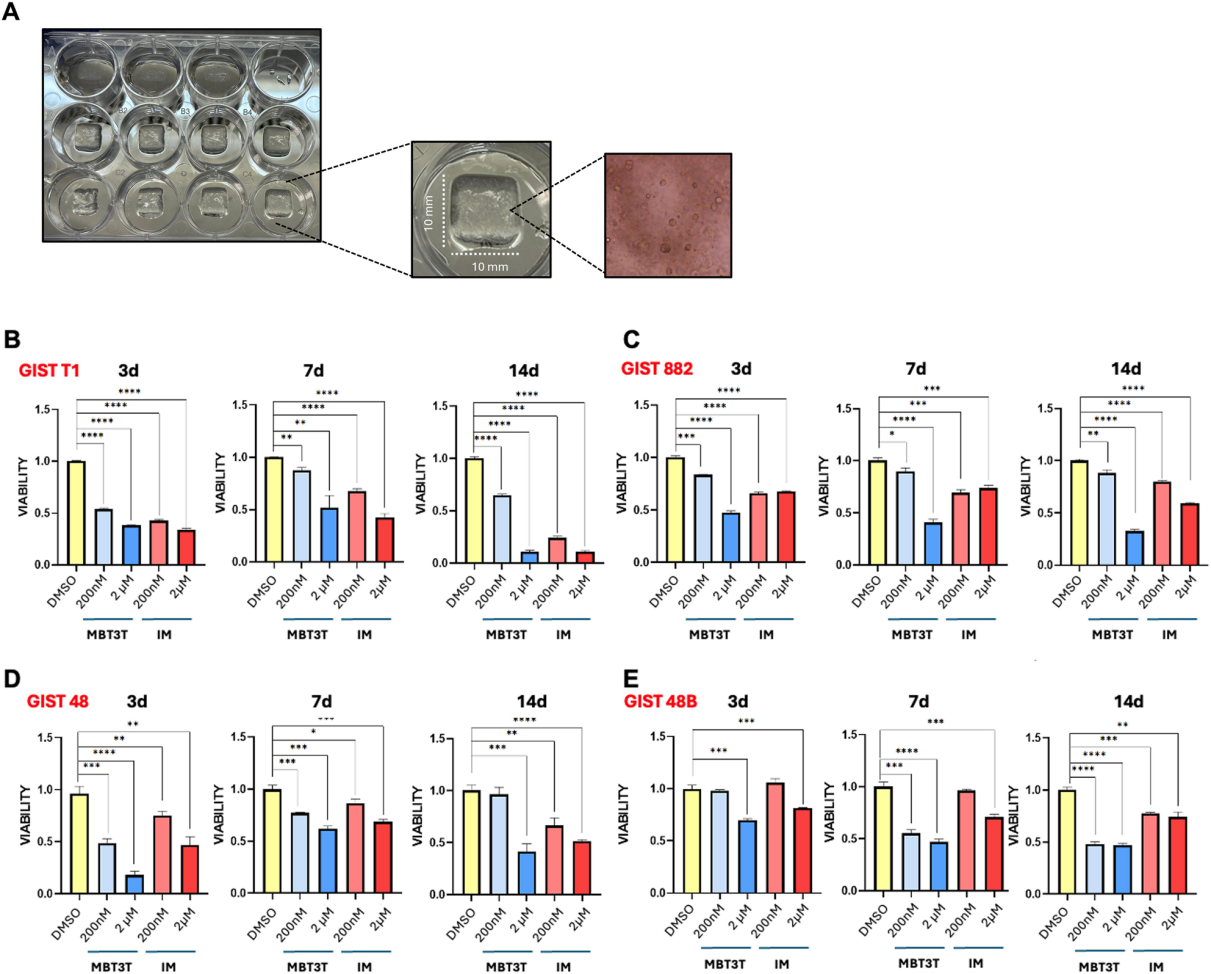
3D bioprinted GIST models.** A** Examples of a bioprinted model obtained for GIST-T1. Under a low-power light microscope with a 20X magnification, the 3D bioprinted GIST models remained stable for up to 14 days. On the right, the panel shows a detail of GIST-T1 cells within the bioink after 14 days. **B-E** Viability of 3D bioprinted GIST models treated with MBT3T and imatinib (IM) for 3, 7 and 14 days. MBT3T proved to be effective in both imatinib-sensitive, **B **GIST-T1 and **C** GIST-882 as well as in imatinib-resistant, **D** GIST-48 and **E** GIST-48B cell lines. Data are presented as mean ± SEM (*n* = 3). *: *p* < 0.05; **: *p* < 0.01; ***: *p* < 0.001; ****: *p* < 0.0001

As an example, Fig. 4A shows the bioprinted model obtained for GIST-T1. Under a low-power light microscope, the 3D bioprinted GIST models remained stable from days 1 to 14. This made us able to test MBT3T on GIST cells for a longer time (i.e. up to 14 days) compared with the 2D models that can be cultured for a shorter time.

We evaluated the viability of GIST-T1, GIST-882, GIST-48 and GIST-48B 3D cell models after 3, 7 and 14 days of incubation with MBT3T and imatinib at 200 nM and 2 µM to assess the model responses to antitumor medications (Fig. 4B-E). As expected by previous works on other cancer types, cells in 3D models tend to be more resistant to pharmacological treatments [36].

Typically, in 3D models, including spheroids or organoids, increased IC_50_ values and drug resistance are commonly seen; this phenomenon has been ascribed to multiple mechanisms, including a reduced penetration of drugs, higher pro-survival signaling, and/or overexpression of genes involving drug resistance. This highlights that evaluation of pharmacological response in conventional monolayer cultures may not reflect pathophysiological events occurring in cancer patients suggesting that screening in the 3D format may reveal information not accessible in standard 2D experiments [37].

In agreement with that, we did not observe a 50% reduction in viability when using a concentration of 200 nM for both MBT3T and imatinib, while we detected a significant decline of viability by applying 2 µM. Besides this, comparison of MBTB3T and imatinib showed the same trend observed in 2D, that is a higher efficacy of MBT3T over imatinib.

### Safety and tumor specificity response in zebrafish

**Fig. 5:**
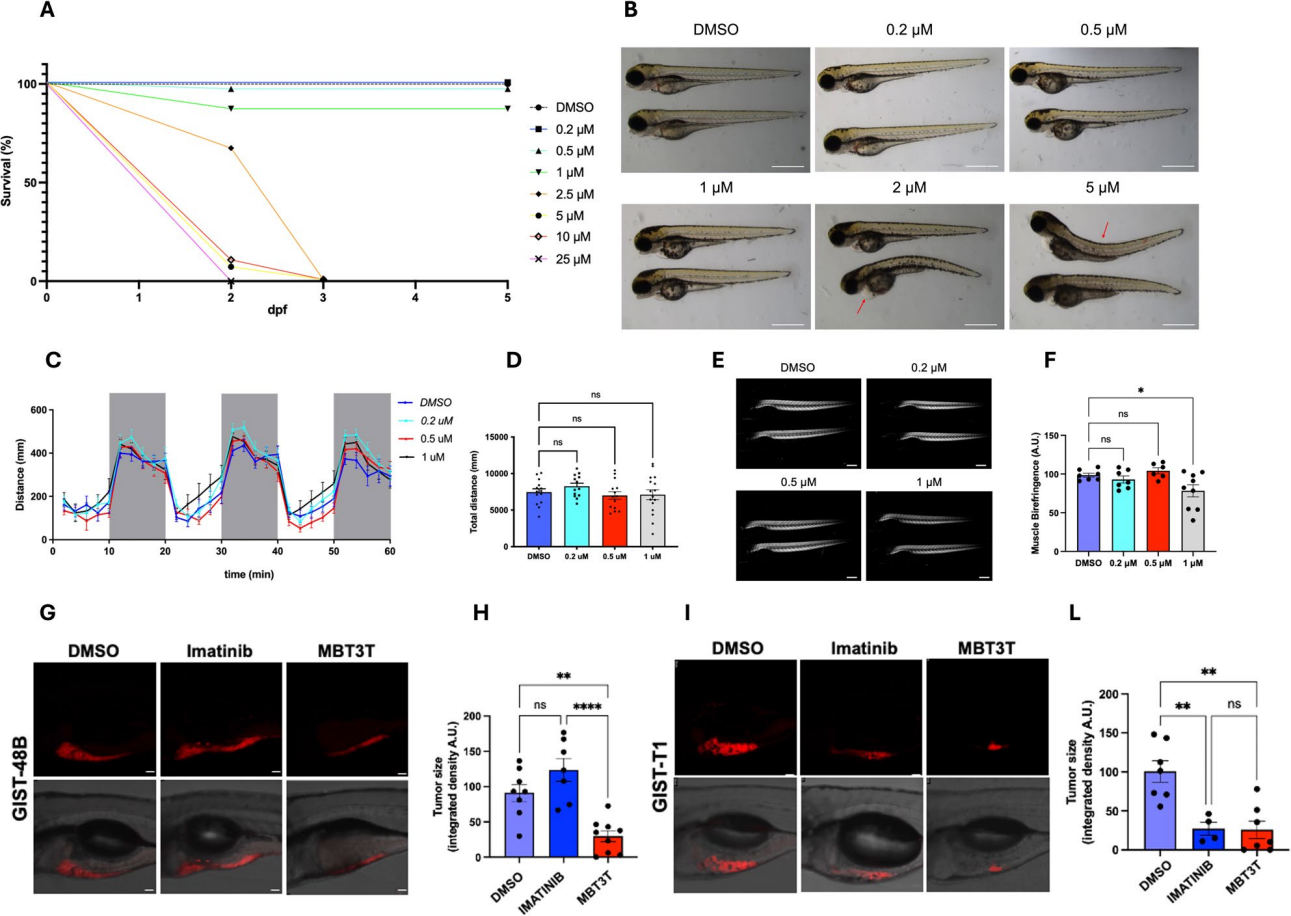
Studies on the zebrafish model. **A** Survival of zebrafish larvae exposed to increasing concentrations of MBT3T (0.2–25 µM) or vehicle control (DMSO) from 2 to 5 dpf. MBT3T induced a dose-dependent decrease in survival, with complete lethality observed from 2.5 µM within three days of treatment. **B** Morphological alterations in zebrafish larvae at 5 dpf exposed to increasing concentrations of MBT3T. Embryos were treated with vehicle (DMSO) or the indicated concentrations (0.2–5 µM) of the MBT3T from 2 dpf to 5 dpf. Control larvae (DMSO) displayed normal body morphology, whereas exposure to > 1 µM resulted in progressive defects including pericardial and yolk sac edema, body curvature (red arrows). Two representative larvae per condition are shown. Scale bar: 1 mm. **C** Locomotor activity profiles of larvae treated with vehicle (DMSO) or increasing concentrations of MBT3T (0.2, 0.5, and 1 µM) at 5 dpf. White areas represent light exposure periods, while grey areas indicate dark phases. No significant differences in movement were observed across the tested MBT3T concentrations. **D** Quantification of the total distance swum over 60 min. DMSO (*n* = 14), 0.2 µM (*n* = 14), 0.5 µM (*n* = 14), 1 µM (*n* = 16) larvae. Values represent mean ± SEM from three independent biological replicates. Statistical significance was determined by one-way ANOVA followed by Tukey’s post-hoc test; ns, not significant. **E** Representative birefringence images showing the structural integrity of skeletal muscles in zebrafish larvae (5 dpf) following treatment with increasing drug concentrations (DMSO, 0.2 µM, 0.5 µM, and 1 µM). The birefringence intensity reflects the degree of muscle fiber organization and polarization. Scale bar = 0.5 mm. **F** Quantification of normalized birefringence intensity (arbitrary units, A.U.). Statistical analysis revealed a significant decrease in birefringence at 1 µM compared to control (DMSO (*n* = 7), 0.2 µM (*n* = 7), 0.5 µM (*n* = 6), 1 µM (*n* = 9)). Bars represent mean ± SEM. **G-L** Zebrafish xenografts were established by injecting human **(G, H)** GIST-48B (imatinib-resistant) or **(I, L)** GIST-T1 (imatinib-sensitive) cells labelled with Vybrant™ DiI into the yolk sac of 2 dpf zebrafish embryos (0 days post-injection, dpi). At 4 dpf (2 dpi), larvae were treated with vehicle (DMSO), imatinib (30 µM), or MBT3T (0.5 µM) for 3 days. (G, I) Representative fluorescence and merged bright-field images show the tumor mass in GIST-48B and GIST-T1 xenografts following the different treatments. Scale bar: 50 μm. **H**, **L** Quantification of tumor size expressed as integrated fluorescence density (arbitrary units, A.U.). GIST-48B: DMSO (*n* = 8), imatinib (*n* = 7), MBT3T (*n* = 9) and GIST-T1: DMSO (*n* = 7), imatinib (*n* = 4), MBT3T (*n* = 7) larvae. Data are shown as mean ± SEM; statistical significance was determined by one-way ANOVA followed by Tukey’s post-hoc test; *: *p* < 0.05; **: *p* < 0.01; ****: *p* < 0.0001; ns: not significant

To evaluate the in vivo activity and tolerability of MBT3T, zebrafish (Danio rerio) embryos were employed as a vertebrate model system. We first assessed biocompatibility by exposing embryos to a concentration range from 0.2 µM to 10 µM of MBT3T. As shown in Fig. 5A, survival curves revealed that the compound was well tolerated at low doses, with no significant differences compared with DMSO controls up to 0.5 µM. A gradual decline in viability appeared at 1 µM, and complete lethality occurred above 2.5 µM.

To further characterize these effects, morphological evaluation revealed that larvae exposed to 0.2 or 0.5 µM developed normally, maintaining the typical straight body. In contrast, concentrations above 1 µM produced progressive malformations such as pericardial and yolk-sac edema, curvature of the trunk, and overall growth delay, confirming a dose-dependent developmental sensitivity (Fig. 5B). Next, larval locomotor behavior under alternating light and dark cycles using the DanioVision tracking system was analyzed. Individual larvae were placed in separate wells of a 24-well plate containing 1 mL of system water, and their movement was continuously recorded to quantify locomotor activity. Zebrafish typically exhibit increased swimming activity during darkness, which serves as a robust indicator of neuromuscular coordination. Larvae exposed to 0.2 µM and 0.5 µM MBT3T behaved indistinguishably from controls, and even at 1 µM no statistically significant differences were observed in the total distance moved (Fig. 5C-D). These results indicate that the tested concentrations were non-toxic and did not impair neuromuscular function.

To assess whether MBT3T could effectively inhibit tubulin polymerization in an in vivo model, muscle birefringence, a sensitive indicator of microtubule and sarcomeric alignment, was analyzed and observed that control larvae displayed a continuous bright pattern along the somites, whereas exposure to 1 µM MBT3T resulted in a uniform reduction in birefringence intensity, indicating microtubule depolymerization consistent with the compound’s proposed mechanism of action (Fig. 5E-F**)**. Finally, to assess the tumor-specific activity of MBT3T in vivo, zebrafish embryos were xenografted with fluorescently labeled human GIST-T1 (imatinib-sensitive) and GIST-48B (imatinib-resistant) cells and treated with either 0.2 µM MBT3T or 30 µM imatinib. After three days of treatment, no significant change in tumor size was observed in GIST-48B xenografts treated with imatinib, consistent with their resistant phenotype, whereas a clear reduction in tumor fluorescence was detected in GIST-T1 larvae treated with imatinib and in both xenograft models treated with MBT3T. These results indicate that MBT3T effectively reduces tumor growth in vivo, including in imatinib-resistant GIST cells (Fig. 5G-L).

Overall, these results demonstrate that MBT3T is well tolerated at sub-micromolar concentrations and retains strong, tumor-selective activity in vivo through disruption of microtubule organization.

## Discussion

The development of resistance to TKIs, particularly imatinib, remains one of the major unmet clinical challenges in the management of GISTs. Although successive lines of TKIs have improved patient outcomes, their efficacy is often limited by the emergence of secondary resistance mechanisms and by the persistent dependence on KIT/PDGFRA signaling inhibition [38]. In this context, the identification of therapeutic agents with mechanisms of action distinct from TK inhibition may represent a highly relevant strategy.

In the present study, we investigated a novel series of benzo[d]imidazo[2,1-b]thiazole derivatives and identified MBT3T as a potent antitumor compound with activity against both imatinib-sensitive and imatinib-resistant GIST models. In particular, MBT3T exhibited nanomolar cytotoxicity across four biologically distinct GIST cell lines, preserving its antitumor activity in more complex experimental systems, such as 3D bioprinted GIST models and zebrafish xenografts, underscoring the robustness of its biological effect.

Mechanistically, our data demonstrate that MBT3T exerts its antitumor activity independently of the canonical KIT signaling pathway. Neither KIT phosphorylation nor downstream PI3K/AKT and MAPK signaling appeared affected by treatment, prompting the investigation of alternative molecular targets. Ligand-based similarity analysis, molecular docking, and molecular dynamics simulations converged on tubulin as the most plausible target, with MBT3T binding to the colchicine site at the α/β-tubulin interface. Experimental validation confirmed that MBT3T induces microtubule disassembly, leading to G2/M cell cycle arrest and caspase-dependent apoptosis in both imatinib-sensitive and -resistant GIST cells lines.

MBT3T shares the mechanism of action with colchicine, a clinically approved microtubule inhibitor; however, while colchicine is a well-characterized tubulin polymerization inhibitor, its clinical application in oncology is severely limited by its narrow therapeutic window and dose-limiting systemic toxicity, particularly affecting gastrointestinal, neuromuscular, and hematological systems [39]. In contrast, MBT3T demonstrated a markedly favourable selectivity profile, showing minimal toxicity toward PBMCs and human fibroblasts at concentrations substantially higher than those required to inhibit GIST cell growth. Furthermore, MBT3T was well tolerated in vivo at sub-micromolar concentrations in zebrafish, without significant effects on survival, locomotor activity, or muscle integrity at therapeutically active doses. Moreover, unlike colchicine, which exerts broad cytotoxic effects across proliferating cell types (see also Fig. S10), MBT3T showed preferential activity against GIST cells, including models that are intrinsically resistant to TKIs. This selectivity may reflect subtle differences in binding mode, intracellular accumulation, or microtubule dynamics in GIST cells, and suggests that MBT3T could offer a wider therapeutic margin than classical microtubule inhibitors. Interestingly, target fishing analysis performed using the SwissTargetPrediction platform identified two additional potential molecular targets for MBT3T in GIST: PDGFRA, which is reported to be overexpressed by approximately 38,000-fold in GISTs, and EGFR, which shows approximately threefold overexpression. Compared with other chemotherapeutic agents, interactions of MBT3T with these receptors may induce selective uptake of MBT3T in GIST cells [40]. Finally, the benzo[d]imidazo[2,1-b]thiazole scaffold provides opportunities for further medical chemistry optimization, potentially enabling the development of derivatives with enhanced selectivity.

MBT3T treatment induced caspase 3/7 activation in all GIST cell lines, confirming that apoptosis represents a key component of its antitumor activity. Although the extent of caspase activation differed among the tested models, these variations were generally consistent with the heterogeneous biological background of GISTs and did not contradict the overall cytotoxic profile observed in viability assays. Differences in genetic context, baseline apoptotic susceptibility, and intracellular stress responses may contribute to the quantitative variability in caspase activation among cell lines [41–44]. Nevertheless, the ability of MBT3T to consistently trigger apoptotic signaling across both imatinib-sensitive and -resistant models further supports its mechanism of action as a non–KIT-centered therapeutic strategy capable of overcoming diverse resistance phenotypes.

Despite the promising results, this study has some limitations. First, while zebrafish xenografts provide a valuable in vivo platform for rapid efficacy and toxicity assessment, validation in mammalian models will be necessary to fully characterize the pharmacokinetic and pharmacodynamic properties of MBT3T, together with long-term toxicity, bioavailability, and potential off-target effects.

## Conclusion

Based on our results, MBT3T represents a novel, potent, and selective tubulin polymerization inhibitor with robust activity against both imatinib-sensitive and -resistant GISTs. Its distinct mechanism of action offers a promising therapeutic avenue for patients who have developed resistance to existing TKI therapies. Future studies will focus on comprehensive pharmacokinetic and pharmacodynamic characterization, as well as on exploring potential combination therapies to maximize its clinical benefit. The identification of MBT3T provides a compelling new drug candidate and underscores the value of exploring diverse molecular targets to overcome drug resistance in GIST therapy.

## Supplementary Information

Supplementary Material 1.

## Data Availability

Research data are available at (https://doi.org/10.6092/unibo/amsacta/8026).

## Abbreviations

3D: Three-dimensional
A.U.: Arbitrary Units
BSA: Bovine Serum Albumin
COLC: Colchicine
CTG: CellTiter-Glo (Luminescent Cell Viability Assay)
DMSO: Dimethyl sulfoxide
dpf: Days post-fertilization
dpi: Days post-injection
DOXO: Doxorubicin
FBS: Fetal Bovine Serum
FDA: Food and Drug Administration
FITC: Fluorescein isothiocyanate
GDP: Guanosine diphosphate
GISTs: Gastrointestinal stromal tumors
GTP: Guanosine triphosphate
HF: Hartree–Fock (method)
IC₅₀: Half maximal inhibitory concentration
IM: Imatinib
IM-R: Imatinib-resistant
IM-S: Imatinib-sensitive
MBT3T: Benzo[d]imidazo[2,1-b]thiazole derivative (compound 3)
MD: Molecular dynamics
MeCN: Acetonitrile
MM-GBSA: Molecular Mechanics-Generalized Born Surface Area
MTT: 3-(4,5-dimethylthiazol-2-yl)-2,5-diphenyltetrazolium bromide
NMR: Nuclear Magnetic Resonance (¹H-NMR: Proton; ¹³C-NMR: Carbon-13)
NOE: Nuclear Overhauser Effect
NT: Non-treated (control)
PBC: Periodic boundary conditions
PBMCs: Peripheral blood mononuclear cells
PDB: Protein Data Bank
PDGFRA: Platelet-derived growth factor receptor alpha
PFA: Paraformaldehyde
PME: Particle mesh Ewald
RE: Regorafenib
RTK: Receptor Tyrosine Kinase
SAR: Structure-activity relationship
SEM: Standard error of the mean
ST: Staurosporine
SU: Sunitinib
TK: Tyrosine kinase
TKIs: Tyrosine kinase inhibitors
UHPLC-MS: Ultra-High Performance Liquid Chromatography-Mass Spectrometry
