# Supplementary material for: Identification of MBT3T as a new effective therapeutic option in imatinib-resistant gastrointestinal stromal tumors (GISTs)
Source: J Exp Clin Cancer Res. 2026 Mar 21;45:108. doi: 10.1186/s13046-026-03698-3 (PMC13130600; doi:10.1186/s13046-026-03698-3)
Supplement: Supplementary file 1 — Supplementary Material 1. [file 13046_2026_3698_MOESM1_ESM.docx]

**Supplementary Data for:**

**Identification of MBT3T as a new effective therapeutic option in imatinib-resistant gastrointestinal stromal tumors (GISTs)**

**Authors:**

Gloria Ravegnini ^1,2,#,*^, Daniele Esposito ^1,#^, Francesca Gorini ^1,#^, Tainah Dorina Marforio^3^

, Emma Coschina ^1^, Eva Benuzzi ^1^, Antonella Simone ^1^, Ahmed Samaha ^4^ , Aldo Di Vito ^1^, Martina Rossi ^1^ , Patrizia Hrelia ^1,2^, Alessandra Locatelli ^1^, Matteo Calvaresi^3,5^, Nicola Facchinello ^1,4^, Rita Morigi ^1,*^, Sabrina Angelini ^1,2^

**Affiliations:**

^1 Department of Pharmacy and Biotechnology, University of Bologna, Bologna, 40126, Italy^

^2 Clinical Pharmacology Unit, IRCCS Azienda Ospedaliero-Universitaria di Bologna, Bologna, 40138, Italy^

^3 Department of Chemistry "Giacomo Ciamician", Alma Mater Studiorum - University of Bologna, Bologna, 40126, Italy^

^4 Neuroscience Institute, Italian Research Council (CNR), 35131 Padua, Italy^

^5 IRCCS Azienda Ospedaliero – Universitaria di Bologna, Preclinical & Translational Research in Oncology lab (PRO), Bologna 40138, Italy^

**Table of contents:**

Figure S1 p.S3

Figure S2 p.S4

Synthesis of the benzo[d]imidazo[2,1-b]thiazole derivatives p.S5-S6

Figure S3 p.S6

Figure S4 p.S6

Figure S5 p.S7

Figure S6 p.S7

Figure S7 p.S8

Figure S8 p.S8

Figure S9 p.S9

Figure S10 p.S9

Synthetic procedures, compounds data p.S10-S17

^1^H NMR and ^13^C NMR spectra of the new compounds p.S18-43

^1^H-^13^C HSQC NMR spectrum of compound 5 p.S22

^1^H-^13^C HSQC NMR spectrum of compound 15 p.S28

1D-NOESY spectra of compound 17 p.S31

1D-NOESY spectrum of compound 24 p.S38

UHPLC-MS traces of compounds 1-27 p.S44-S57

References p.S58

|  | | | | |
| --- | --- | --- | --- | --- |
| **Compound** | **Ar** | **R_1_** | **R_2_** | **R_3_** |
| 1 |  | H | H | H |
| 2 |  | OCH_3_ | H | H |
| 3 (MBT3T) |  | H | H | OCH_3_ |
| 4 |  | H | H | Cl |
| 5 |  | H | H | F |
| 6 |  | H | CH_3_ | CH_3_ |
| 7 |  | H | H | H |
| 8 |  | H | H | H |
| 9 |  | H | H | H |
| 10 |  | H | H | OCH_3_ |
| 11 |  | H | H | OCH_3_ |
| 12 |  | H | H | H |
| 13 |  | H | H | H |
| 14 |  | H | H | OCH_3_ |
| 15 |  | H | H | F |

**Figure S1.** Benzo[d]imidazo[2,1-b]thiazole derivatives.

|  | | | | | | |
| --- | --- | --- | --- | --- | --- | --- |
| **Compound** | **R** | **R_1_** | **R_2_** | **R_3_** | **R_4_** | **R_5_** |
| 16 | CH_3_ | H | H | H | H | H |
| 17 | CH_3_ | H | H | Cl | H | H |
| 18 | CH_3_ | H | H | OCH_3_ | H | H |
| 19 | CH_3_ | H | H | OCH_3_ | CH_3_ | H |
| 20 | CH_3_ | H | OCH_3_ | H | H | OCH_3_ |
| 21 |  | H | H | H | H | H |
| 22 |  | H | H | Cl | H | H |
| 23 |  | OCH_3_ | H | OH | H | H |
| 24 |  | OCH_3_ | H | OCH_3_ | H | H |
| 25 |  | OCH_3_ | H | F | H | H |
| 26 |  | OCH_3_ | H | Cl | H | H |
| 27 |  | OCH_3_ | H | Br | H | H |

**Figure S2.** Benzo[d]imidazo[2,1-b]thiazolylmethyleneindolinone derivatives.

**Synthesis of the benzo[d]imidazo[2,1-b]thiazole derivatives**

The benzo[d]imidazo[2,1-b]thiazoles 1-15 were synthesized by reacting the appropriate 2-aminobenzothiazole (28-33) with the 2-bromo-1-arylethan-1-ones 34-40 to obtain the intermediates 41-47 which were in turn submitted to cyclization in HCl 2N (Figure S3). In a previous work, we described the synthesis of compound 8 [1], where it was used as a starting compound. Moreover, research performed using the Reaxys database highlighted that the synthesis of the derivatives 1, 13 [2] and 9-11 [3], had already been described, although using a different synthetic method. However, none of these compounds had been previously studied as antiproliferative agents. Finally, compound 7 is mentioned in a patent [4] which does not report any data of the compound; therefore, its synthesis and spectroscopic data have been described in the Synthetic procedures section (p.S10). The structures of compounds 1-15 were confirmed by means of ^1^H NMR and ^13^C NMR spectroscopy and mass spectrometry. ^13^C NMR spectra of compounds containing a fluorine atom (5 and 15) showed doublets because of the C - F coupling. To better interpret the ^13^C spectra of these compounds and calculate the C - F coupling constants (reported in the transcripts), HSQC experiments were performed.

The aldehydes 49-51 were obtained by performing a Vilsmeier-Haack reaction (Figure S4) on compounds 1, 3, 48 [5] and were obtained in good yields. The aldehyde 49 is cited in a patent [6] as a starting compound, but its synthesis and spectroscopic data are not reported, therefore we described it. The 2-indolinones 52 and 53 are commercially available, whereas the oxindoles 54-59 were prepared according to previously reported procedures [7–12].

The Knoevenagel reaction between the aldehydes 49-51 and the oxindoles 52-59 was performed in methanol/piperidine to achieve compounds 16-27 (Figure S4) in good yields. Most derivatives were obtained as pure stereoisomers.

The geometrical configuration was studied by performing Nuclear Overhauser Effect (NOE) experiments on compounds 17 and 24 to determine whether the methine bridge and the proton at the 4 position of the indole (ind-4) are close in space (*Z* configuration) or not (*E* configuration, see Figure S5). As far as compound 17 is concerned, the irradiation of the methine bridge proton –CH= at 7.90 ppm produced NOE at 7.32 ppm, the doublet due to the proton at position 5 of the benzo[d]imidazo[2,1-b]thiazole core, but not at the ind-4 proton. The irradiation of the doublet at 7.32 ppm gave NOE at 7.52 ppm (proton at position 6) and confirmed the closeness of the methine bridge (NOE at 7.90 ppm). These results are in agreement with the *E* configuration (Figure S5).

Analogues NOE experiments were performed on compound 24. When the methine bridge proton was irradiated (7.74 ppm), NOE was observed at 7.93 ppm (proton at position 5 of the benzo[d]imidazo[2,1-b]thiazole), but not at ind-4. This confirmed the E configuration also for this compound (Figure S5).

On the basis of these results and by comparing the ^1^H NMR spectra, we concluded that the Knoevenagel adducts 16-27 were obtained as pure *E* isomers, with the only exceptions of compounds 20, 26 and 27 which gave *E/Z* mixtures, even if with a predominance of isomer *E*. In fact, the *E/Z* ratio, determined on account of the ^1^H NMR spectra, corresponds to 78/22, 86/14 and 88/12 respectively. Compounds 20, 26 and 27 in solution undergo progressive isomerization. This behaviour, together with the long acquisition time required due to the presence of several quaternary carbons, led to crowded ^13^C NMR spectra, making the description inaccurate and meaningless.

**Figure S3.** Synthesis of compounds 1-15.

**Figure S4**. Synthesis of compounds 16-27.

**Figure S5.** *E* and *Z* isomers of compounds 16-27 and NOE connections detected in compounds 17 and 24.

**
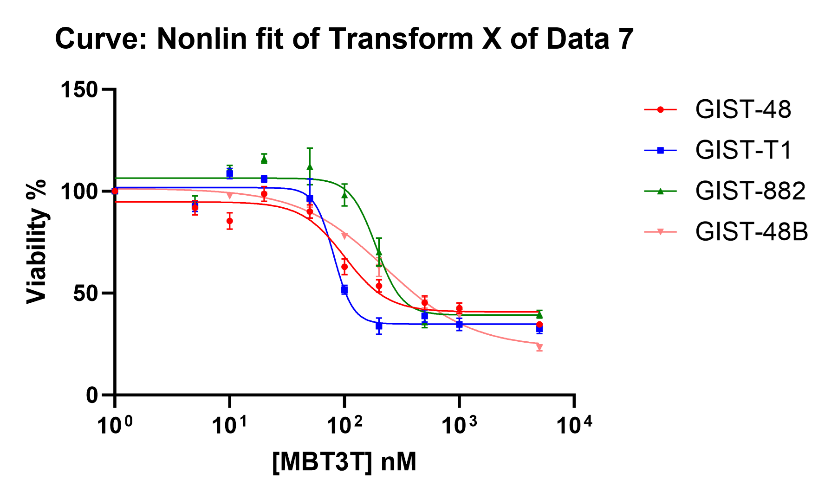
**

**Figure S6.** Viability after 72 h-treatment with MBT3T, assessed through Cell Titer Glo (CTG, Promega) assay in GIST cell lines.


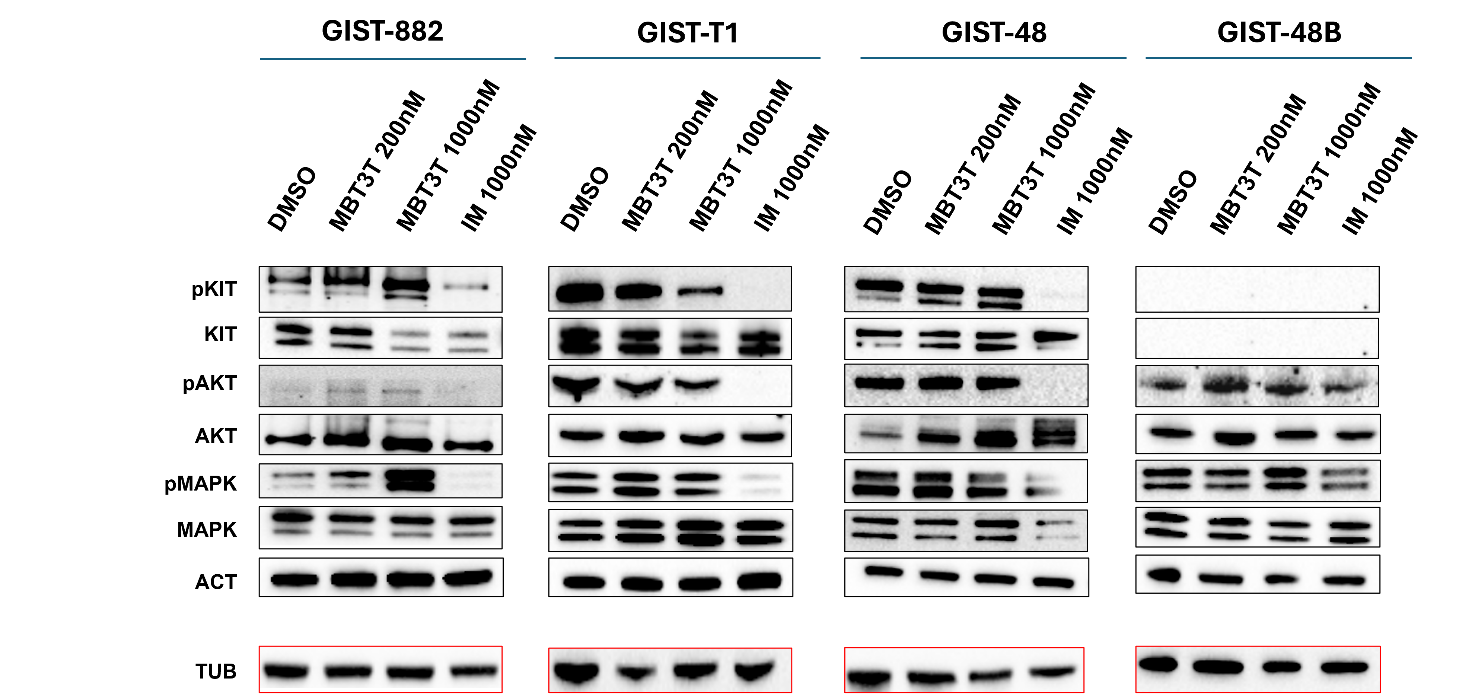


**Figure S7.** Immunoblotting evaluation of the KIT downstream cascade (KIT, phosphoKIT, AKT, phosphoAKT, MAPK, phosphoMAPK).


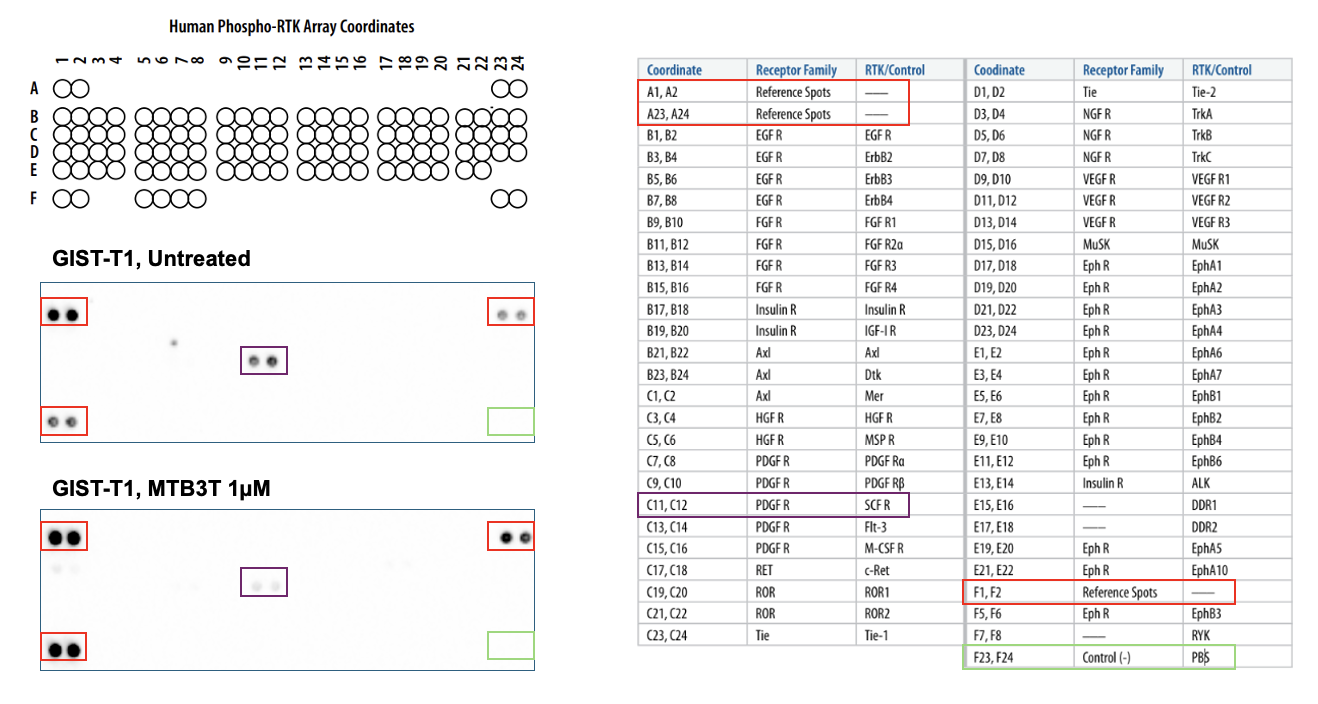


**Figure S8.** Human Phospo-RTK array in GIST-T1 cell line treated and untreated with MTB3T**.**

| **CHEMBL ID** | **SIMILARITY** | **SMILES** |
| --- | --- | --- |
| CHEMBL1497019 | 0.992 | COC1=CC=C2N3C=C(N=C3SC2=C1)C1=CC=CC=C1 |
| CHEMBL130475 | 0.990 | COC1=CC=C(C=C1)C1=CN2C(SC3=CC=CC=C23)=N1 |
| CHEMBL1908959 | 0.988 | COC1=CC=C2N3C=C(N=C3SC2=C1)C1=CC=C(I)C=C1 |
| CHEMBL1771169 | 0.988 | COC1=CC=C2N3C=C(N=C3SC2=C1)C1=CC=C([123I])C=C1 |
| CHEMBL1771173 | 0.987 | COC1=CC=C2N3C=C(N=C3SC2=C1)C1=CC=C(Br)C=C1 |
| CHEMBL362918 | 0.985 | COC1=CC=C2N3C=C(N=C3SC2=C1)C1=CC=C(C)C=C1 |
| CHEMBL129214 | 0.983 | COC1=CC=CC(=C1)C1=CN2C(SC3=CC=CC=C23)=N1 |
| CHEMBL1972678 | 0.983 | COC1=CC=C(C=C1)C1=CN2C(SC3=CC(C)=CC=C23)=N1 |
| CHEMBL129503 | 0.979 | OC1=CC=CC(=C1)C1=CN2C(SC3=CC=CC=C23)=N1 |
| CHEMBL125410 | 0.975 | CC1=C(O)C=C(C=C1)C1=CN2C(SC3=CC=CC=C23)=N1 |
| CHEMBL1970473 | 0.974 | COC1=CC(C2=CN3C(SC4=CC=CC=C34)=N2)=C(OC)C=C1 |
| CHEMBL339325 | 0.974 | COC1=C(O)C=C(C=C1)C1=CN2C(SC3=CC=CC=C23)=N1 |
| CHEMBL340364 | 0.970 | OC1=C(Cl)C=CC(=C1)C1=CN2C(SC3=CC=CC=C23)=N1 |
| CHEMBL129245 | 0.967 | CC1=C(N=C2SC3=CC=CC=C3N12)C1=CC(O)=CC=C1 |
| CHEMBL128953 | 0.967 | CC1=CC=C2N3C=C(N=C3SC2=C1)C1=CC(O)=CC=C1 |
| CHEMBL126901 | 0.963 | OC1=C(C=CC=C1)C1=CN2C(SC3=CC=CC=C23)=N1 |
| CHEMBL129484 | 0.963 | NC1=CC=CC(=C1)C1=CN2C(SC3=CC=CC=C23)=N1 |
| CHEMBL434046 | 0.961 | O=CNC1=CC=CC(=C1)C1=CN2C(SC3=CC=CC=C23)=N1 |
| CHEMBL126734 | 0.960 | [O-][N+](=O)C1=CC=CC(=C1)C1=CN2C(SC3=CC=CC=C23)=N1 |

**Figure S9.** CHEMBL identifier (ID), similarity score and SMILES of structures obtained by SwissSimilarity tool. On the right, the 2D structure of CHMBL1497019 and MBT3T.


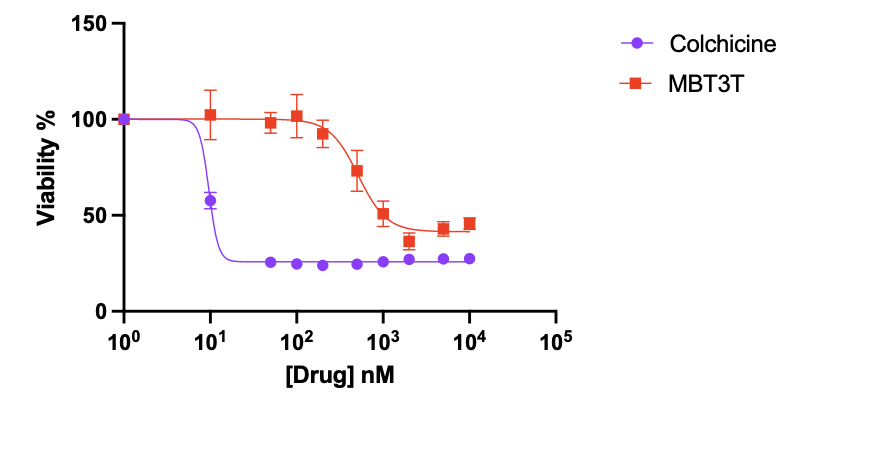


**Figure S10.** Viability after 72 h-treatment with MBT3T, and colchicine in WS1 cell line (fibroblasts). IC_50_ MBT3T: 516nM and IC_50_ Colchicine: 9.7nM.

**Synthetic procedures**

The 2-aminobenzothiazoles 28-33, 2-bromoacetophenone (39) and the oxindoles 52, 53 are commercially available. Bromoacetyl derivatives 34-38, 40 [13–16], 2-methylbenzo[d]imidazo[2,1-b]thiazole 48 [5] and oxindoles 54-59 [7–12] were prepared according to literature procedures.

For ^1^H NMR and ^13^C NMR, the chemical shift was expressed in δ (ppm) and referenced to the residual peak of the solvent as the internal standard (DMSO-d_6_: δ H = 2.50 ppm; δ C = 39.52 ppm). The coupling constant values (*J*) were determined in Hertz (abbreviation: bz=benzene, im=imidazole, th=thienyl, ph=phenyl, thia=thiazolyl; py=pyridyl, ind=indole; CH=methine bridge).

**General procedure for the synthesis of compounds 1-15 (Figure S3).**

The appropriate 2-aminobenzothiazole 28-33 (20 mmol) was dissolved in 100 mL of acetone and treated with the appropriate 2-bromoacetylderivative 34-40 (1 eq.). The reaction mixture was refluxed for 1-5 h (according to a TLC test). After cooling, the solid product was separated by filtration, and the resulting intermediate salt was used in the subsequent step without further purification. It was refluxed for 1-4 h with 200 mL of 2N HCl and, before complete cooling, the solution was cautiously basified by dropwise addition of 15% NH_4_OH. The solid product obtained was collected by filtration and crystallized from ethanol.

Compounds 1, 13 [2] and 9-11 [3] had been previously described.

*5-Methoxy-2-(thiophen-3-yl)benzo[d]imidazo[2,1-b]thiazole (2)*

Beige powder. ^1^H NMR (400 MHz, DMSO-d_6_) δ 8.42 (s, 1H, im), 7.81 (t, *J* = 2.2 Hz, 1H, th), 7.60 (d, *J* = 2.2 Hz, 2H, th), 7.57 (dd, *J* = 8.2, 0.9 Hz, 1H, bz), 7.37 (t, *J* = 8.2 Hz, 1H, bz), 7.21 (dd, *J* = 8.2, 0.9 Hz, 1H, bz), 4.07 (s, 3H, OCH_3_). ^13^C NMR (100 MHz, DMSO-*d*_6_) δ 147.28, 146.06, 142.56, 135.85, 130.01, 126.61, 125.72, 125.65, 121.54, 119.65, 116.47, 111.29, 109.11, 56.37. MW = 286.37. Yield = 76%. MS (ESI): *m/z* calcd. for C_14_H_10_N_2_OS_2_ [M+ H]⁺: 287.03; found: 287.01. Anal. Calcd for C_14_H_10_N_2_OS_2_: C, 58.72; H, 3.52; N, 9.78. Found: C, 58.68; H, 3.53; N, 9.80.

*7-Methoxy-2-(thiophen-3-yl)benzo[d]imidazo[2,1-b]thiazole (3)*

White powder. ^1^H NMR (600 MHz, DMSO-*d*_6_) δ 8.52 (s, 1H, im), 7.86 (d, *J* = 8.8 Hz, 1H, bz), 7.73 (dd, *J* = 3.0, 1.2 Hz, 1H, th), 7.67 (d, *J* = 2.5 Hz, 1H, bz), 7.61 (dd, *J* = 5.0, 3.0 Hz, 1H, th), 7.48 (dd, *J* = 5.0, 1.2 Hz, 1H, th), 7.14 (dd, *J* = 8.8, 2.5 Hz, 1H, bz), 3.83 (s, 3H, OCH_3_). ^13^C NMR (150 MHz, DMSO-*d*_6_) δ 156.86, 146.09, 142.46, 135.83, 130.43, 126.83, 125.95, 125.40, 119.52, 113.79, 113.77, 109.46, 108.74, 55.83. MW = 286.37. Yield = 85%. MS (ESI): *m/z* calcd. for C_14_H_10_N_2_OS_2_ [M+H]⁺: 287.03; found: 287.01. Anal. Calcd for C_14_H_10_N_2_OS_2_: C, 58.72; H, 3.52; N, 9.78. Found: C, 58.75; H, 3.53; N, 9.81.

*7-Chloro-2-(thiophen-3-yl)benzo[d]imidazo[2,1-b]thiazole (4)*

White powder. ^1^H NMR (400 MHz, DMSO-*d*_6_) δ 8.60 (s, 1H, im), 8.21 (d, *J* = 2.1 Hz, 1H, bz), 7.96 (d, *J* = 8.6 Hz, 1H, bz), 7.76 (dd, *J* = 3.0, 1.3 Hz, 1H, th), 7.65 – 7.60 (m, 2H, bz+th), 7.48 (dd, *J* = 5.0, 1.3 Hz, 1H, th). ^13^C NMR (100 MHz, DMSO-*d*_6_) δ 146.87, 143.13, 135.59, 130.99, 130.80, 128.87, 126.99, 126.79, 125.39, 124.65, 120.04, 114.39, 109.03. MW = 290.78. Yield = 82%. MS (ESI): *m/z* calcd. for C_13_H_7_ClN_2_S_2_ [M+H]⁺: 290.98; found: 291.01. Anal. Calcd for C_13_H_7_ClN_2_S_2_: C, 53.70; H, 2.43; N, 9.63. Found: C, 53.72; H, 2.44; N, 9.65.

*7-Fluoro-2-(thiophen-3-yl)benzo[d]imidazo[2,1-b]thiazole (5)*

Beige powder. ^1^H NMR (400 MHz, DMSO-*d*_6_) δ 8.58 (s, 1H, im), 8.01 (dd, *J* = 8.8, 2.5 Hz, 1H, bz-8), 7.97 (dd, *J* = 8.8, 4.4 Hz, 1H, bz-5), 7.75 (dd, *J* = 3.0, 1.2 Hz, 1H, th-2), 7.62 (dd, *J* = 5.0, 3.0 Hz, 1H, th-5), 7.48 (dd, *J* = 5.0, 1.2 Hz, 1H, th-4), 7.44 (td, *J* = 8.8, 2.5 Hz, 1H, bz-6). ^13^C NMR (100 MHz, DMSO-*d*_6_) δ 159.03 (d, *^1^J_C-F_* = 241.5 Hz, C-F), 146.67, 142.93, 135.69, 130.72 (d, *^3^J_C-F_* = 11.1 Hz), 128.72 (d, *^4^J_C-F_* = 2.0 Hz), 126.93, 125.38, 119.85, 114.25 (d, *^3^J_C-F_* = 9.4 Hz), 114.17 (d, *^2^J_C-F_* = 24.3 Hz), 112.11 (d, *^2^J_C-F_* = 28.2 Hz), 108.99. 109.03. MW = 274.33. Yield = 73%. MS (ESI): *m/z* calcd. for C_13_H_7_FN_2_S_2_ [M+H]⁺: 275.01; found: 275.01. Anal. Calcd for C_13_H_7_FN_2_S_2_: C, 56.92; H, 2.57; N, 10.21. Found: C, 56.95; H, 2.58; N, 10.24.

*6,7-Dimethyl-2-(thiophen-3-yl)benzo[d]imidazo[2,1-b]thiazole (6)*

Beige powder. ^1^H NMR (400 MHz, DMSO-*d*_6_) δ 8.49 (s, 1H, im), 7.75 – 7.71 (m, 3H, bz+th), 7.61 (dd, *J* = 5.0, 3.0 Hz, 1H, th), 7.48 (dd, *J* = 5.0, 1.3 Hz, 1H, th), 2.35 (s, 3H, CH_3_), 2.31 (s, 3H, CH_3_). ^13^C NMR (100 MHz, DMSO-*d*_6_) δ 146.65, 142.72, 135.87, 135.43, 133.79, 130.01, 126.83, 125.96, 125.43, 124.87, 119.62, 113.75, 108.55, 19.69, 19.43. MW = 284.40. Yield = 74%. MS (ESI): *m/z* calcd. for C_15_H_12_N_2_S_2_ [M+H]⁺: 285.05; found: 285.01. Anal. Calcd for C_15_H_12_N_2_S_2_: C, 63.35; H, 4.25; N, 9.85. Found: C, 63.37; H, 4.26; N, 9.87.

*2-(2,5-Dimethylthiophen-3-yl)benzo[d]imidazo[2,1-b]thiazole (7)*

Beige powder. ^1^H NMR (600 MHz, DMSO-*d*_6_) δ 8.45 (s, 1H, im), 8.03 (dd, *J* = 7.6, 1.2 Hz, 1H, bz), 8.02 (dd, *J* = 7.6, 1.2 Hz, 1H, bz), 7.56 (td, *J* = 7.6, 1.2 Hz, 1H, bz), 7.42 (td, *J* = 7.6, 1.2 Hz, 1H, bz), 7.08 (s, 1H, th), 2.59 (s, 3H, CH_3_), 2.40 (s, 3H, CH_3_). ^13^C NMR (150 MHz, DMSO-*d*_6_) δ 146.03, 143.06, 134.67, 131.85, 130.80, 130.60, 129.08, 126.61, 125.96, 125.01, 124.94, 113.40, 109.55, 14.79, 14.67. MW = 284.40. Yield = 70%. MS (ESI): *m/z* calcd. for C_15_H_12_N_2_S_2_ [M+H]⁺: 285.05; found: 285.11. Anal. Calcd for C_15_H_12_N_2_S_2_: C, 63.35; H, 4.25; N, 9.85. Found: C, 63.37; H, 4.26; N, 9.87.

*2-(Thiazol-2-yl)benzo[d]imidazo[2,1-b]thiazole (12)*

Beige powder. ^1^H NMR (400 MHz, DMSO-*d*_6_) δ 8.93 (s, 1H, im), 8.16 (dd, *J* = 8.1, 1.2 Hz, 1H, bz), 8.06 (dd, *J* = 8.1, 1.2 Hz, 1H, bz), 7.89 (d, *J* = 3.2 Hz, 1H, thia), 7.70 (d, *J* = 3.2 Hz, 1H, thia), 7.58 (td, *J* = 7.7, 1.2 Hz, 1H, bz), 7.46 (td, *J* = 7.7, 1.2 Hz, 1H, bz). ^13^C NMR (100 MHz, DMSO-*d*_6_) δ 162.38, 147.33, 143.56, 141.17, 131.57, 129.44, 126.77, 125.70, 125.07, 119.39, 114.02, 110.58. MW = 257.33. Yield = 64%. MS (ESI): *m/z* calcd. for C_12_H_7_N_3_S_2_ [M+H]⁺: 258.02; found: 258.00. Anal. Calcd for C_12_H_7_N_3_S_2_: C, 56.01; H, 2.74; N, 16.33. Found: C, 56.03; H, 2.74; N, 16.35.

*7-Methoxy-2-(pyridin-2-yl)benzo[d]imidazo[2,1-b]thiazole (14)*

Light pink powder. ^1^H NMR (400 MHz, DMSO-*d*_6_) δ 8.82 (s, 1H, im), 8.56 (ddd, *J* = 4.8, 1.8, 1.1 Hz, 1H, py), 8.04 (d, *J* = 8.9 Hz, 1H, bz), 7.95 (dt, *J* = 7.8, 1.1 Hz, 1H, py), 7.84 (td, *J* = 7.8, 1.8 Hz, 1H, py), 7.67 (d, *J* = 2.5 Hz, 1H, bz), 7.27 (ddd, *J* = 7.8, 4.8, 1.1 Hz, 1H, py), 7.13 (dd, *J* = 8.9, 2.5 Hz, 1H, bz), 3.83 (s, 3H, OCH_3_). ^13^C NMR (100 MHz, DMSO-*d*_6_) δ 157.11, 152.57, 149.30, 146.41, 146.30, 136.94, 130.63, 125.88, 122.15, 118.87, 114.44, 113.84, 111.55, 109.30, 55.84. MW = 281.33. Yield = 85%. MS (ESI): *m/z* calcd. for C_15_H_11_N_3_OS [M+H]⁺: 282.07; found: 282.01. Anal. Calcd for C_15_H_11_N_3_OS: C, 64.04; H, 3.94; N, 14.94. Found: C, 64.07; H, 3.95; N, 14.96.

*7-Fluoro-2-(pyridin-2-yl)benzo[d]imidazo[2,1-b]thiazole (15)*

Beige powder. ^1^H NMR (400 MHz, DMSO-*d*_6_) δ 8.89 (s, 1H, im), 8.57 (ddd, *J* = 4.8, 1.8, 1.1 Hz, 1H, py), 8.18 (dd, *J* = 8.9, 4.6 Hz, 1H, bz-5), 8.02 (dd, *J* = 8.9, 2.6 Hz, 1H, bz-8), 7.95 (dt, *J* = 7.6, 1.1 Hz, 1H, py), 7.85 (td, *J* = 7.6, 1.8 Hz, 1H, py), 7.45 (td, *J* = 9.0, 2.6 Hz, 1H, bz-6), 7.28 (ddd, *J* = 7.6, 4.8, 1.1 Hz, 1H, py). ^13^C NMR (100 MHz, DMSO-*d*_6_) δ 159.26 (d, *^1^J_C-F_* = 241.9 Hz), 152.36, 149.36, 146.94, 146.67, 137.01, 130.94 (d, *^3^J_C-F_* = 11.1 Hz), 128.67 (d, *^4^J*_C-F_ = 1.9 Hz), 122.34, 118.98, 114.95 (d, *^3^J_C-F_* = 9.2 Hz), 114.22 (d, *^2^J_C-F_* = 24.8 Hz), 112.07 (d, *^2^J_C-F_* = 28.2 Hz), 111.87. MW = 269.30. Yield = 86%. MS (ESI): *m/z* calcd. for C_14_H_8_FN_3_S [M+H]⁺: 270.05; found: 270.10. Anal. Calcd for C_14_H_8_FN_3_S: C, 62.44; H, 2.99; N, 15.60. Found: C, 62.48; H, 3.00; N, 15.63.

**General procedure for the synthesis of compounds 16-27 (Figure S4).**

The appropriate oxindole 52-59 (10 mmol) was dissolved in methanol (100 mL) and treated with the properly substituted aldehyde 49-51 (1 eq.) and piperidine (1 mL). The reaction mixture was refluxed for 5-10 h according to a TLC test. The precipitate, formed on cooling, was collected by filtration and crystallized from methanol (compounds 16-22) or toluene (compounds 23-27).

*(E)-3-((2-methylbenzo[d]imidazo[2,1-b]thiazol-3-yl)methylene)indolin-2-one (16)*

Yellow powder. ^1^H NMR (600 MHz, DMSO-*d*_6_) δ 10.70 (s, 1H, NH), 8.08 (d, *J* = 7.8, 1H, bz), 7.82 (s, 1H, CH), 7.72 (d, *J* = 7.8, 1H, bz), 7.53 (t, *J* = 7.8, 1H, bz), 7.43 (t, *J* = 7.8, 1H, bz), 7.23 (t, *J* = 7.9 Hz, 1H, ind), 7.02 (d, *J* = 7.9 Hz, 1H, ind), 6.92 – 6.87 (m, 2H, ind), 2.16 (s, 3H, CH_3_). ^13^C NMR (150 MHz, DMSO-*d*_6_) δ 168.19, 148.93, 145.61, 142.83, 132.53, 129.97, 129.17, 127.81, 126.98, 125.17, 124.99, 123.31, 121.38, 121.06, 120.56, 119.55, 113.35, 109.90, 15.60. MW = 331.39. Yield = 48%. MS (ESI): *m/z* calcd. for C_19_H_13_N_3_OS [M+H]⁺: 332.09; found: 332.12. Anal. Calcd for C_19_H_13_N_3_OS_:_ C, 68.86; H, 3.95; N, 12.68. Found: C, 68.88; H, 3.95; N, 12.70.

*(E)-5-chloro-3-((2-methylbenzo[d]imidazo[2,1-b]thiazol-3-yl)methylene)indolin-2-one (17)*

Dark orange powder. ^1^H NMR (600 MHz, DMSO-*d*_6_) δ 10.84 (s, 1H, NH), 8.09 (d, *J* = 7.9 Hz, 1H, bz), 7.92 (s, 1H, CH), 7.73 (d, *J* = 7.9 Hz, 1H, bz), 7.54 (t, *J* = 7.9 Hz, 1H, bz), 7.44 (t, *J* = 7.9 Hz, 1H, bz), 7.29 (dd, *J* = 8.2, 2.4 Hz, 1H, ind-6), 6.96 (d, *J* = 2.4 Hz, 1H, ind-4), 6.92 (d, *J* = 8.2 Hz, 1H, ind-7), 2.15 (s, 3H, CH_3_). ^13^C NMR (150 MHz, DMSO-*d*_6_) δ 167.97, 149.66, 145.98, 141.54, 132.55, 129.33, 129.22, 127.01, 126.30, 125.23, 125.18, 125.07, 122.81, 122.73, 121.37, 120.62, 113.52, 111.26, 15.76. MW = 365.84. Yield = 40%. MS (ESI): *m/z* calcd. for C_19_H_12_ClN_3_OS [M+H]⁺: 366.05; found: 366.23. Anal. Calcd for C_19_H_12_ClN_3_OS_:_ C, 62.38; H, 3.31; N, 11.49. Found: C, 62.39; H, 3.30; N, 11.51.

*(E)-5-methoxy-3-((2-methylbenzo[d]imidazo[2,1-b]thiazol-3-yl)methylene)indolin-2-one (18)*

Red powder. ^1^H NMR (600 MHz, DMSO-*d*_6_) δ 10.51 (s, 1H, NH), 8.08 (d, *J* = 8.2 Hz, 1H, bz), 7.81 (s, 1H, CH), 7.70 (d, *J* = 8.2 Hz, 1H, bz), 7.52 (t, *J* = 8.2, 1H, bz), 7.42 (t, *J* = 8.2 Hz, 1H, bz), 6.84 (dd, *J* = 8.2, 2.4 Hz, 1H, ind-6), 6.81 (d, *J* = 8.2 Hz, 1H, ind-7), 6.57 (d, *J* = 2.4 Hz, 1H, ind-4), 3.55 (s, 3H, OCH_3_), 2.19 (s, 3H, CH_3_). ^13^C NMR (150 MHz, DMSO-*d*_6_) δ 168.22, 154.29, 149.05, 145.56, 136.57, 132.55, 129.19, 128.23, 126.94, 125.14, 125.00, 121.84, 120.43, 119.87, 115.39, 113.43, 110.30, 109.38, 55.28, 15.64. MW = 361.42. Yield = 35%. MS (ESI): *m/z* calcd. for C_20_H_15_N_3_O_2_S [M+H]⁺: 362.10; found: 362.23. Anal. Calcd for C_20_H_15_N_3_O_2_S_:_ C, 66.47; H, 4.18; N, 11.63. Found: C, 66.49; H, 4.18; N, 11.65.

*(E)-5-methoxy-6-methyl-3-((2-methylbenzo[d]imidazo[2,1-b]thiazol-3-yl)methylene)indolin-2-one (19)*

Red powder. ^1^H NMR (600 MHz, DMSO-*d*_6_) δ 10.45 (s, 1H, NH), 8.07 (d, *J* = 8.2 Hz, 1H, bz), 7.72 (s, 1H, CH), 7.69 (d, *J =* 8.2 1H, bz), 7.50 (t, *J* = 8.2 Hz, 1H, bz), 7.42 (t, *J* = 8.2 Hz, 1H, bz), 6.70 (s, 1H, ind), 6.54 (s, 1H, ind), 3.47 (s, 3H, OCH_3_), 2.22 (s, 3H, CH_3_), 2.13 (s, 3H, CH_3_). ^13^C NMR (150 MHz, DMSO-*d*_6_) δ 168.52, 152.14, 148.82, 145.37, 136.56, 132.59, 129.17, 128.57, 128.42, 126.90, 125.10, 124.97, 120.49, 118.94, 118.31, 113.46, 112.07, 106.13, 55.20, 16.56, 15.63. MW = 375.45. Yield = 30%. MS (ESI): *m/z* calcd. for C_21_H_17_N_3_O_2_S [M+H]⁺: 376.11; found: 376.24. Anal. Calcd for C_21_H_17_N_3_O_2_S: C, 67.18; H, 4.56; N, 11.19. Found: C, 67.21; H, 4.57; N, 11.21.

*(E)-4,7-dimethoxy-3-((2-methylbenzo[d]imidazo[2,1-b]thiazol-3-yl)methylene)indolin-2-one (20)*

Brown powder. ^1^H NMR (600 MHz, DMSO-*d*_6_) δ 10.61 (s, 1H, NH), 8.18 (s, 1H, CH), 8.06 (d, *J* = 8.0 Hz, 1H, bz), 7.71 (d, J = 8.0, 1H, bz), 7.52 (t, *J* = 8.0 Hz, 1H, bz), 7.41 (t, *J* = 8.0 Hz, 1H, bz), 6.95 (d, *J* = 8.8 Hz, 1H, ind), 6.64 (d, *J* = 8.8 Hz, 1H, ind), 3.91 (s, 3H, OCH_3_), 3.79 (s, 3H, OCH_3_), 2.21 (s, 3H, OCH_3_). MW = 391.45. Yield = 30%. MS (ESI): *m/z* calcd. for C_21_H_17_N_3_O_3_S [M+H]⁺: 392.11; found: 392.14. Anal. Calcd for C_21_H_17_N_3_O_3_S: C, 64.44; H, 4.38; N, 10.73. Found: C, 64.47; H, 4.39; N, 10.75.

*(E)-3-((2-(thiophen-3-yl)benzo[d]imidazo[2,1-b]thiazol-3-yl)methylene)indolin-2-one (21)*

Yellow powder. ^1^H NMR (600 MHz, DMSO-*d*_6_) δ 10.72 (s, 1H, NH), 8.08 (d, *J* = 8.2, 1H, bz), 7.93 (s, 1H, CH), 7.69 (dd, *J* = 3.0, 1.3 Hz, 1H, th), 7.64 (d, 8.2, 1H, bz), 7.52 (dd, *J* = 5.0, 3.0 Hz, 1H, th), 7.45 (t, *J* = 8.2 Hz, 1H, bz), 7.40 (m, 2H, bz+th), 7.11 (t, *J* = 7.7 Hz, 1H, ind), 6.82 (d, *J* = 7.7 Hz, 1H, ind), 6.60 (d, *J* = 7.7 Hz, 1H, ind), 6.56 (t, *J* = 7.7 Hz, 1H, ind ). ^13^C NMR (150 MHz, DMSO-*d*_6_) δ 167.95, 149.09, 142.98, 142.51, 134.85, 132.50, 130.65, 130.46, 129.40, 126.83, 126.69, 126.13, 125.18, 125.15, 123.57, 123.05, 121.14, 120.55, 119.52, 118.50, 113.64, 109.82. MW = 399.49. Yield = 52%. MS (ESI): *m/z* calcd. for C_22_H_13_N_3_OS_2_ [M+H]⁺: 400.06; found: 400.14. Anal. Calcd for C_22_H_13_N_3_OS_2:_ C, 66.15; H, 3.28; N, 10.52. Found: C, 66.17; H, 3.27; N, 10.55.

*(E)-5-chloro-3-((2-(thiophen-3-yl)benzo[d]imidazo[2,1-b]thiazol-3-yl)methylene)indolin-2-one (22)*

Orange powder. ^1^H NMR (600 MHz, DMSO-*d*_6_) δ 10.82 (s, 1H, NH), 8.11 (d, *J* = 8.0, 1H, bz), 8.06 (s, 1H, CH), 7.72 (m, 2H, th+bz), 7.53 (t, *J* = 8.0, 1H, bz), 7.50 (dd, *J* = 5.2, 3.0 Hz, 1H, th), 7.45 (t, *J* = 8.0, 1H, bz), 7.35 (dd, *J* = 5.2, 1.3 Hz, 1H, th), 7.14 (dd, *J* = 8.3, 2.1 Hz, 1H, ind-6), 6.80 (d, *J* = 8.3 Hz, 1H, ind-7), 6.58 (d, *J* = 2.1 Hz, 1H, ind-4). ^13^C NMR (150 MHz, DMSO-*d*_6_) δ 167.92, 149.85, 143.27, 141.49, 134.73, 132.55, 129.60, 129.46, 128.05, 127.01, 126.89, 125.91, 125.29, 125.26, 124.98, 123.55, 123.46, 122.06, 120.91, 118.75, 113.74, 110.87. MW = 433.93. Yield 48%. MS (ESI): *m/z* calcd. for C_22_H_12_ClN_3_OS_2_ [M+H]⁺: 434.02; found: 434.06. Anal. Calcd for C_22_H_12_ClN_3_OS_2_: C, 60.90; H, 2.79; N, 9.68. Found: C, 60.88; H, 2.80; N, 9.70.

*(E)-5-hydroxy-3-((7-methoxy-2-(thiophen-3-yl)benzo[d]imidazo[2,1-b]thiazol-3-yl)methylene)indolin-2-one (23)*

Orange powder. ^1^H NMR (400 MHz, DMSO-*d*_6_) δ 10.43 (s, 1H, NH), 8.78 (s, 1H, OH), 7.79 (s, 1H, CH), 7.72 (d, *J* = 2.6 Hz, 1H, bz), 7.65 (dd, *J* = 3.0, 1.3 Hz, 1H, th), 7.55 (dd, *J* = 5.0, 3.0 Hz, 1H, th), 7.46 (d, *J* = 9.0 Hz, 1H, bz), 7.41 (dd, *J* = 5.0, 1.3 Hz, 1H, th), 7.01 (dd, *J* = 9.0, 2.6 Hz, 1H, bz), 6.63 (d, *J* = 8.4 Hz, 1H, ind-7), 6.53 (dd, *J* = 8.4, 2.4 Hz, 1H, ind-6), 6.10 (d, *J* = 2.4 Hz, 1H, ind-4), 3.79 (s, 3H, OCH_3_). ^13^C NMR (100 MHz, DMSO-*d*_6_) δ 168.28, 157.13, 152.44, 148.78, 142.23, 135.75, 135.43, 132.56, 131.34, 127.10, 126.93, 126.58, 122.98, 121.73, 119.76, 118.49, 117.49, 114.60, 114.37, 111.34, 110.71, 109.77, 56.19. MW = 445.51. Yield 42%. MS (ESI): *m/z* calcd. for C_23_H_15_N_3_O_3_S_2_ [M+H]⁺: 446.07; found: 446.17. Anal. Calcd for C_23_H_15_N_3_O_3_S_2_ (445.51): C, 62.01; H, 3.40; N, 9.43. Found: C, 61.98; H, 3.41; N, 9.45.

*(E)-5-methoxy-3-((7-methoxy-2-(thiophen-3-yl)benzo[d]imidazo[2,1-b]thiazol-3-yl)methylene)indolin-2-one (24)*

Red powder. ^1^H NMR (400 MHz, DMSO-*d*_6_) δ 10.52 (s, 1H, NH), 7.91 (s, 1H, CH), 7.73 (d, *J* = 2.6 Hz, 1H, bz), 7.68 (dd, *J* = 2.9, 1.3 Hz, 1H, th), 7.53 (m, 2H, bz+th), 7.40 (dd, *J* = 5.1, 1.3 Hz, 1H, th), 7.06 (dd, *J* = 9.0, 2.6 Hz, 1H, bz), 6.76 – 6.69 (m, 2H, ind), 6.18 (d, *J* = 2.2 Hz, 1H, ind-4), 3.80 (s, 3H, OCH_3_), 3.27 (s, 3H, OCH_3_). ^13^C NMR (100 MHz, DMSO-*d*_6_) δ 168.10, 156.78, 154.00, 148.64, 142.29, 136.68, 134.98, 130.88, 130.32, 126.69, 126.56, 126.13, 123.00, 121.15, 119.59, 118.41, 116.58, 114.31, 114.12, 110.25, 109.37, 109.29, 55.81, 54.94. MW = 459.54. Yield 45%. MS (ESI): *m/z* calcd. for C_24_H_17_N_3_O_3_S_2_ [M+H]⁺: 460.08; found: 460.17. Anal. Calcd for C_24_H_17_N_3_O_3_S_2_: C, 62.73; H, 3.73; N, 9.14. Found: C, 62.75; H, 3.74; N, 9.16.

*(E)-5-fluoro-3-((7-methoxy-2-(thiophen-3-yl)benzo[d]imidazo[2,1-b]thiazol-3-yl)methylene)indolin-2-one (25)*

Orange powder. ^1^H NMR (400 MHz, DMSO-*d*_6_) δ 10.72 (s, 1H, NH), 7.99 (s, 1H, CH), 7.74 (d, *J* = 2.6 Hz, 1H, bz), 7.68 (dd, *J* = 3.0, 1.3 Hz, 1H, th), 7.56 (d, *J* = 9.0 Hz, 1H, bz ), 7.51 (dd, *J* = 5.0, 3.0 Hz, 1H, th), 7.36 (dd, *J* = 5.0, 1.3 Hz, 1H, th), 7.08 (dd, *J* = 9.0, 2.6 Hz, 1H, bz), 6.96 (td, *J* = 8.8, 2.6 Hz 1H, ind-6), 6.79 (dd, *J* = 8.5, 4.5 Hz, 1H, ind-7), 6.35 (dd, *J* = 9.2, 2.6 Hz, 1H, ind-4), 3.81 (s, 3H, OCH_3_). ^13^C NMR (100 MHz, DMSO-*d*_6_) δ 168.12, 157.16 (d, *^1^J_C-F_* = 235.7 Hz, C-F), 156.79, 149.15, 142.76, 139.12 (d, *^4^J_C-F_* = 2.0 Hz), 134.85, 130.94, 128.83, 126.78, 126.53, 126.02, 123.18, 121.58 (d, *^3^J_C-F_* = 9.2 Hz), 120.81, 118.40, 116.43 (d, *^2^J_C-F_* = 23.7 Hz), 114.31, 114.18, 110.60 (d, *^2^J_C-F_* = 25.9 Hz), 110.28 (d, *^3^J_C-F_* = 8.4 Hz), 109.41, 55.80. MW = 447.50. Yield 45%. MS (ESI): *m/z* calcd. for C_23_H_14_FN_3_O_2_S_2_ [M+H]⁺: 448.06; found: 448.07. Anal. Calcd for C_23_H_14_FN_3_O_2_S_2_ : C, 61.73; H, 3.15; N, 9.39. Found: C, 61.75; H, 3.15; N, 9.41.

*(E)-5-chloro-3-((7-methoxy-2-(thiophen-3-yl)benzo[d]imidazo[2,1-b]thiazol-3-yl)methylene)indolin-2-one (26)*

Orange powder. ^1^H NMR (400 MHz, DMSO-*d*_6_) δ 10.84 (s, 1H, NH), 8.02 (s, 1H, CH), 7.77 (d, *J* = 2.5 Hz, 1H, bz), 7.69 (s, 1H, th), 7.59 (d, *J* = 9.0 Hz, 1H, bz), 7.50 (m, 1H, th), 7.33 (d, *J* = 5.0 Hz, 1H, th), 7.18 – 7.08 (m, 2H, bz+ind-7), 6.81 (d, *J* = 8.3 Hz, 1H, ind-7), 6.56 (s, 1H, ind-4), 3.82 (s, 3H, OCH_3_). MW = 463.95. Yield 47%. MS (ESI): *m/z* calcd. for C_23_H_14_ClN_3_O_2_S_2_ [M+H]⁺: 464.03; found: 464.06. Anal. Calcd for C_23_H_14_ClN_3_O_2_S_2_: C, 59.54; H, 3.04; N, 9.06. Found: C, 59.56; H, 3.05; N, 9.07.

*(E)-5-bromo-3-((7-methoxy-2-(thiophen-3-yl)benzo[d]imidazo[2,1-b]thiazol-3-yl)methylene)indolin-2-one (27)*

Orange powder. ^1^H NMR (400 MHz, DMSO-*d*_6_) δ 10.85 (s, 1H, NH), 8.02 (s, 1H, CH), 7.77 (d, *J* = 2.6 Hz, 1H, bz), 7.68 (s, 1H, th), 7.60 (d, *J* = 9.0 Hz, 1H, bz), 7.50 (m, 1H, th), 7.32 (d, *J* = 5.0 Hz, 1H, th), 7.26 (d, *J* = 8.3 Hz, 1H, ind-6), 7.12 (dd, *J* = 9.0, 2.6 Hz, 1H, bz), 6.76 (d, *J* = 8.3 Hz, 1H, ind-7), 6.69 (s, 1H, ind-4), 3.82 (s, 3H, OCH_3_). MW = 508.41. Yield 35%. MS (ESI): *m/z* calcd. for C_23_H_14_BrN_3_O_2_S_2_ [M+H]⁺: 507.98; found: 508.08. Anal. Calcd for C_23_H_14_BrN_3_O_2_S_2_: C, 54.34; H, 2.78; N, 8.27. Found: C, 54.37; H, 2.79; N, 8.29.

**General procedure for the synthesis of the new aldehydes 49-51 (Figures S4).**

The Vilsmeier reagent was prepared at 0-5°C by dropping POCl_3_ (54 mmol) into a stirred solution of DMF (65 mmol) in CHCl_3_ (5 mL). The appropriate benzoimidazothiazole 1, 3 or 48 (5 mmol) was suspended in CHCl_3_ (20 mL). The mixture thus obtained was dropped into the Vilsmeier reagent while maintaining stirring and cooling. The reaction mixture was kept for 3 h at room temperature and under reflux for 2 h (49, 50) or 5 h (51). Chloroform was removed under reduced pressure, and the resulting oil was poured onto ice. The crude precipitated aldehydes were collected by filtration and crystallized from ethanol (49, 51), or toluene (50).

*2-(Thiophen-3-yl)benzo[d]imidazo[2,1-b]thiazole-3-carbaldehyde (49)*

Yellow powder. ^1^H NMR (600 MHz, DMSO-*d*_6_) δ 9.97 (s, 1H, CHO), 9.04 (d, *J* = 8.3, 1H, bz), 8.19 (dd, *J* = 3.0, 1.3 Hz, 1H, th), 8.12 (d, *J* = 8.3, 1H, bz), 7.75 (dd, *J* = 5.0, 3.0 Hz, 1H, th), 7.66 – 7.59 (m, 2H, bz+th), 7.51 (t, *J* = 8.3, 1H, bz). ^13^C NMR (150 MHz, DMSO-*d*_6_) δ 177.49, 154.66, 154.36, 133.24, 133.19, 129.41, 128.20, 127.52, 127.36, 126.77, 125.96, 125.84, 124.86, 117.41. MW = 284.35. Yield 92%. MS (ESI): *m/z* calcd. for C_14_H_8_N_2_OS_2_ [M+H]⁺: 285.02; found: 285.34

*7-Methoxy-2-(thiophen-3-yl)benzo[d]imidazo[2,1-b]thiazole-3-carbaldehyde (50)*

Beige powder. ^1^H NMR (400 MHz, DMSO-*d*_6_) δ 9.91 (s, 1H, CHO), 8.88 (d, *J* = 9.2 Hz, 1H, bz), 8.13 (dd, *J* = 2.9, 1.3 Hz, 1H, th), 7.74 – 7.69 (m, 2H, th+bz), 7.59 (dd, *J* = 5.0, 1.3 Hz, 1H, th), 7.16 (dd, *J* = 9.2, 2.7 Hz, 1H, bz), 3.82 (s, 3H, OCH_3_). ^13^C NMR (100 MHz, DMSO-*d*_6_) δ 177.41, 157.20, 153.93, 153.59, 133.32, 130.96, 128.15, 127.26, 127.22, 127.15, 125.74, 118.16, 114.12, 108.65, 55.80. MW = 314.28. Yield 95%. MS (ESI): *m/z* calcd. for C_15_H_10_N_2_O_2_S_2_ [M+H]⁺: 315.03; found: 315.10.

*2-Methylbenzo[d]imidazo[2,1-b]thiazole-3-carbaldehyde (51)*

Orange Powder. ^1^H NMR (600 MHz, DMSO-*d*_6_) δ 9.87 (s, 1H, CHO), 8.96 (d, *J* = 8.3, Hz, 1H), 8.09 (d, *J* = 8.3 Hz, 1H, bz), 7.59 (t, *J* = 8.3 Hz, 1H, bz), 7.48 (t, *J* = 8.3 Hz, 1H, bz), 2.62 (s, 3H, CH_3_). ^13^C NMR (150 MHz, DMSO-*d*_6_) δ 176.63, 159.46, 153.79, 133.07, 129.09, 126.69, 126.49, 125.56, 124.79, 117.08, 14.33. MW = 216.26. Yield 90%. MS (ESI): *m/z* calcd. for C_11_H_8_N_2_OS [M+H]⁺: 217.05; found: 217.08.


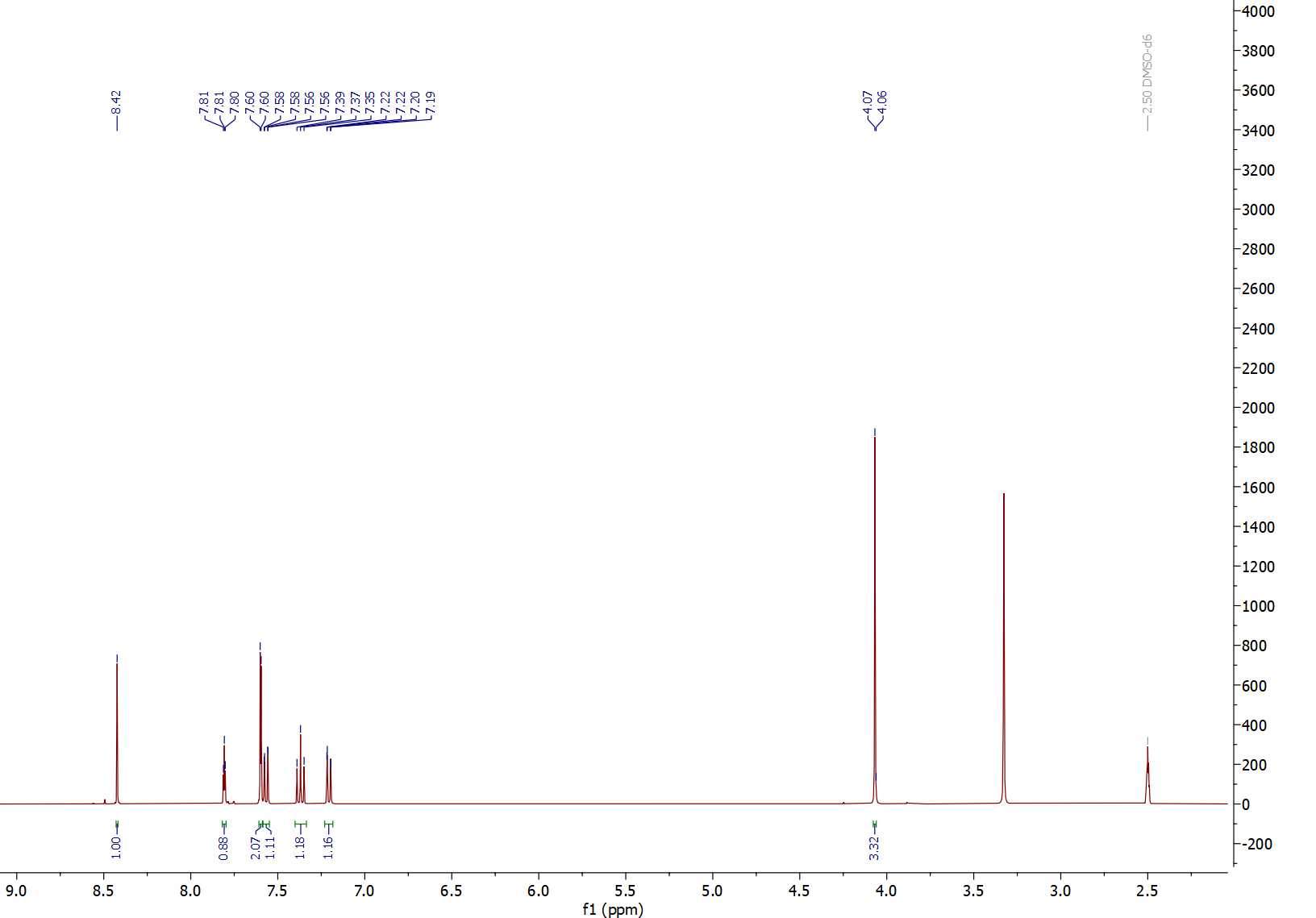


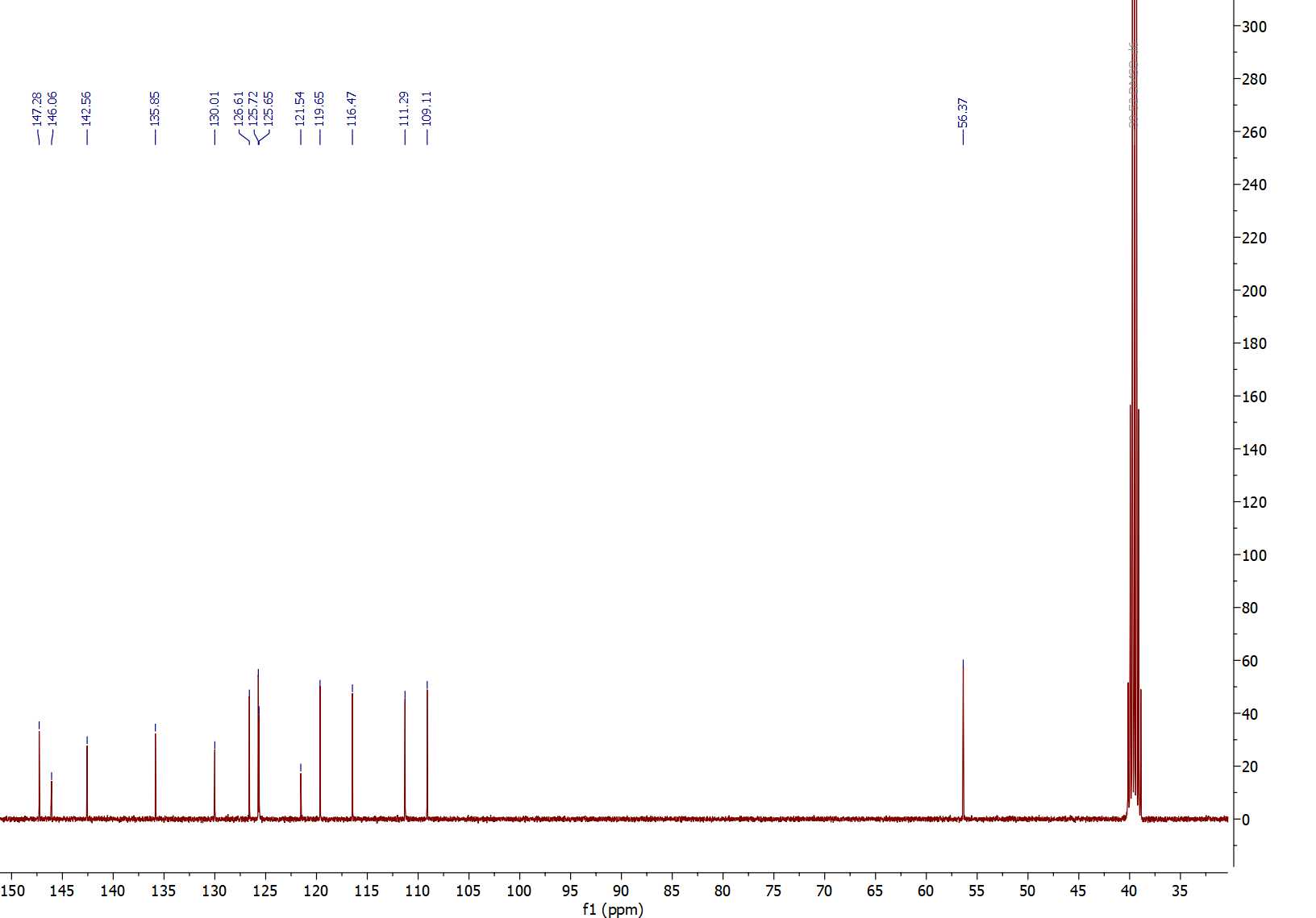


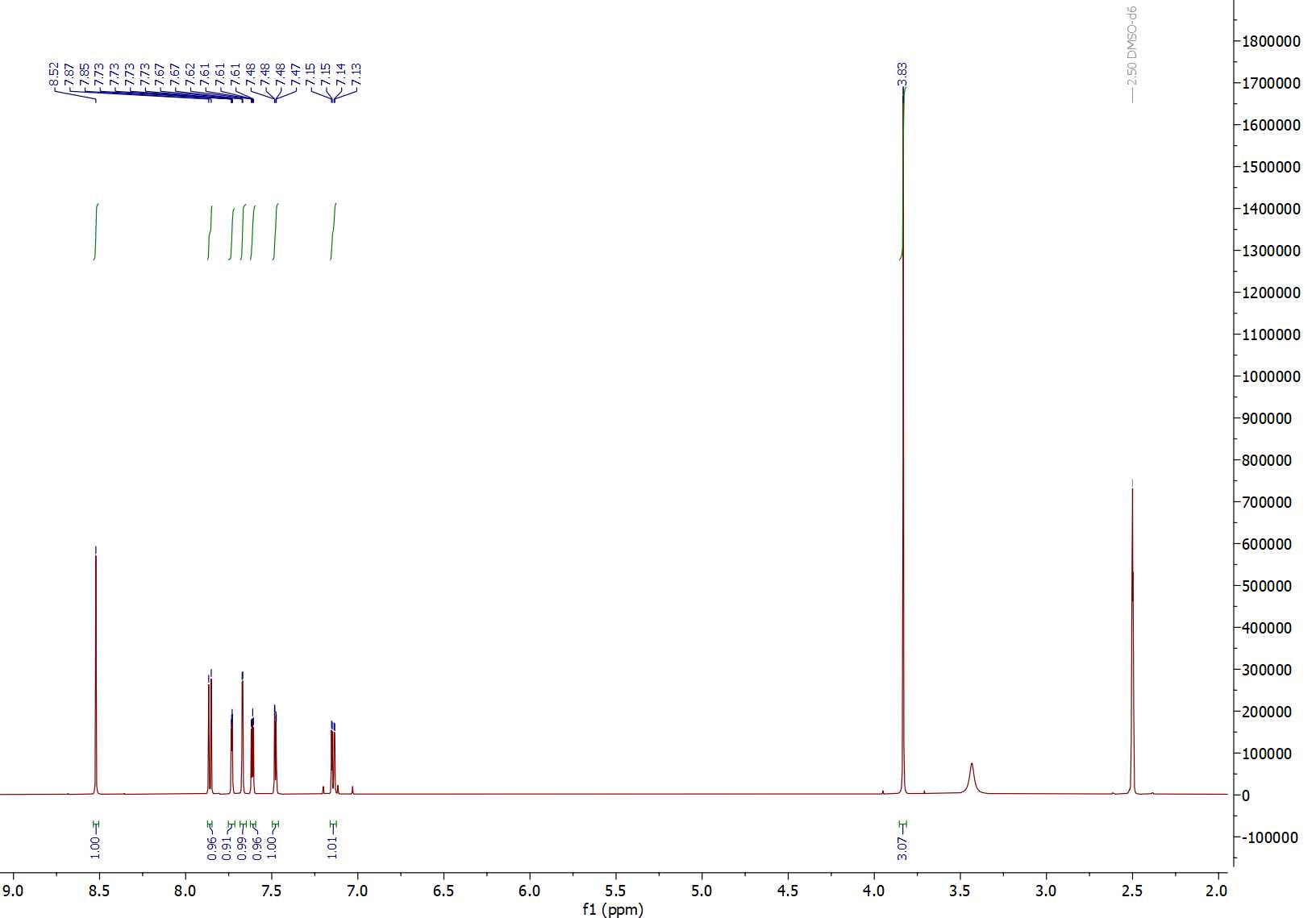


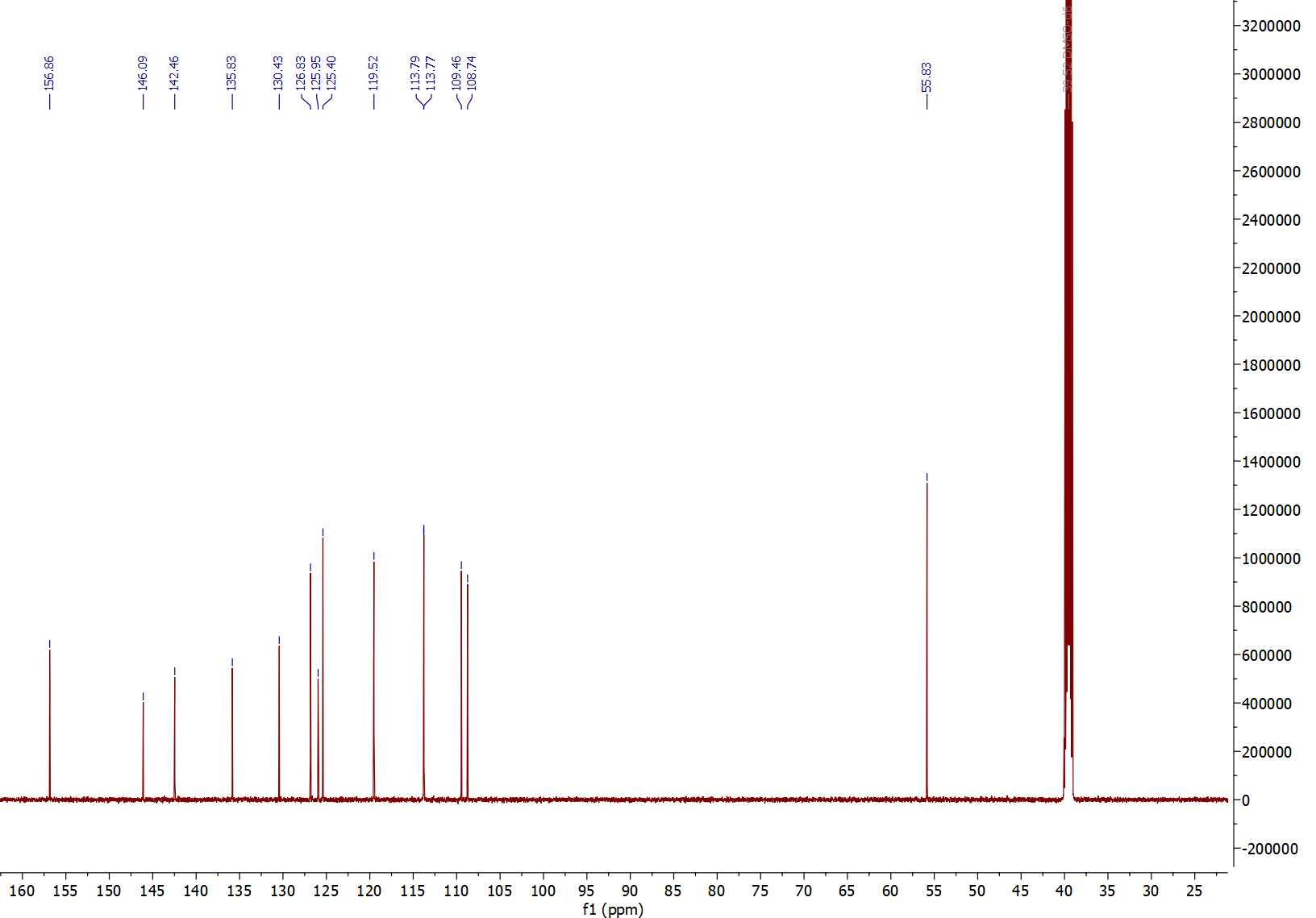


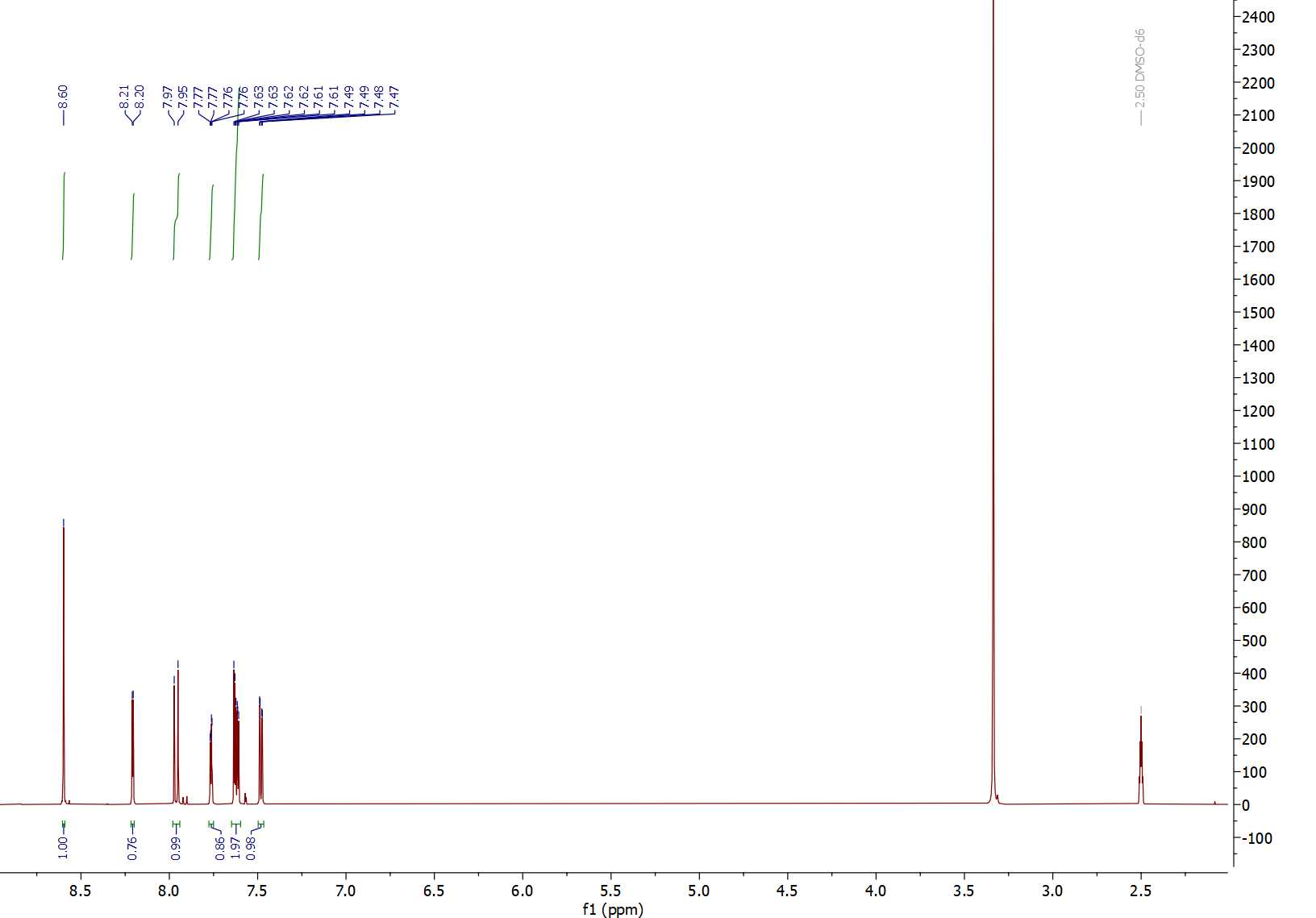


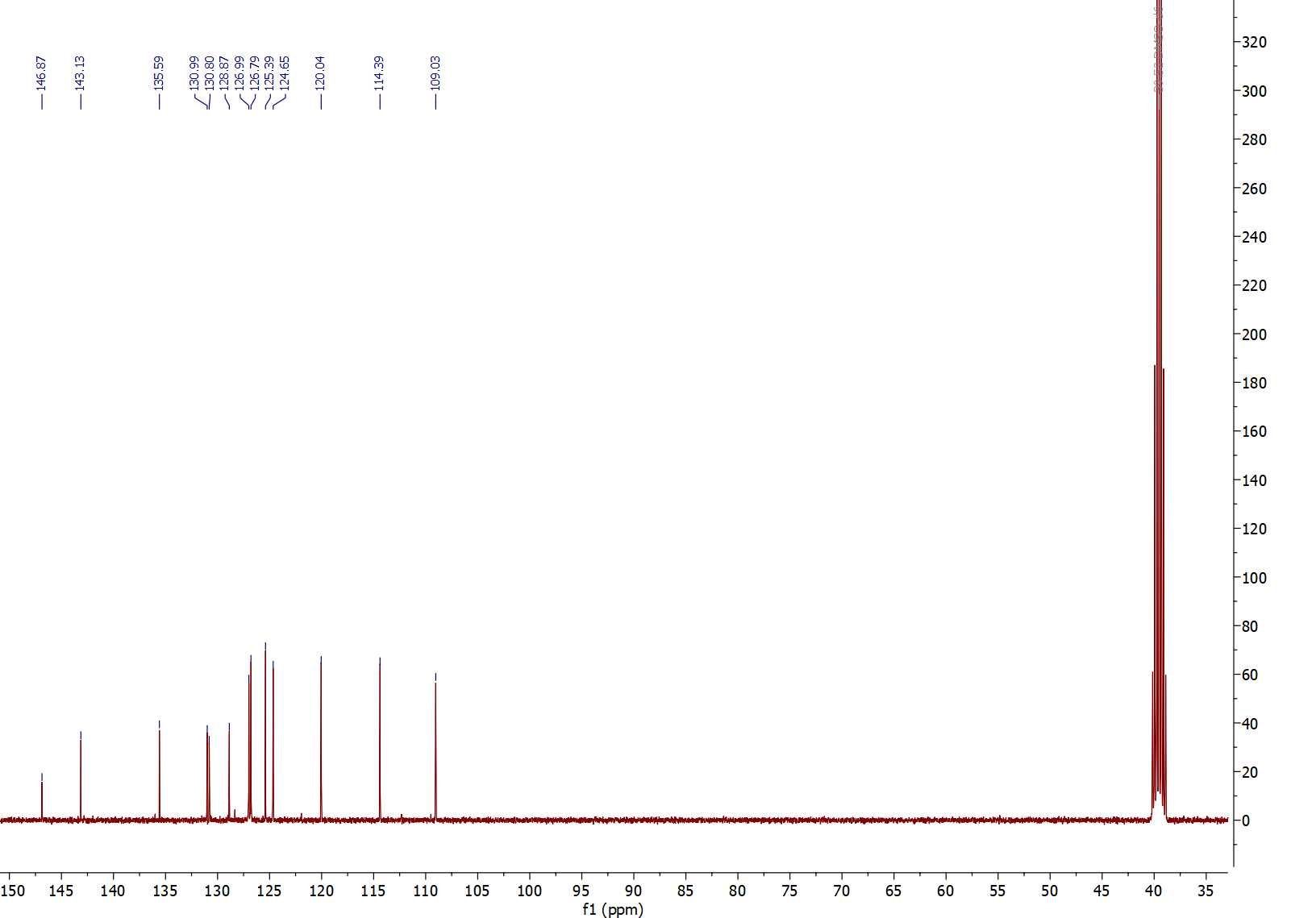


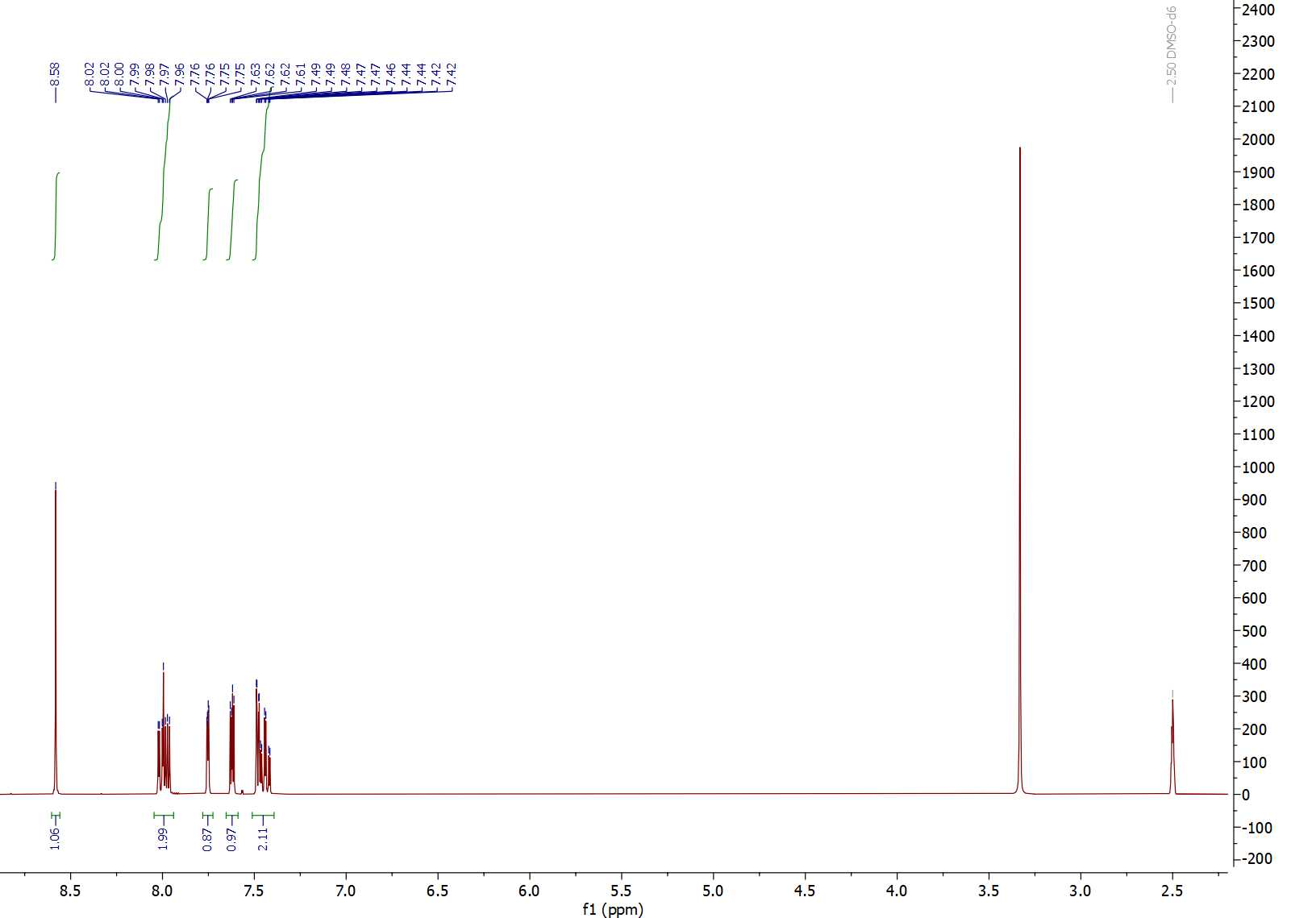


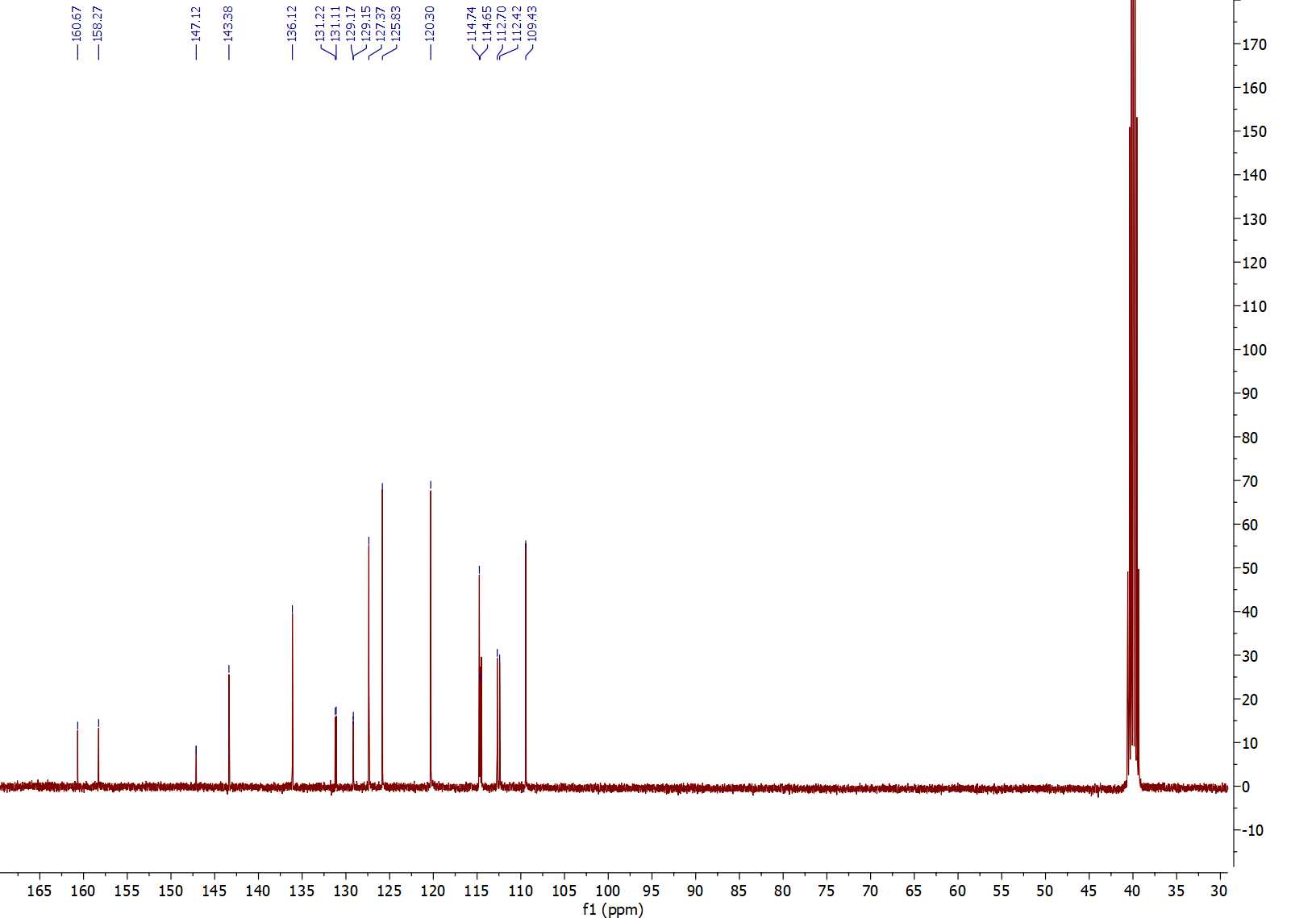


**^1^H-^13^C HSQC NMR spectrum of compound 5 (600 MHz, DMSO-d_6_)**


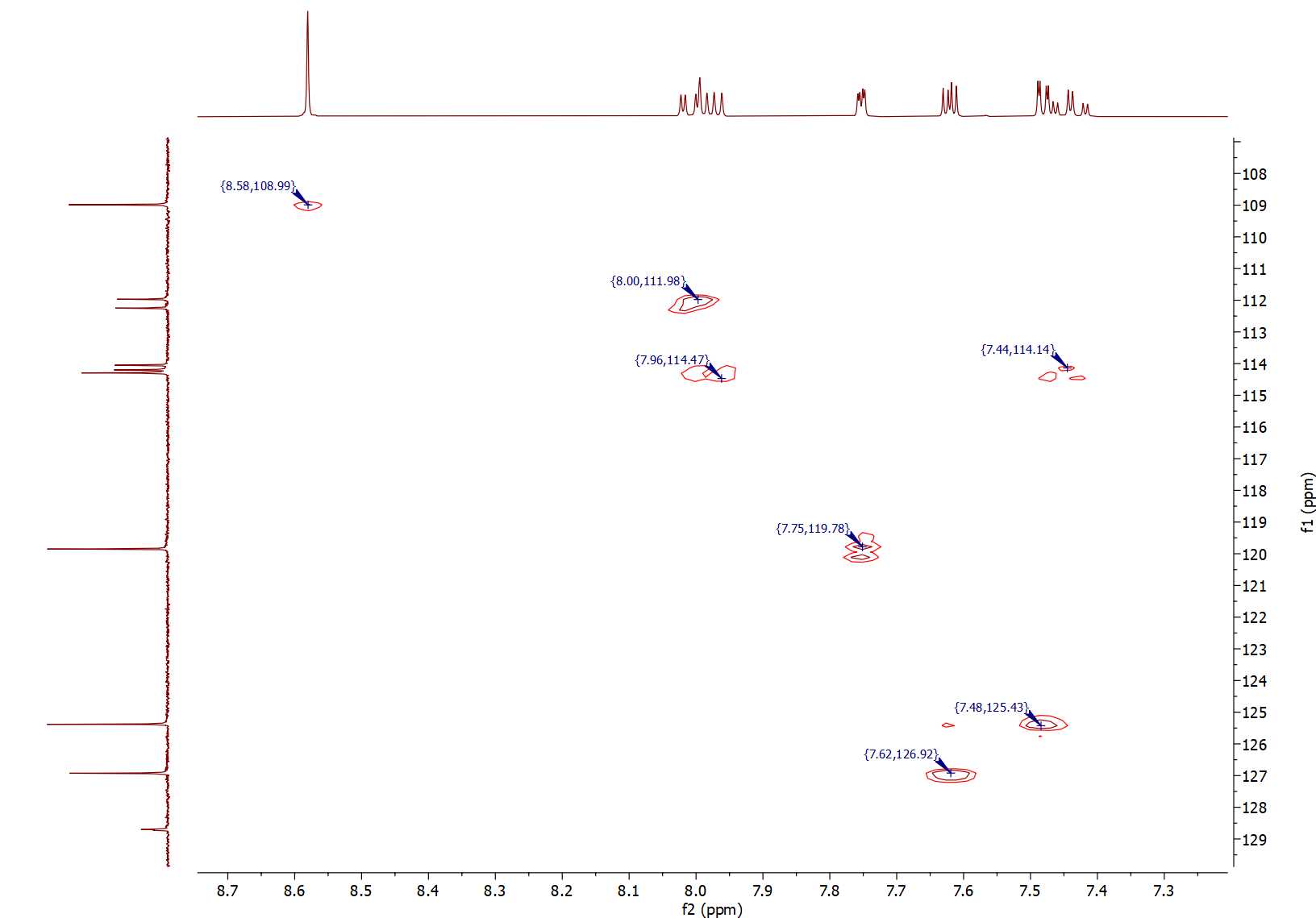


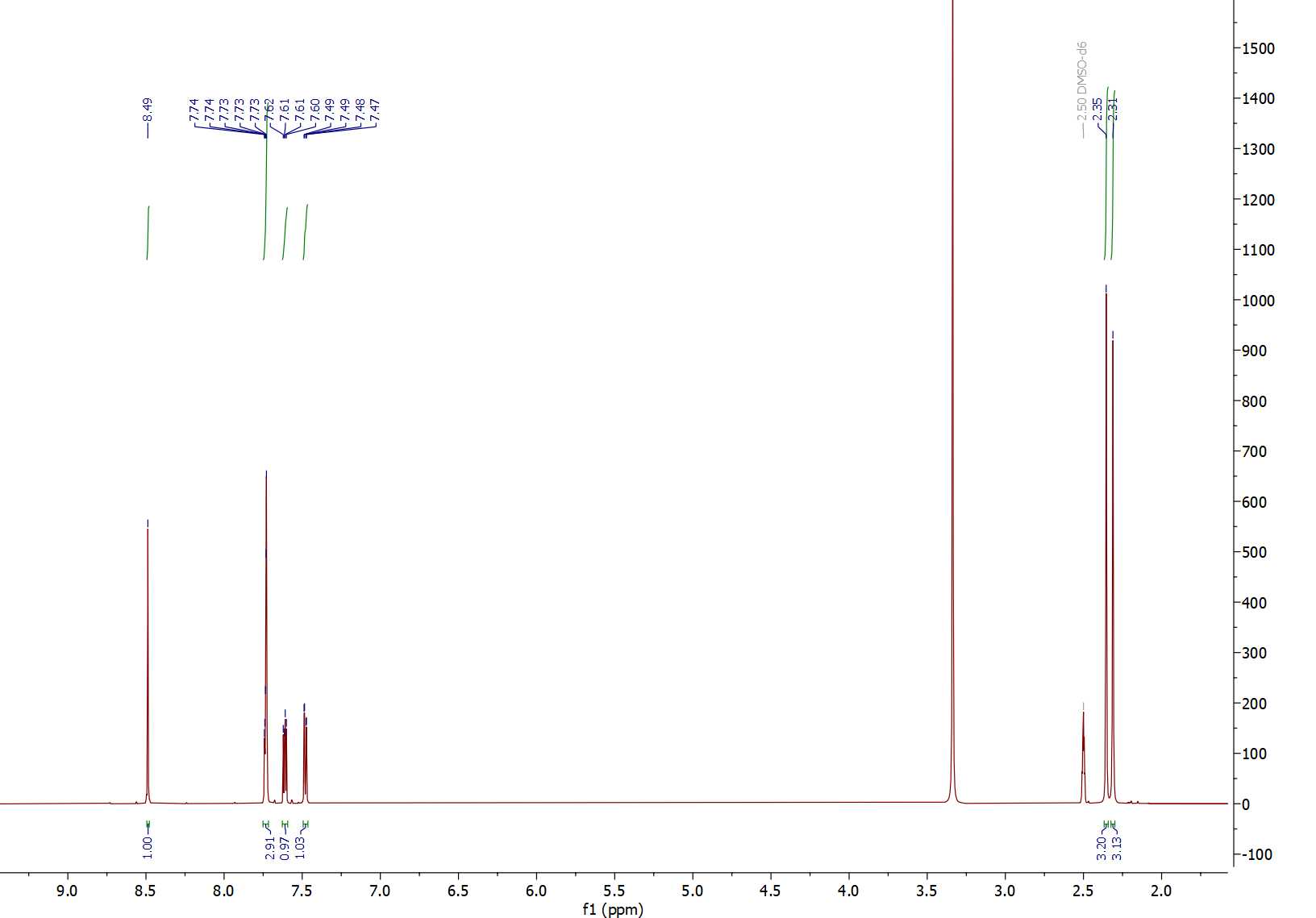


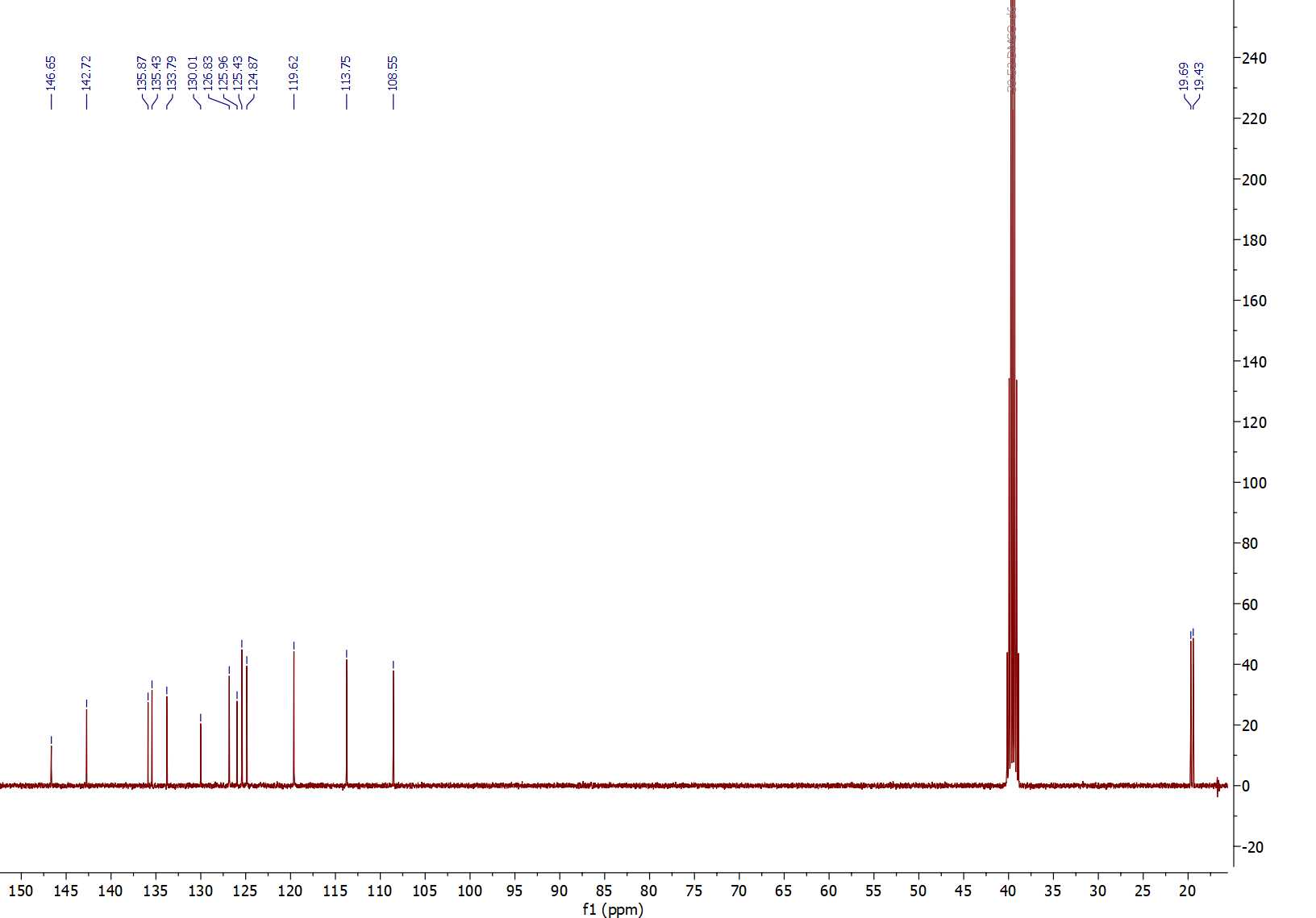


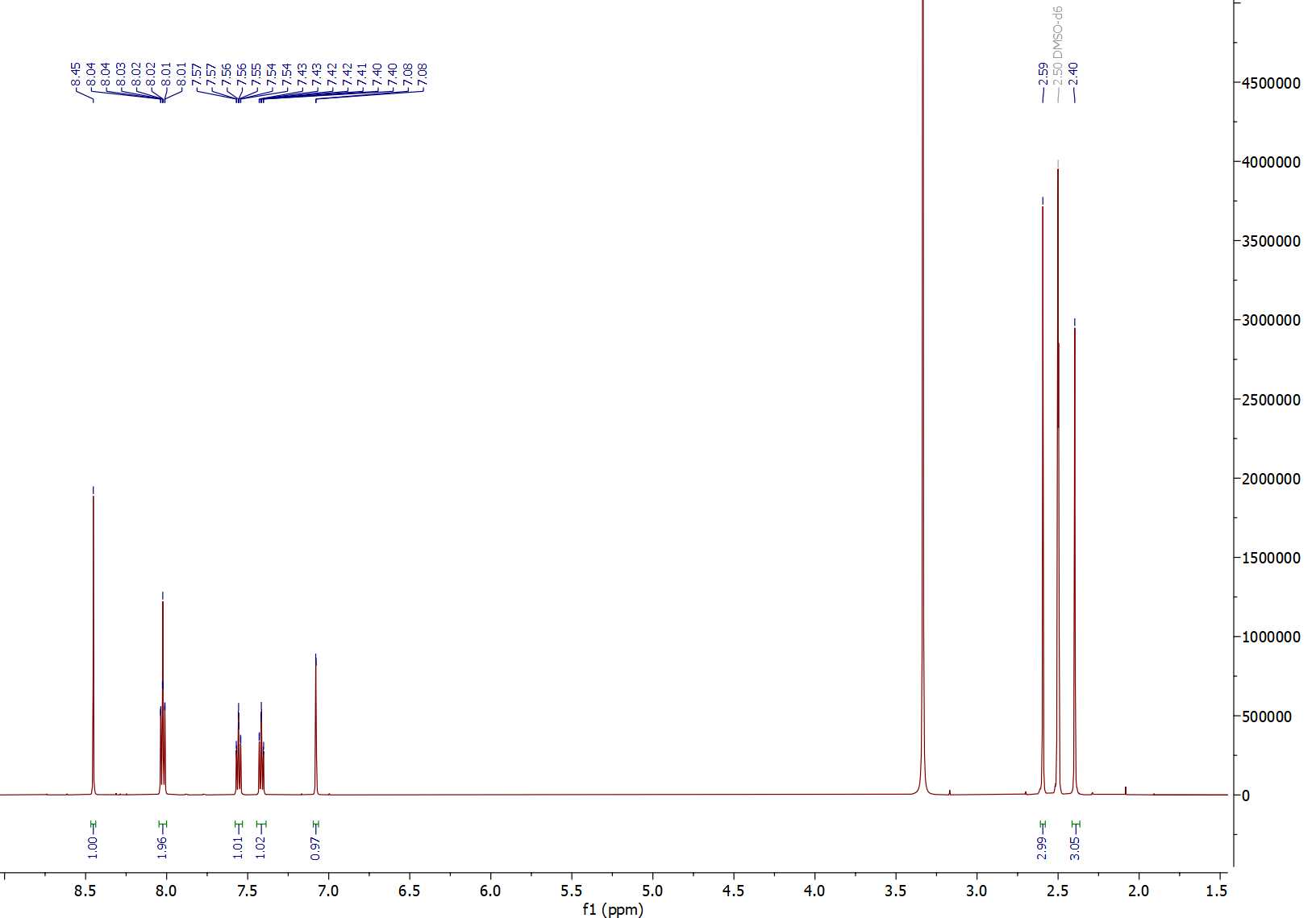


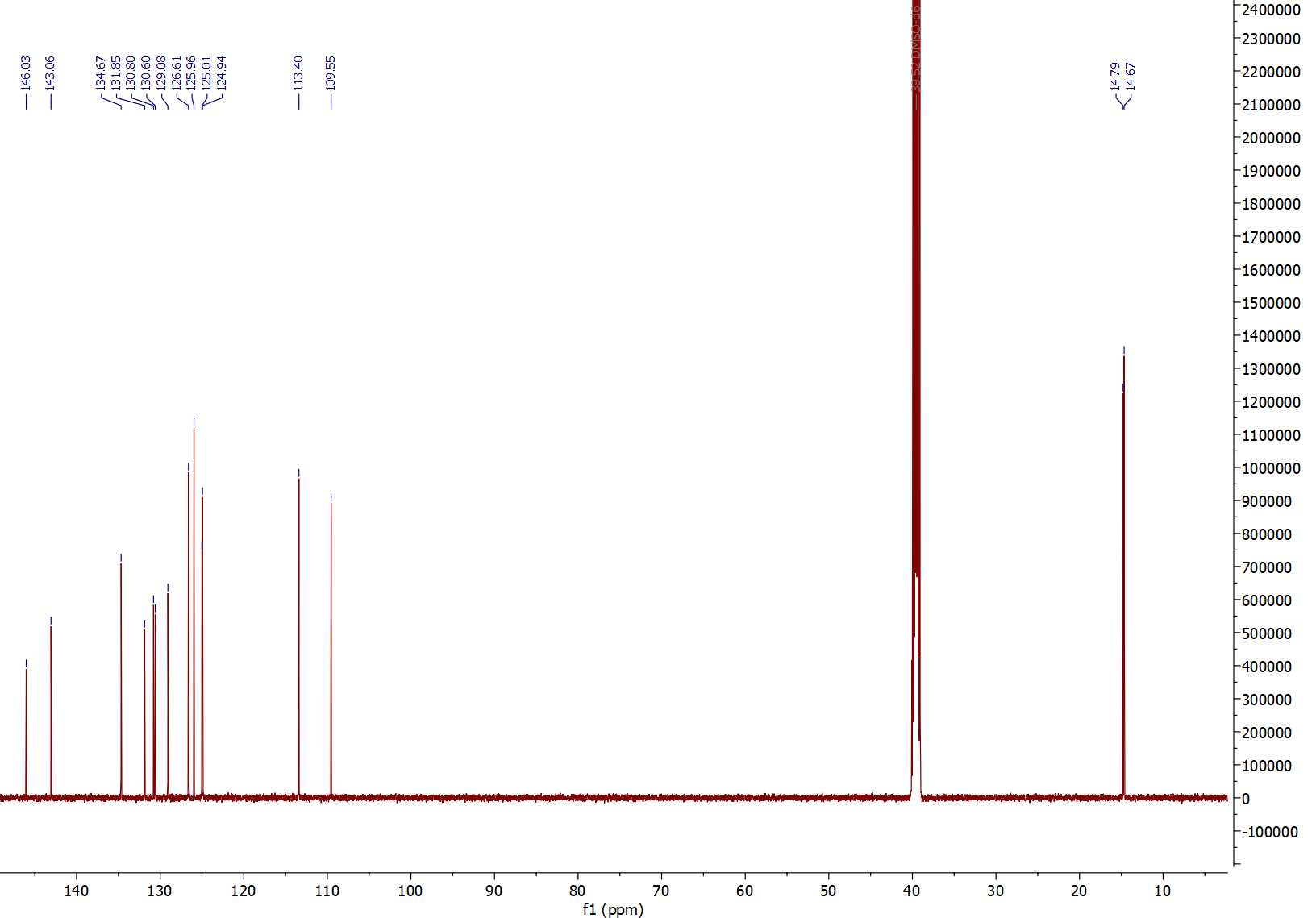


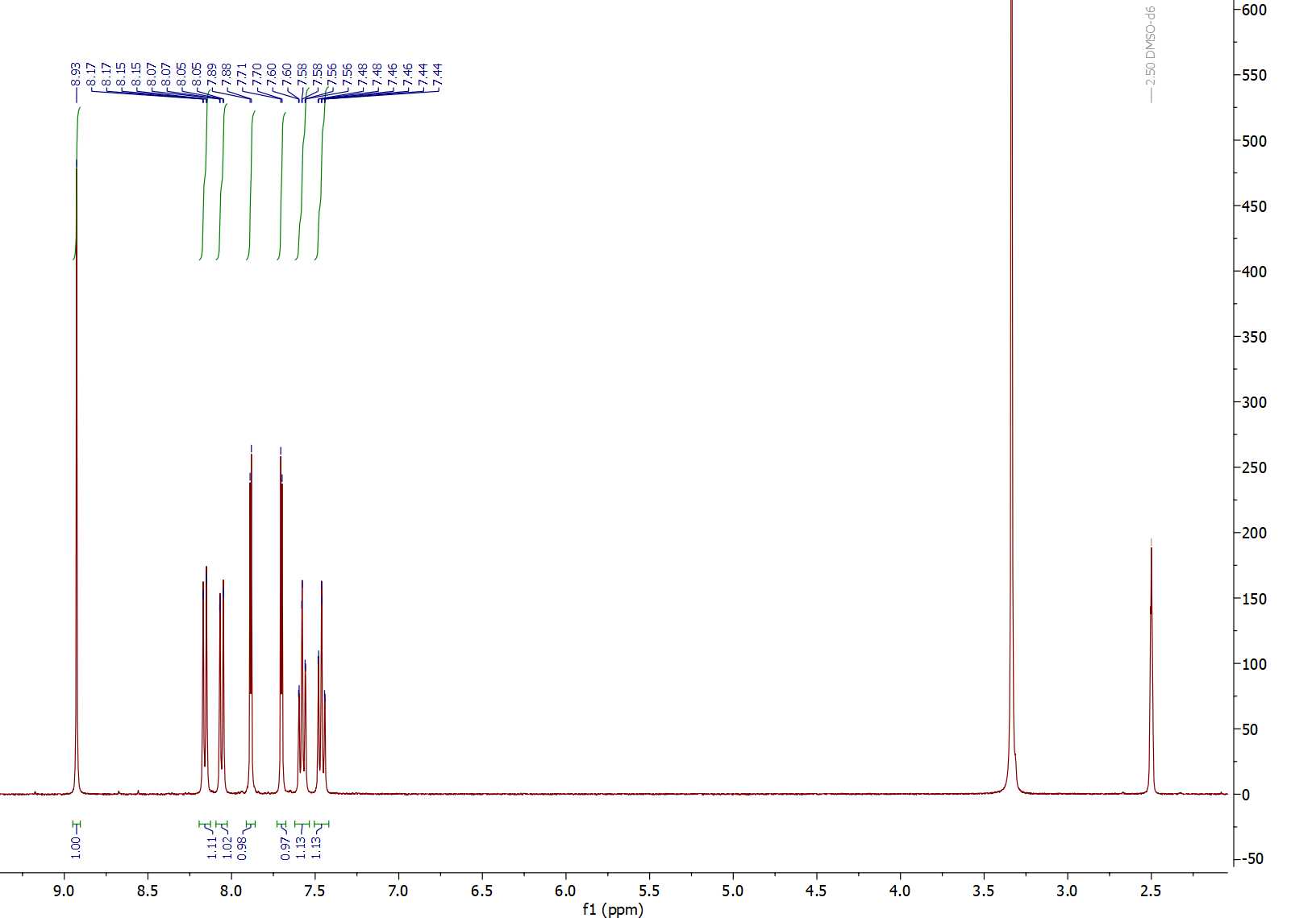


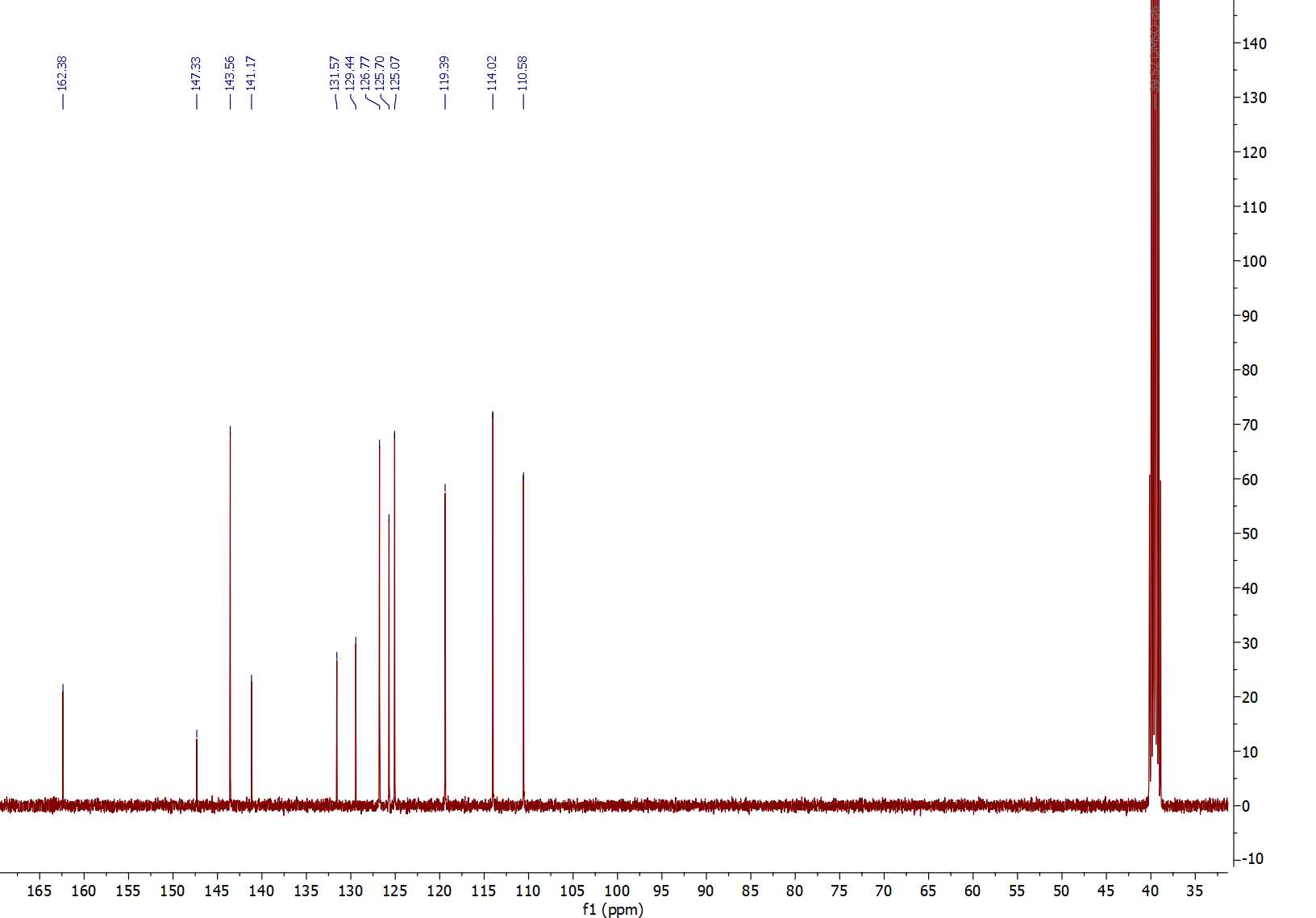


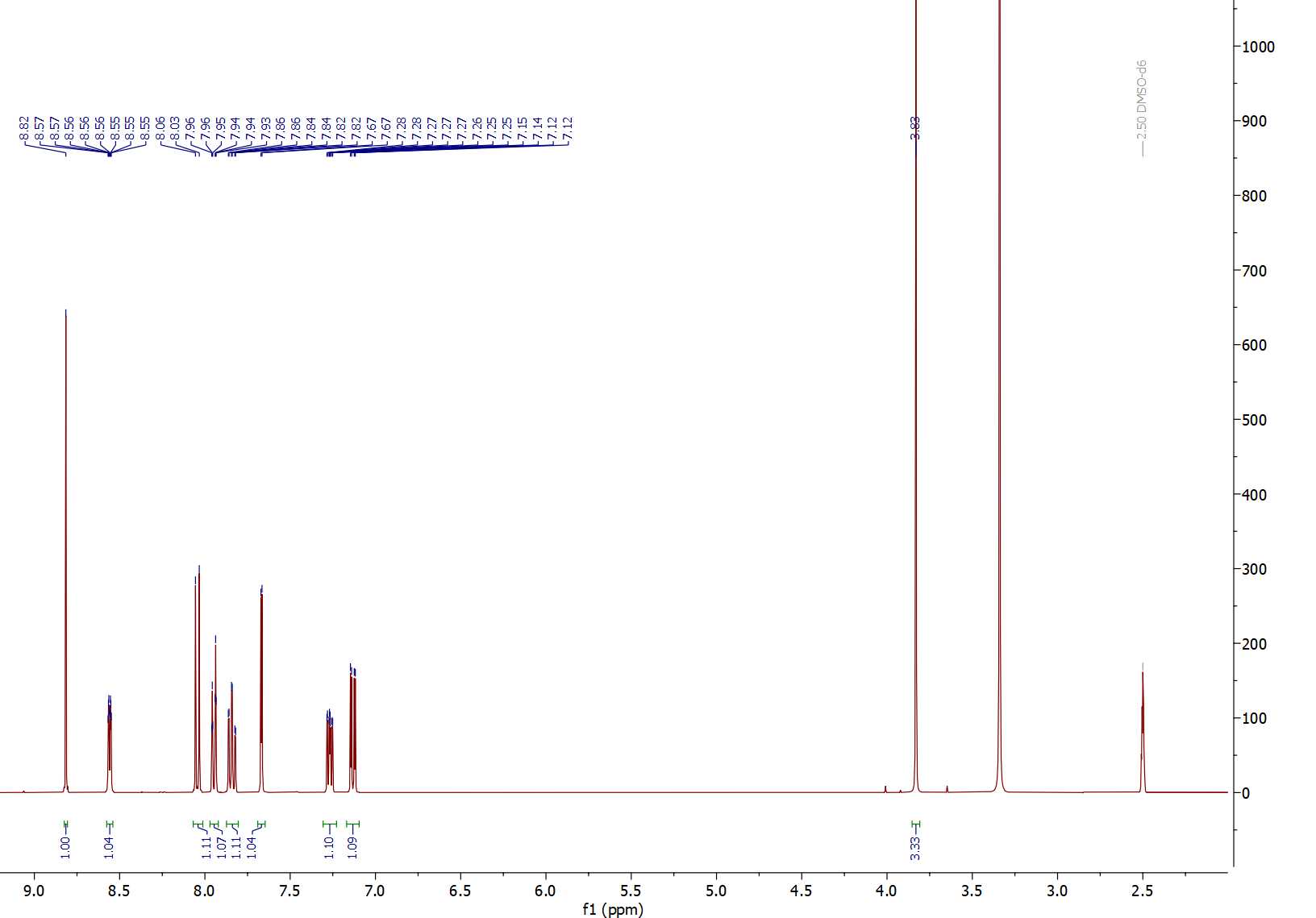


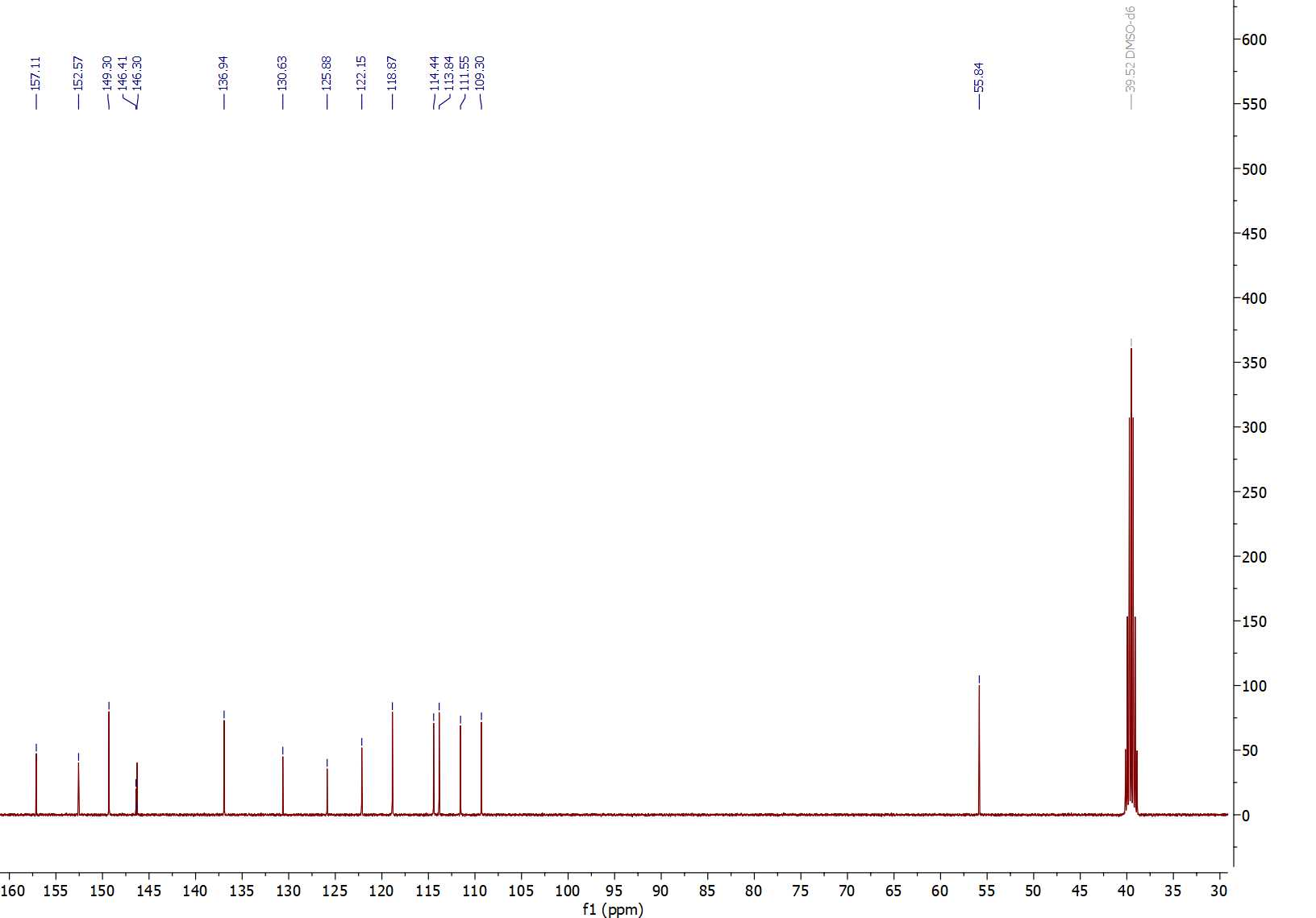


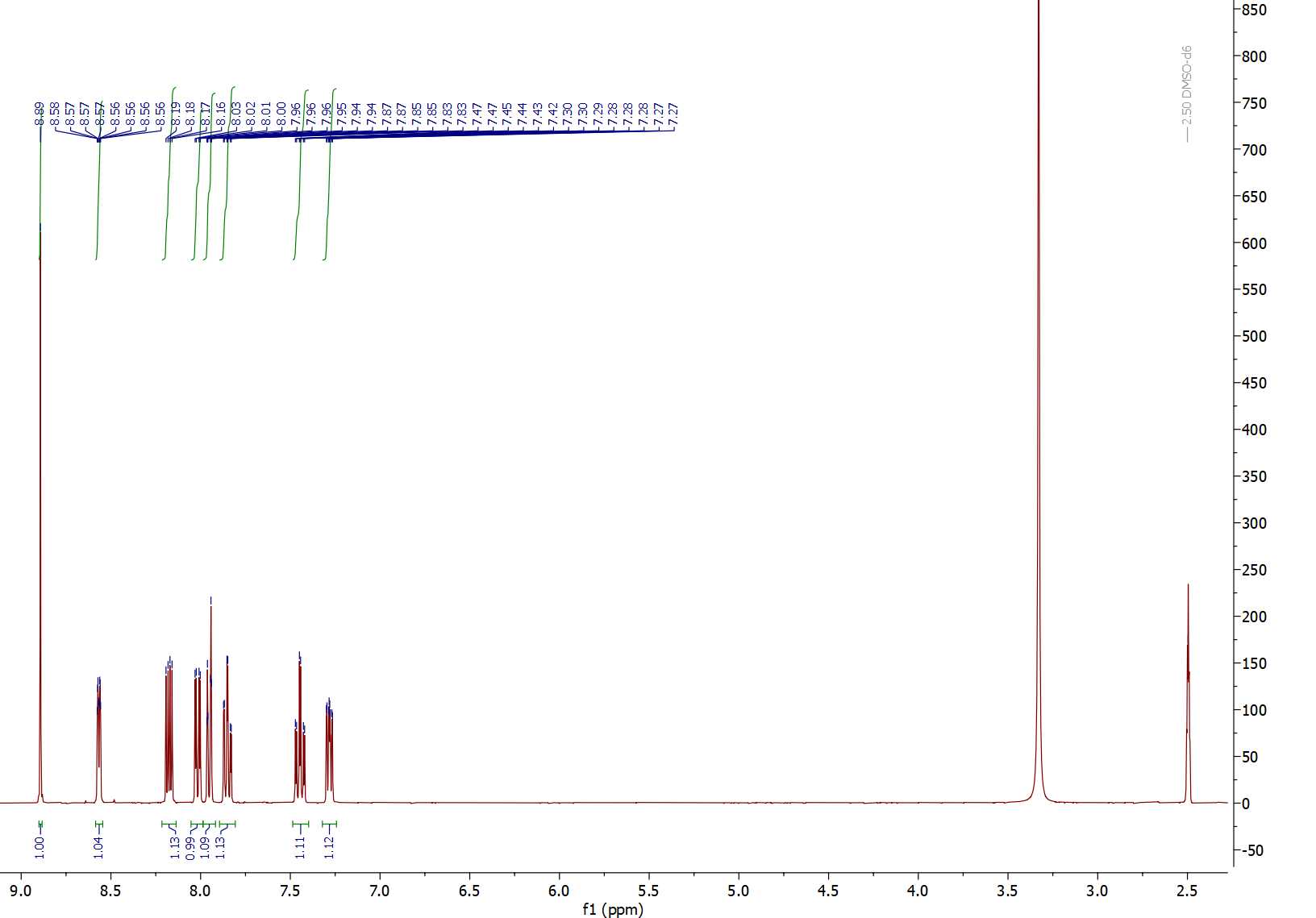


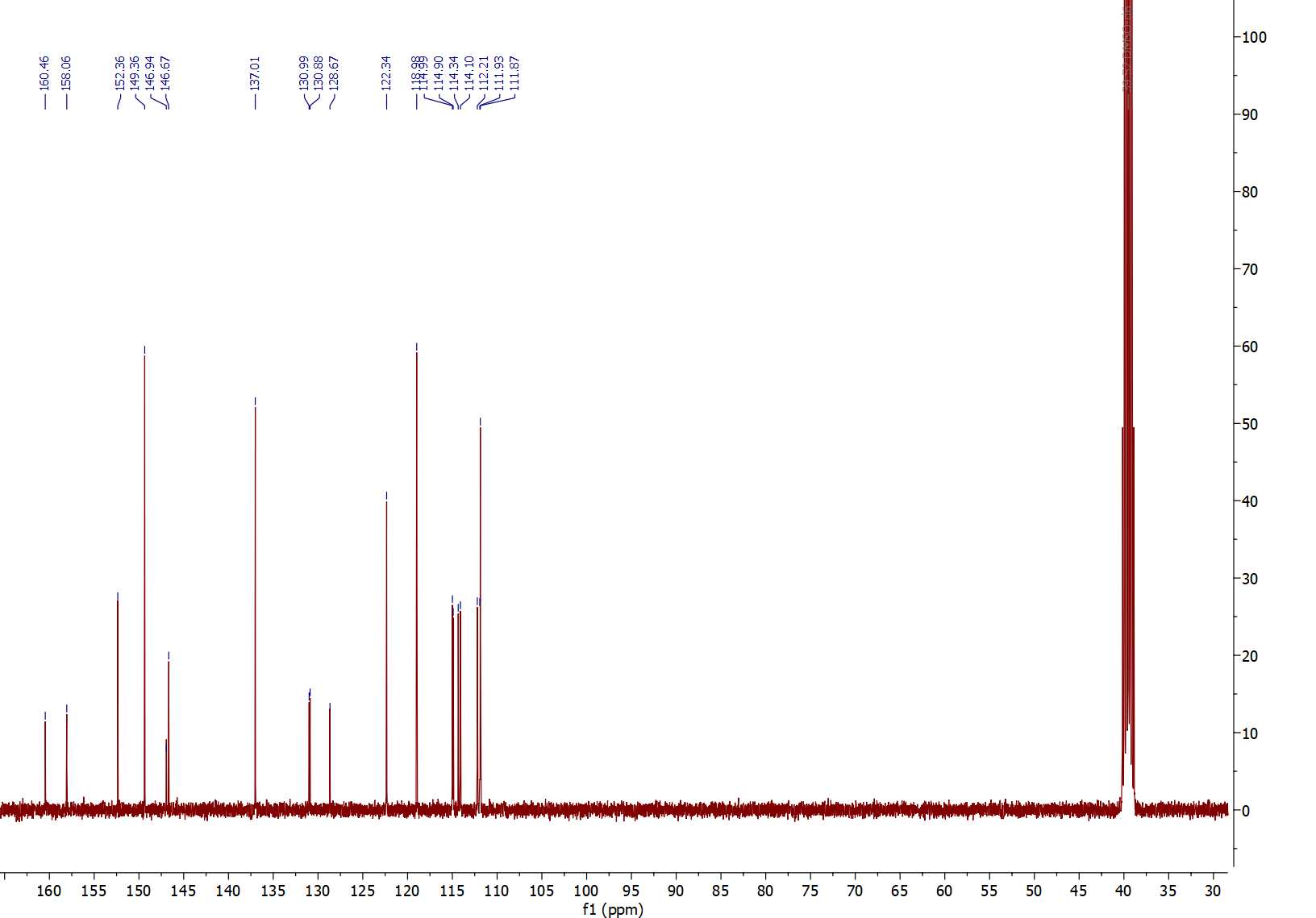


**^1^H-^13^C HSQC NMR spectrum of compound 15 (600 MHz, DMSO-d_6_)**


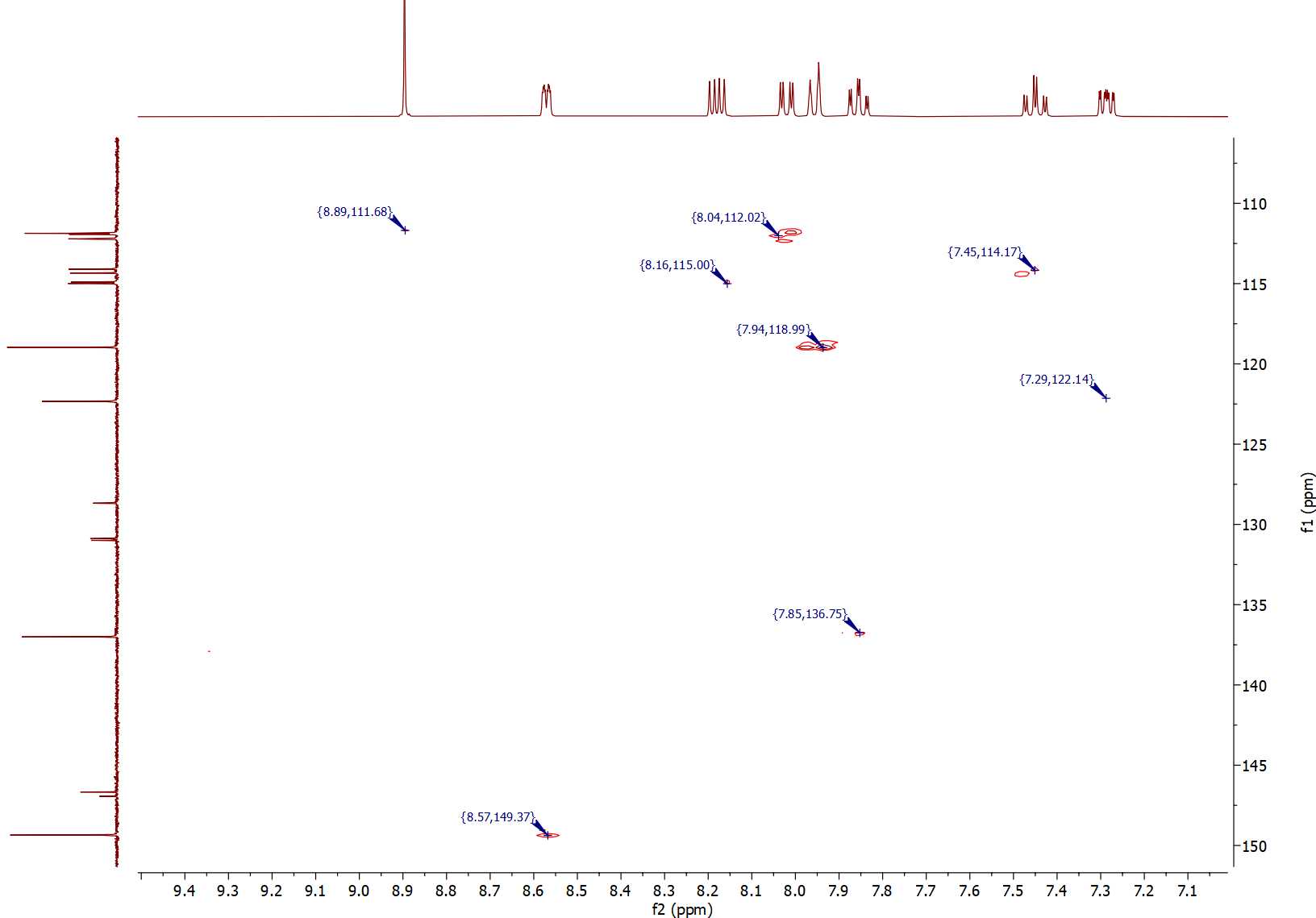


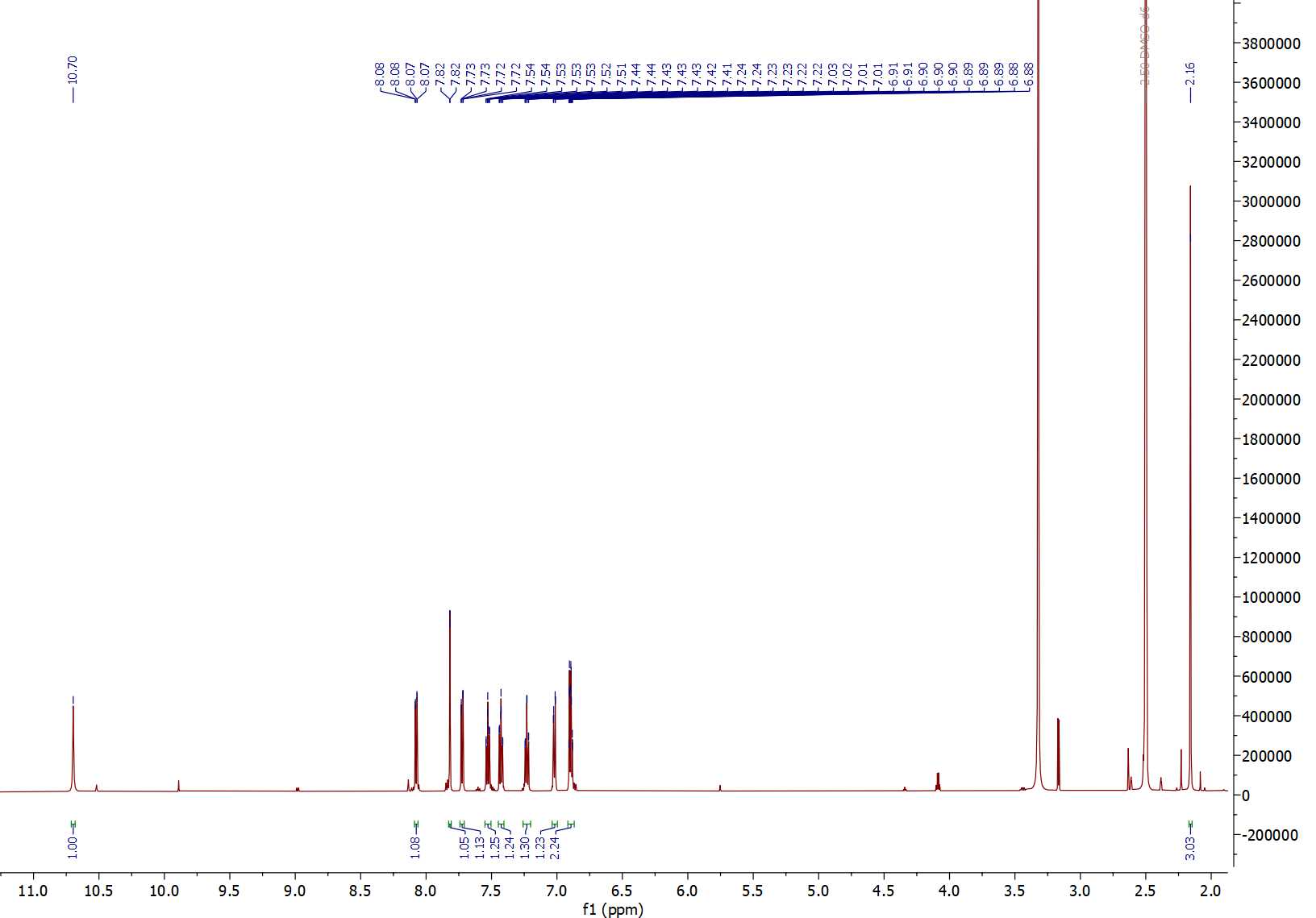


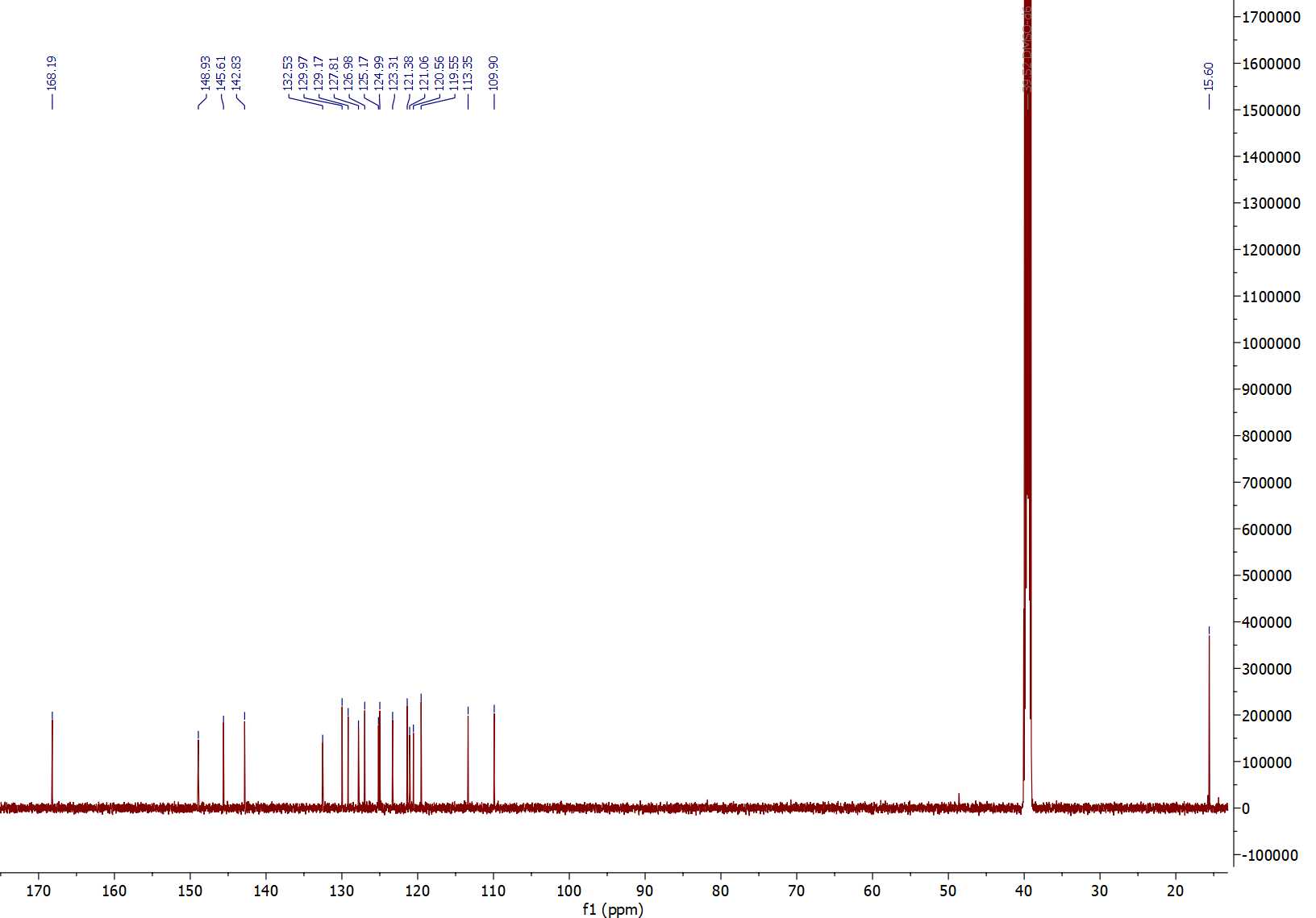


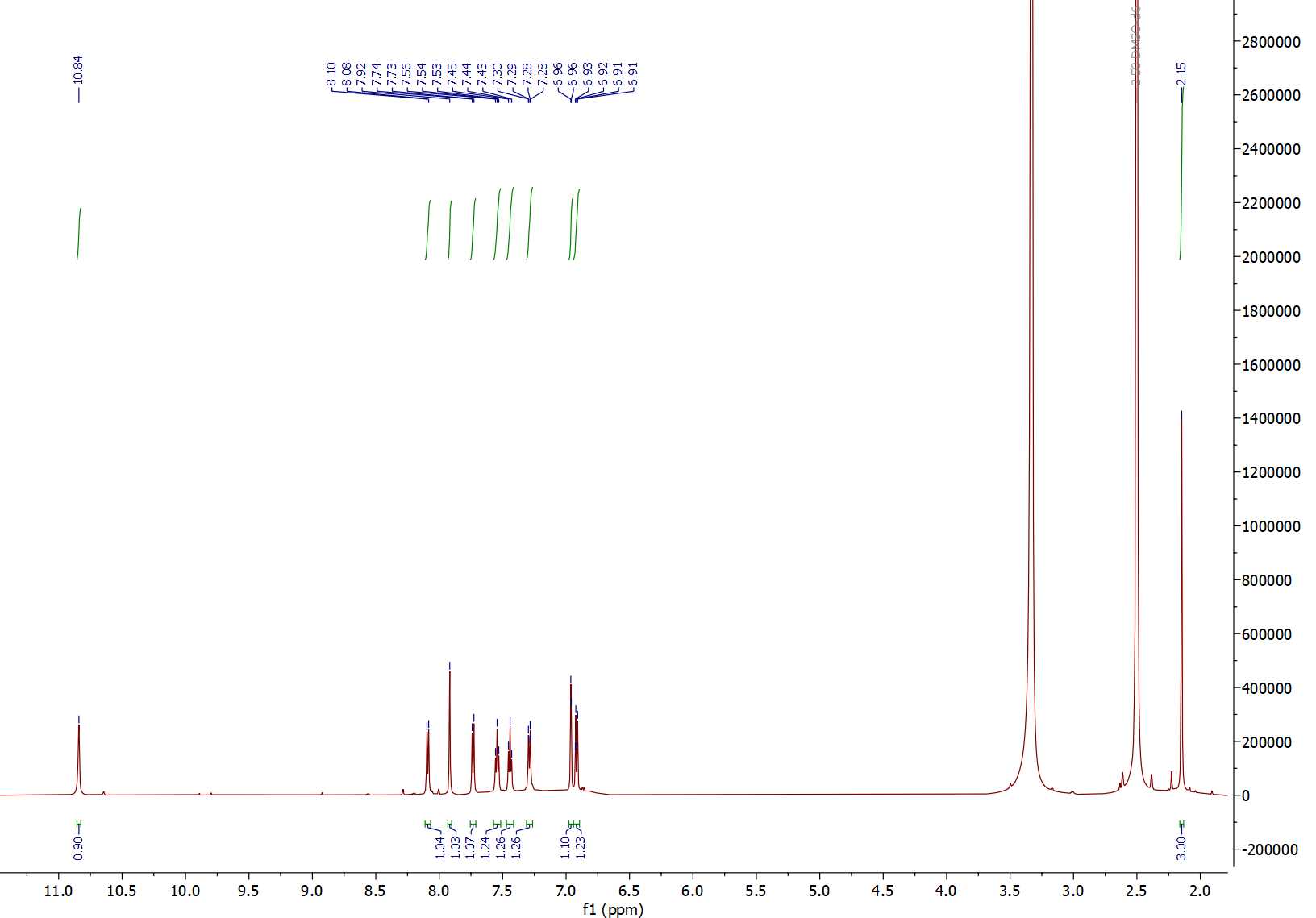


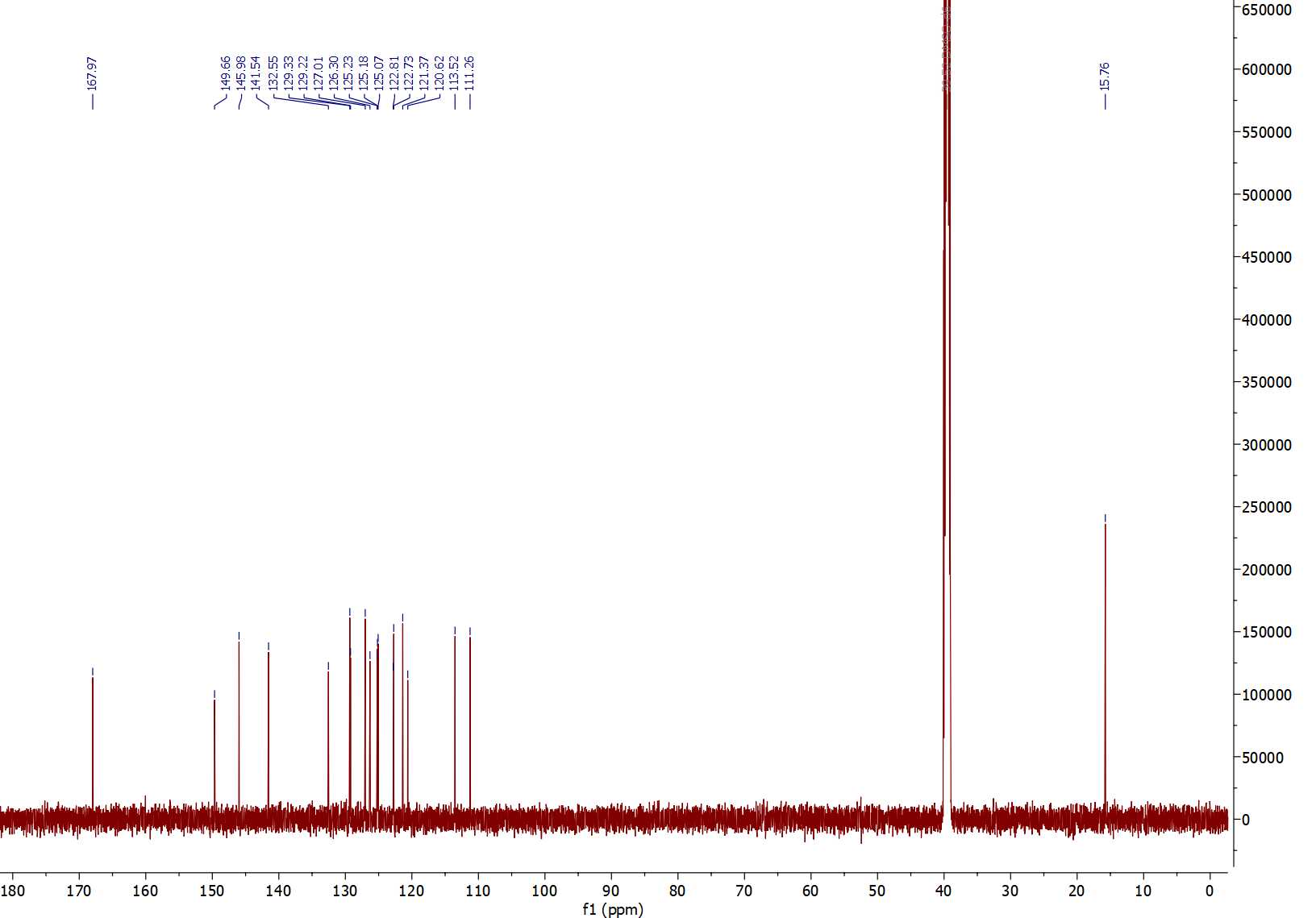


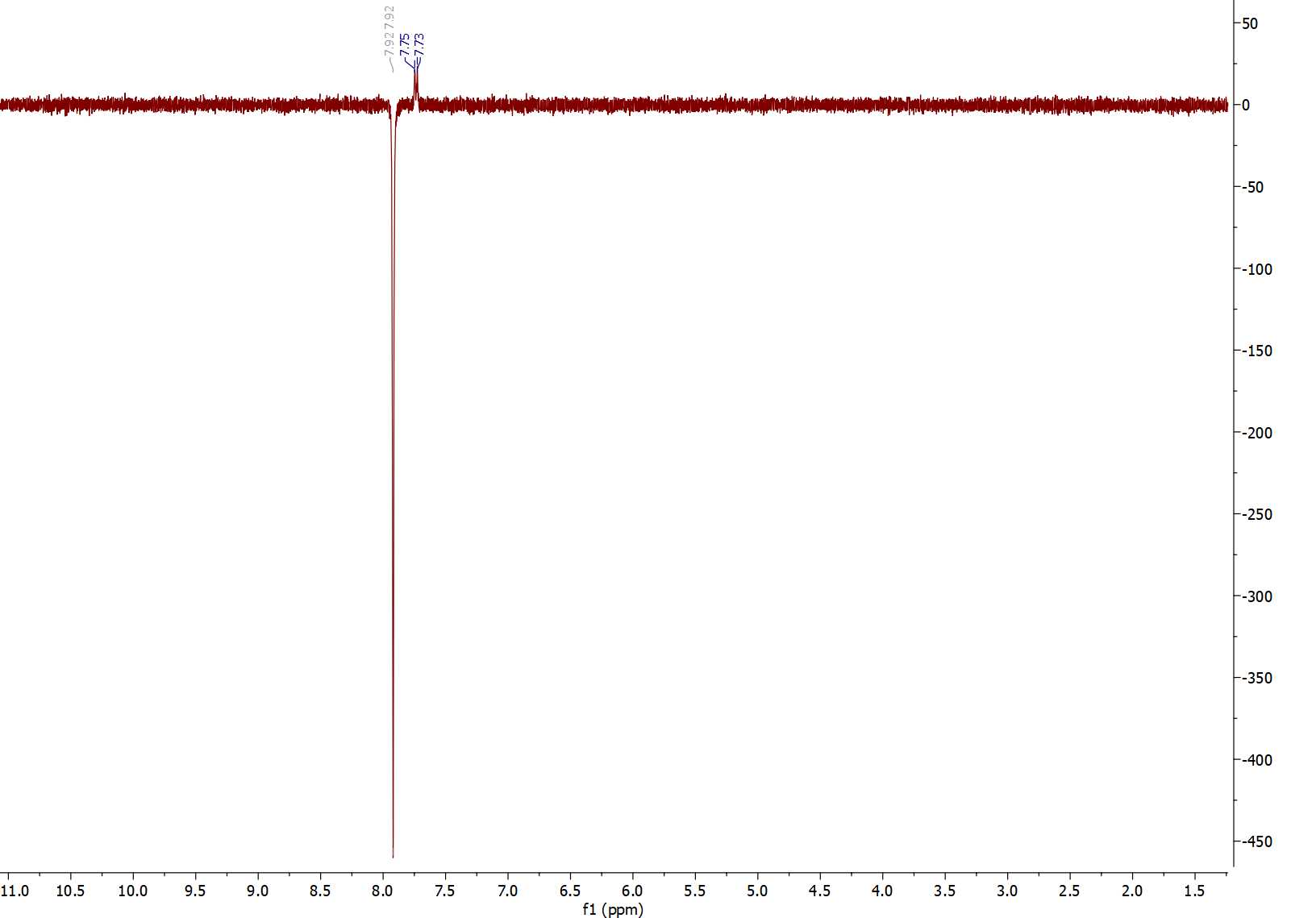


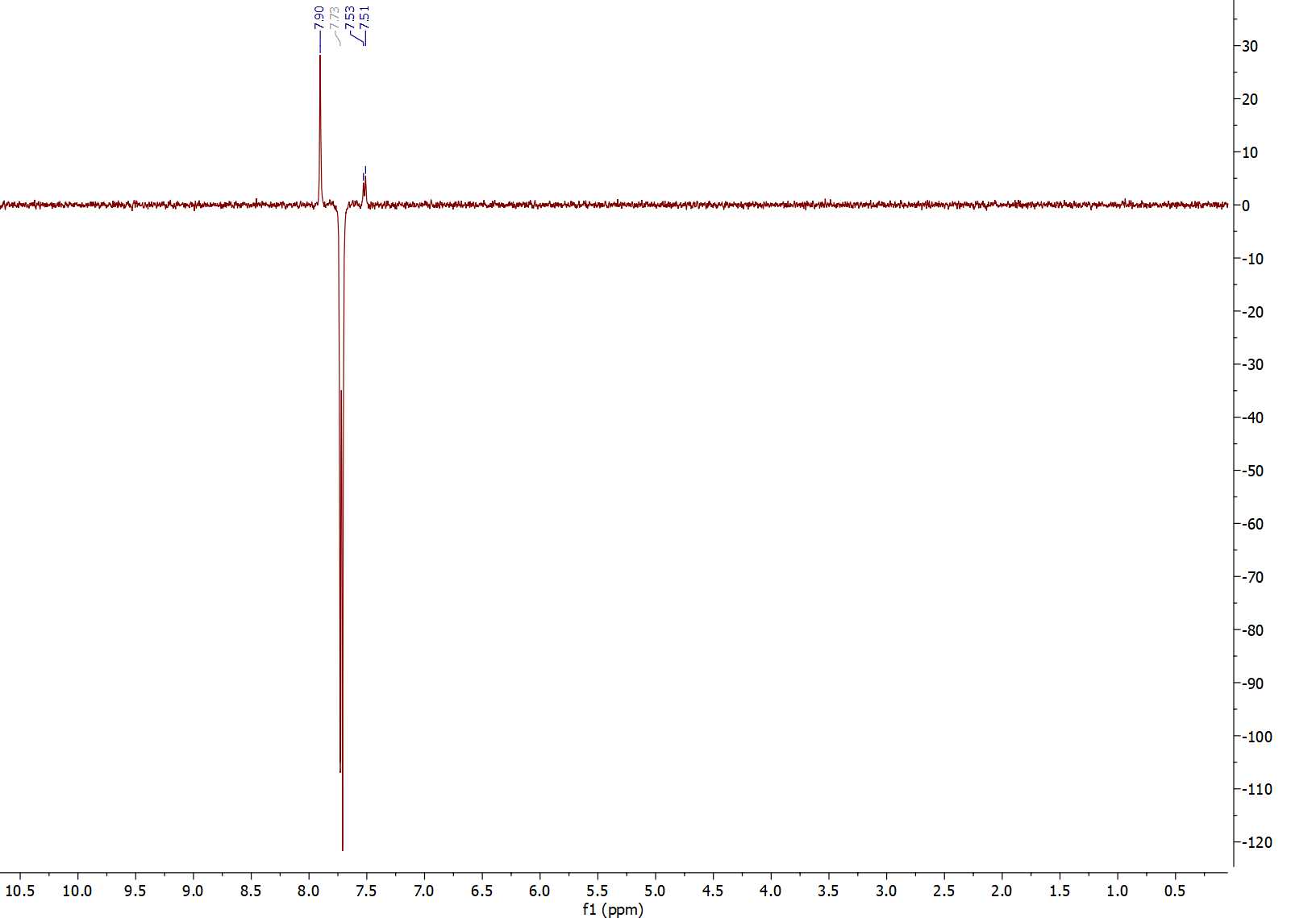


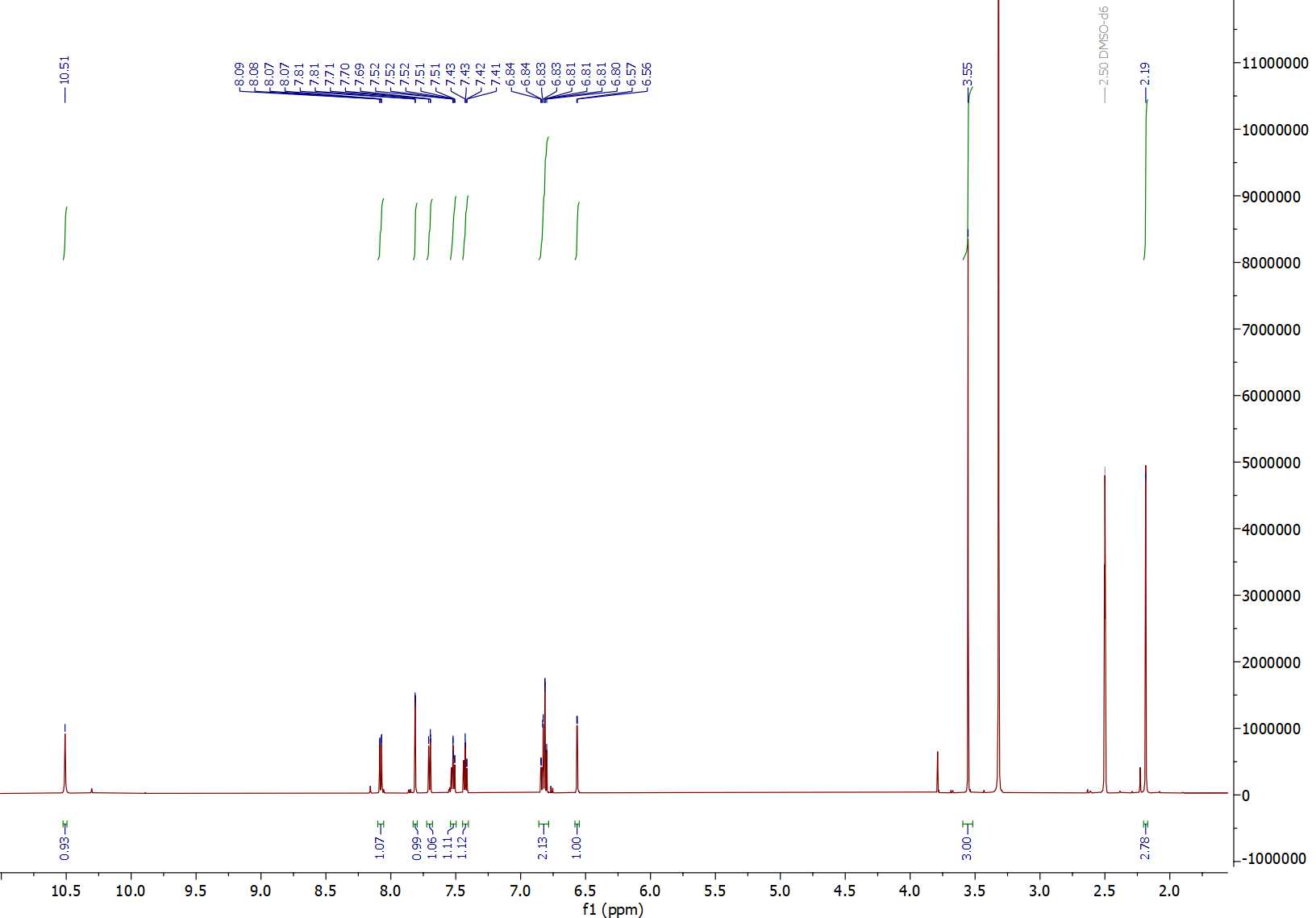


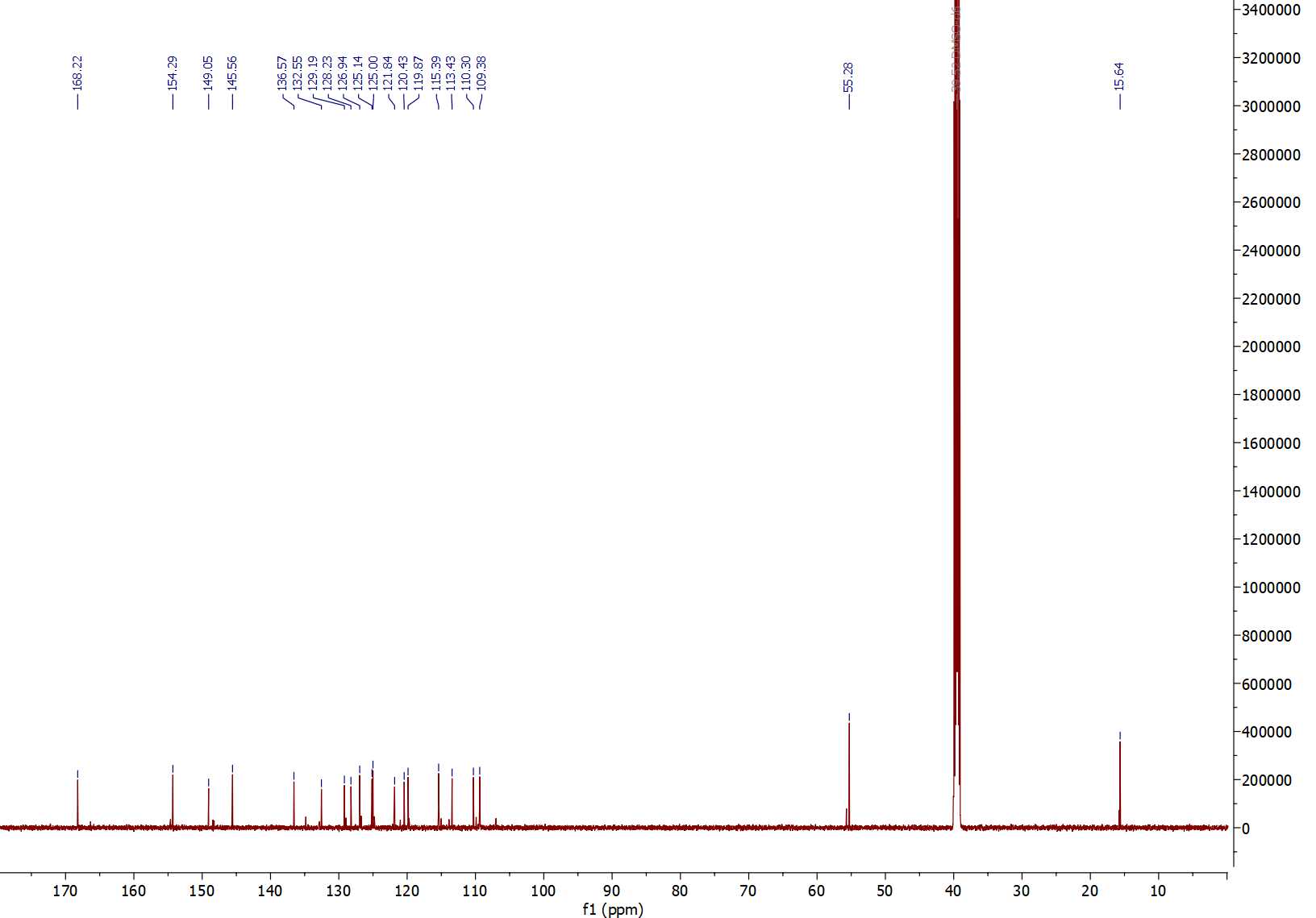


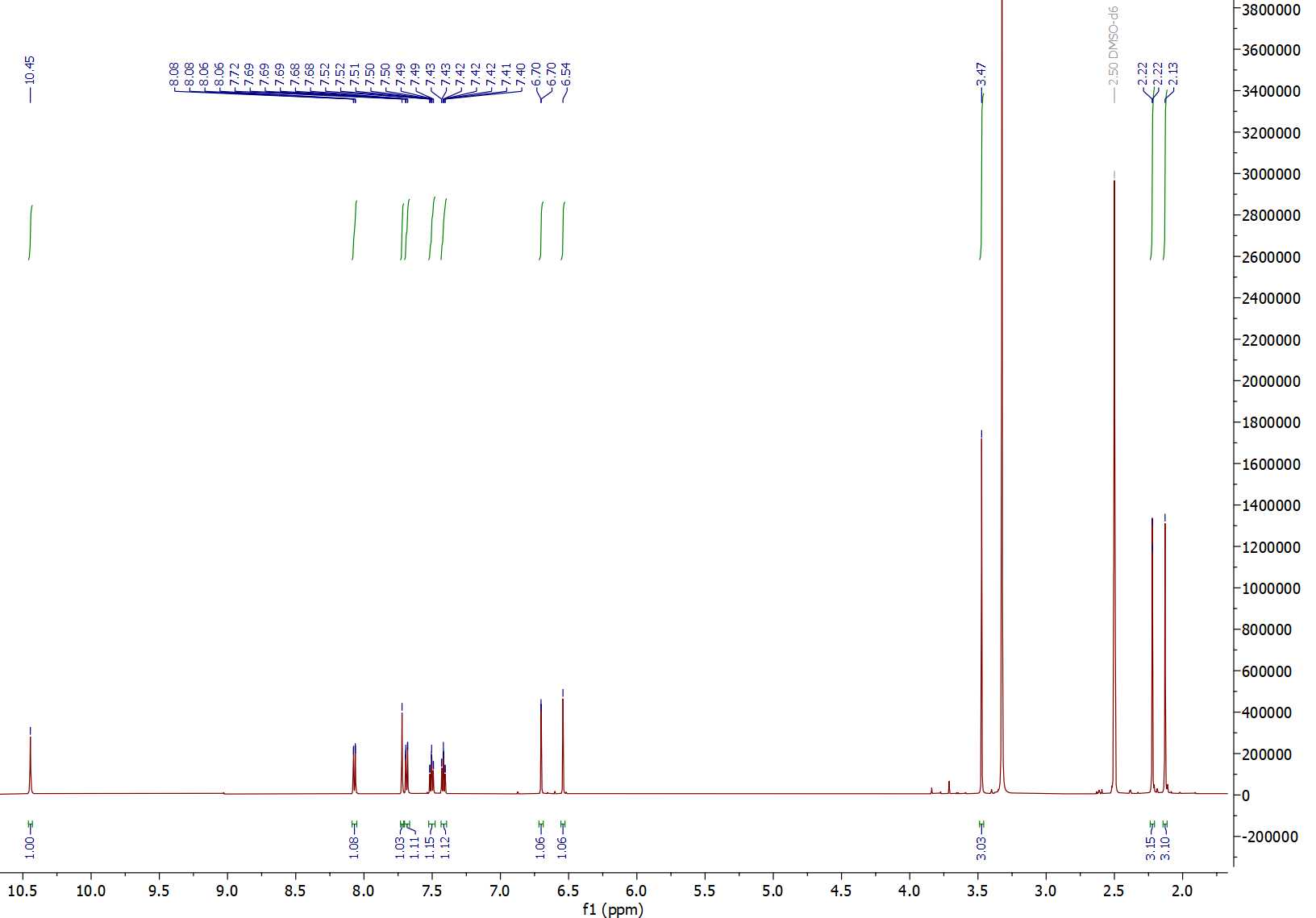


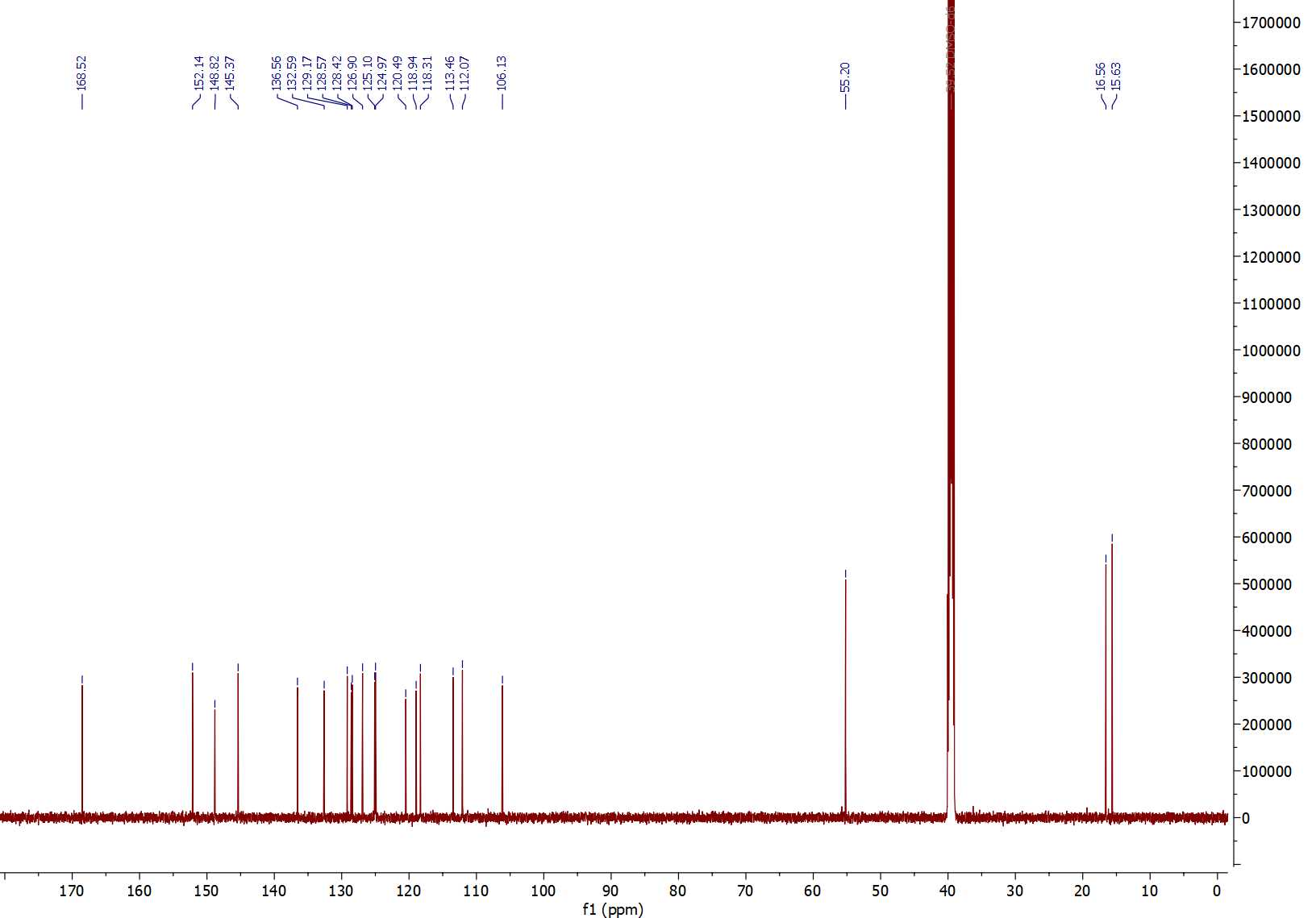


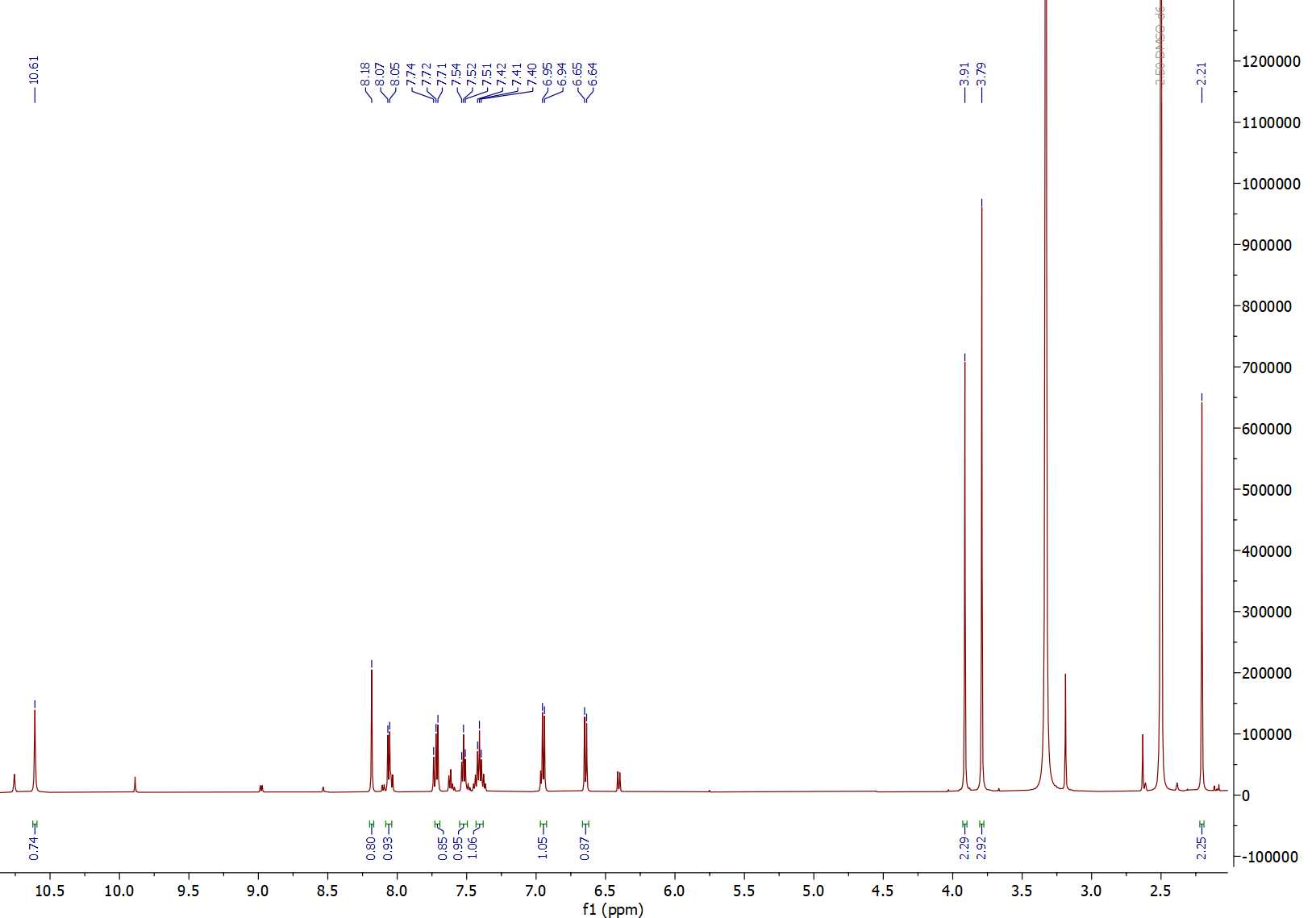


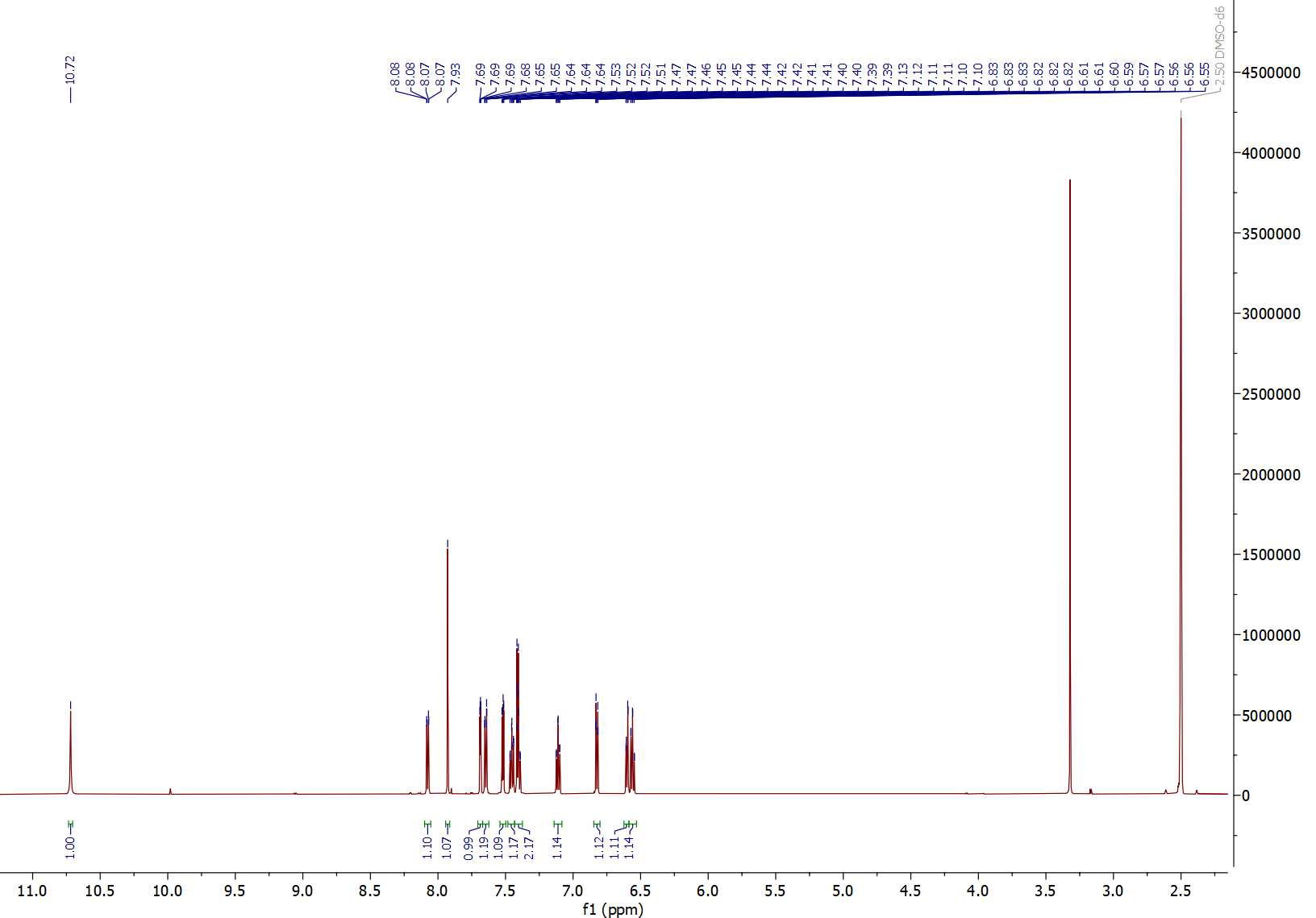

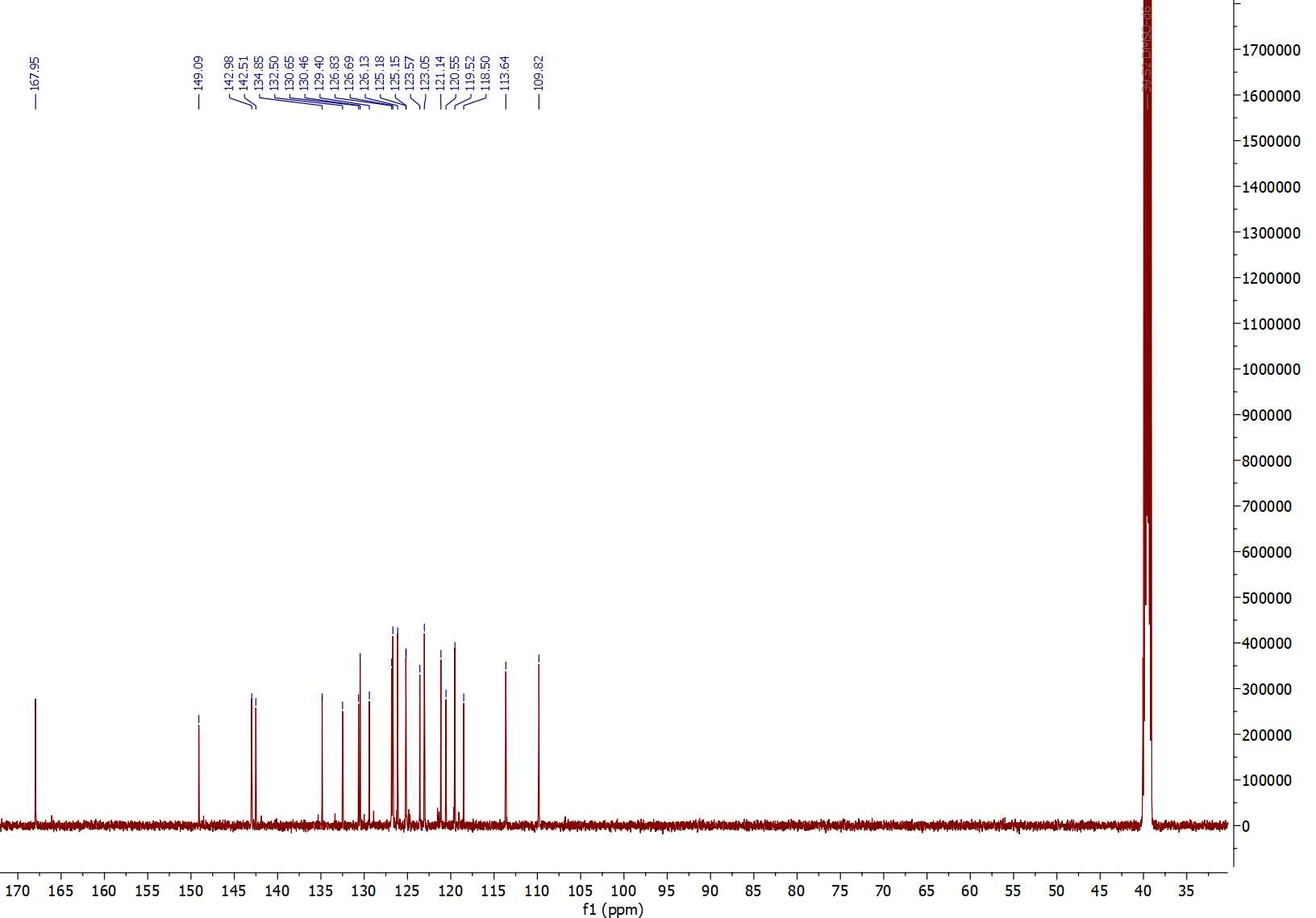


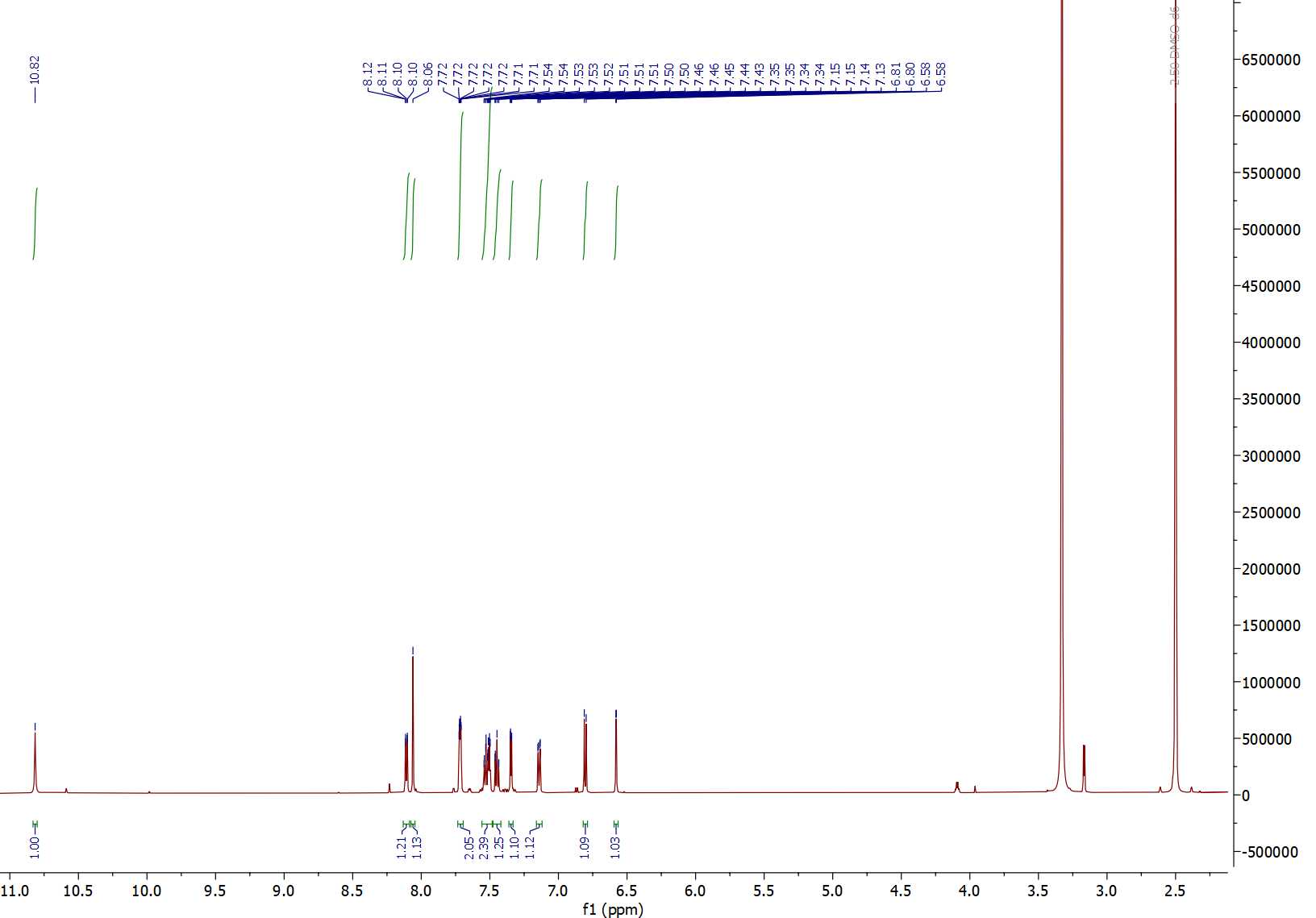


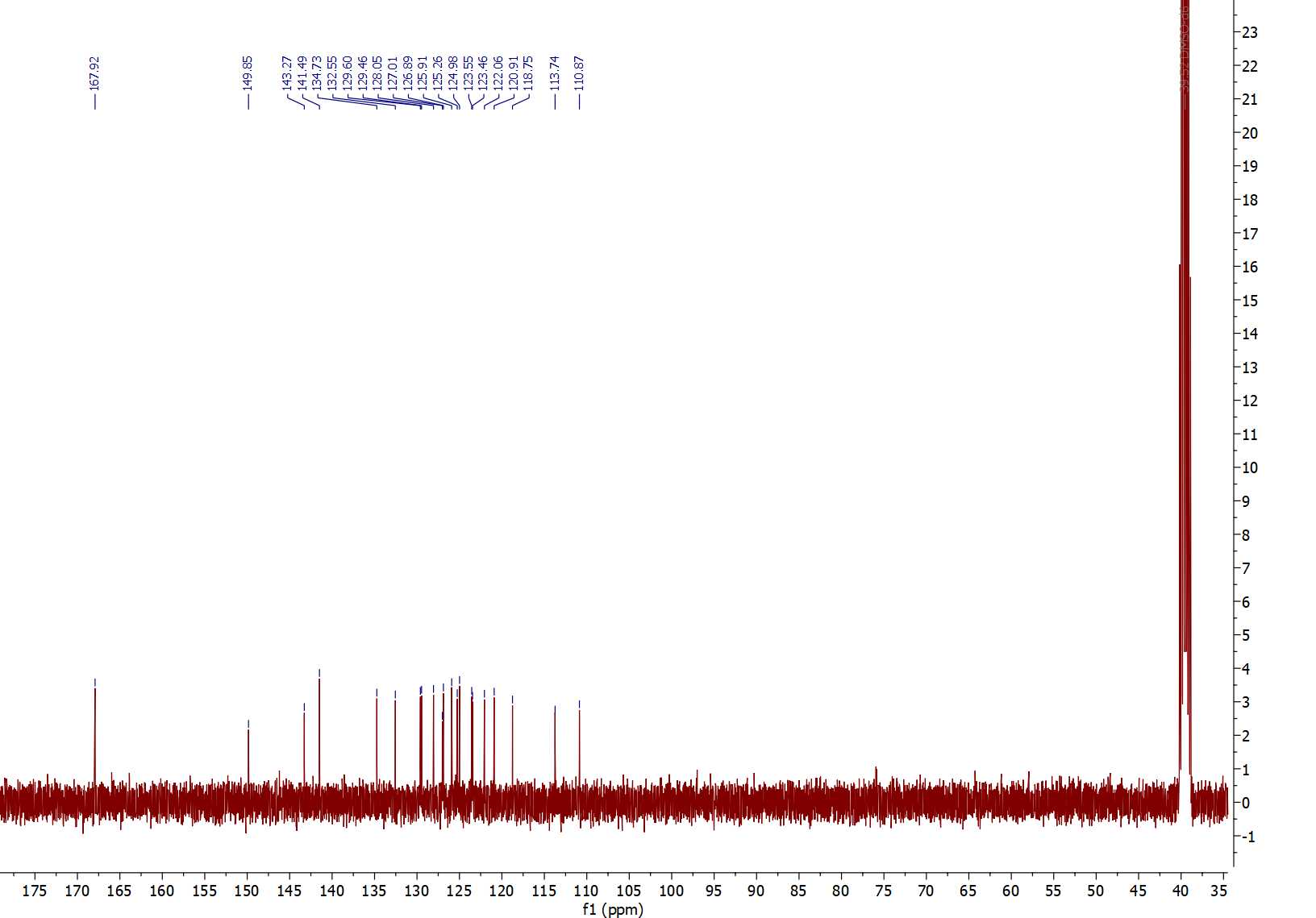


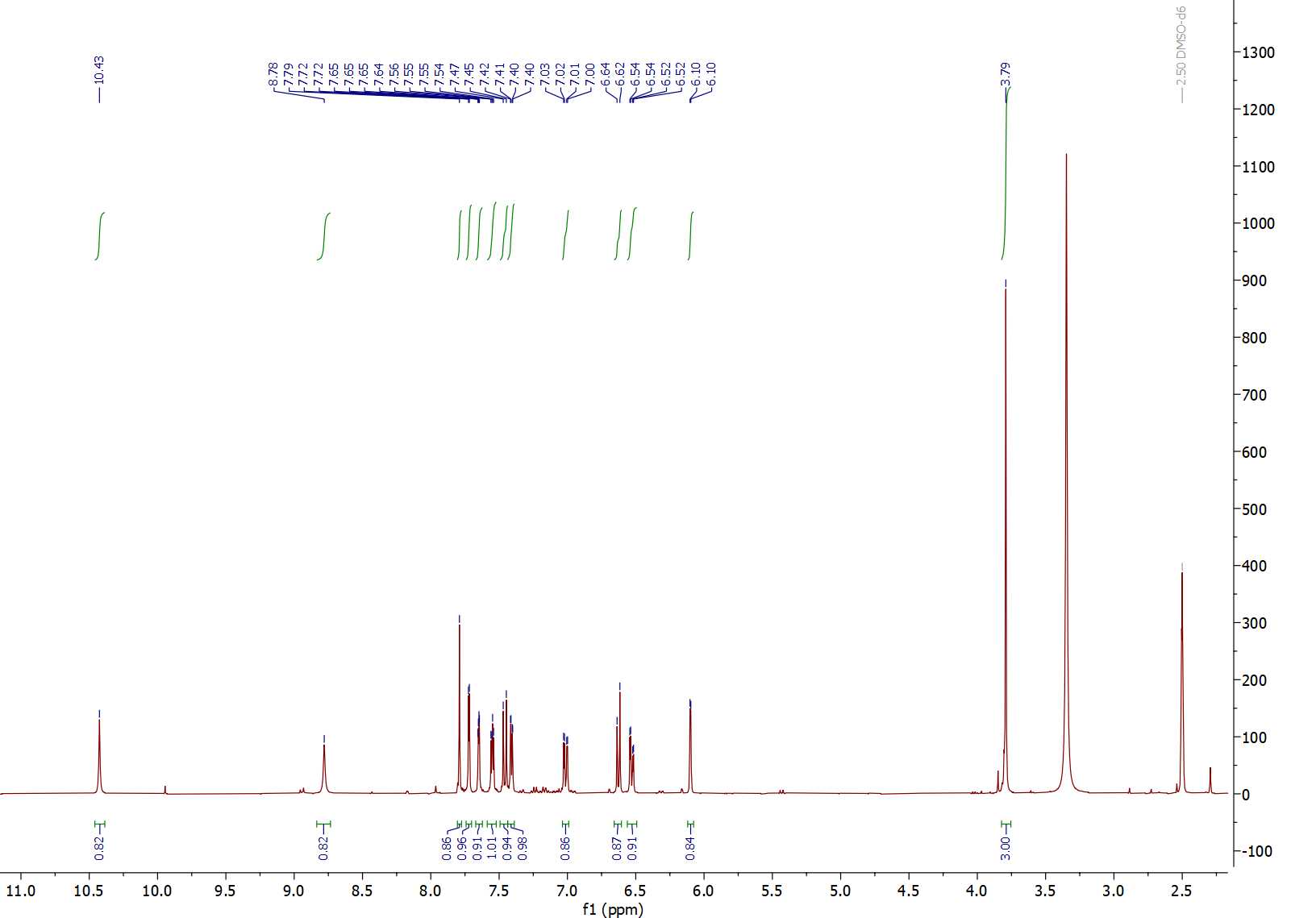


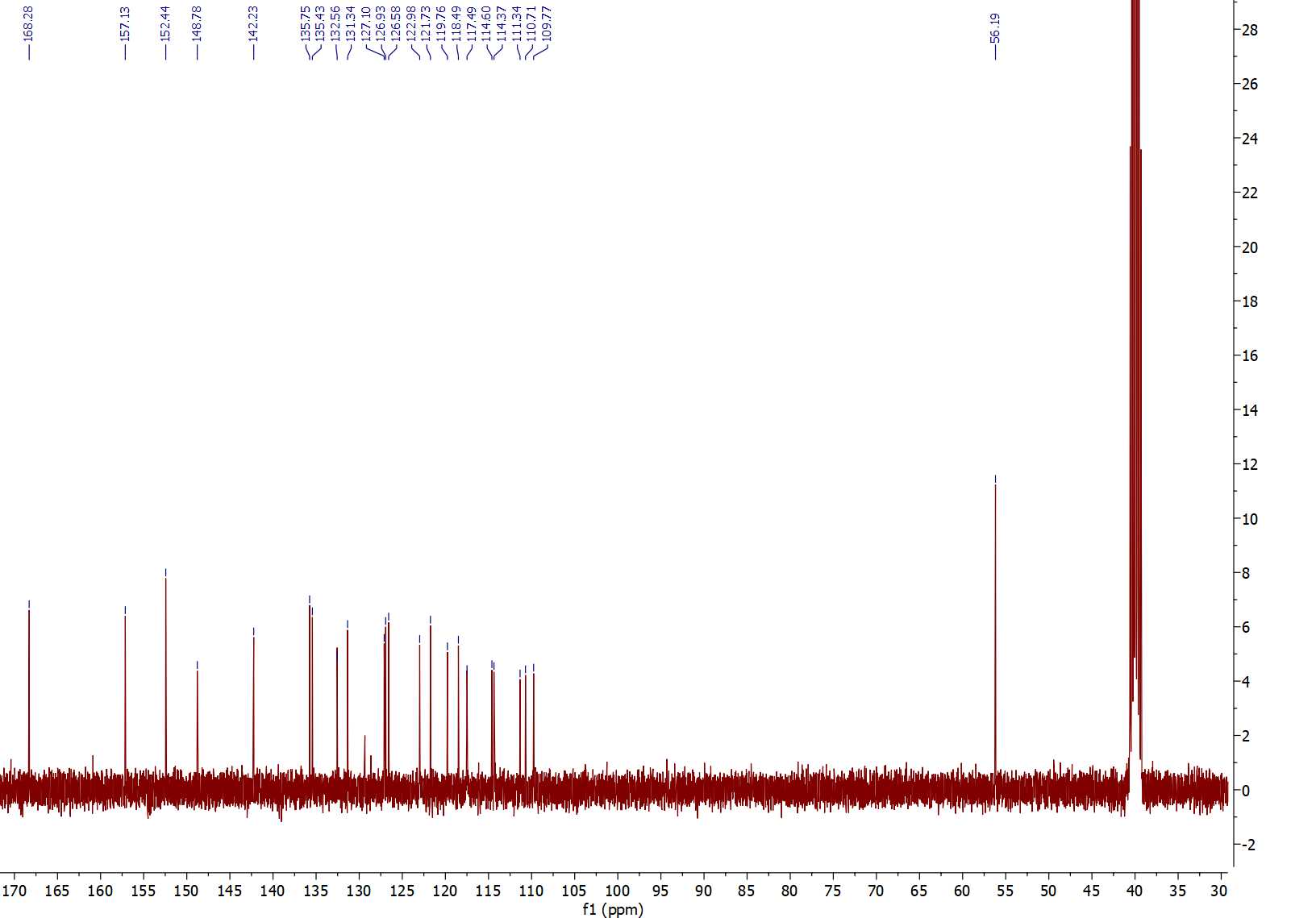


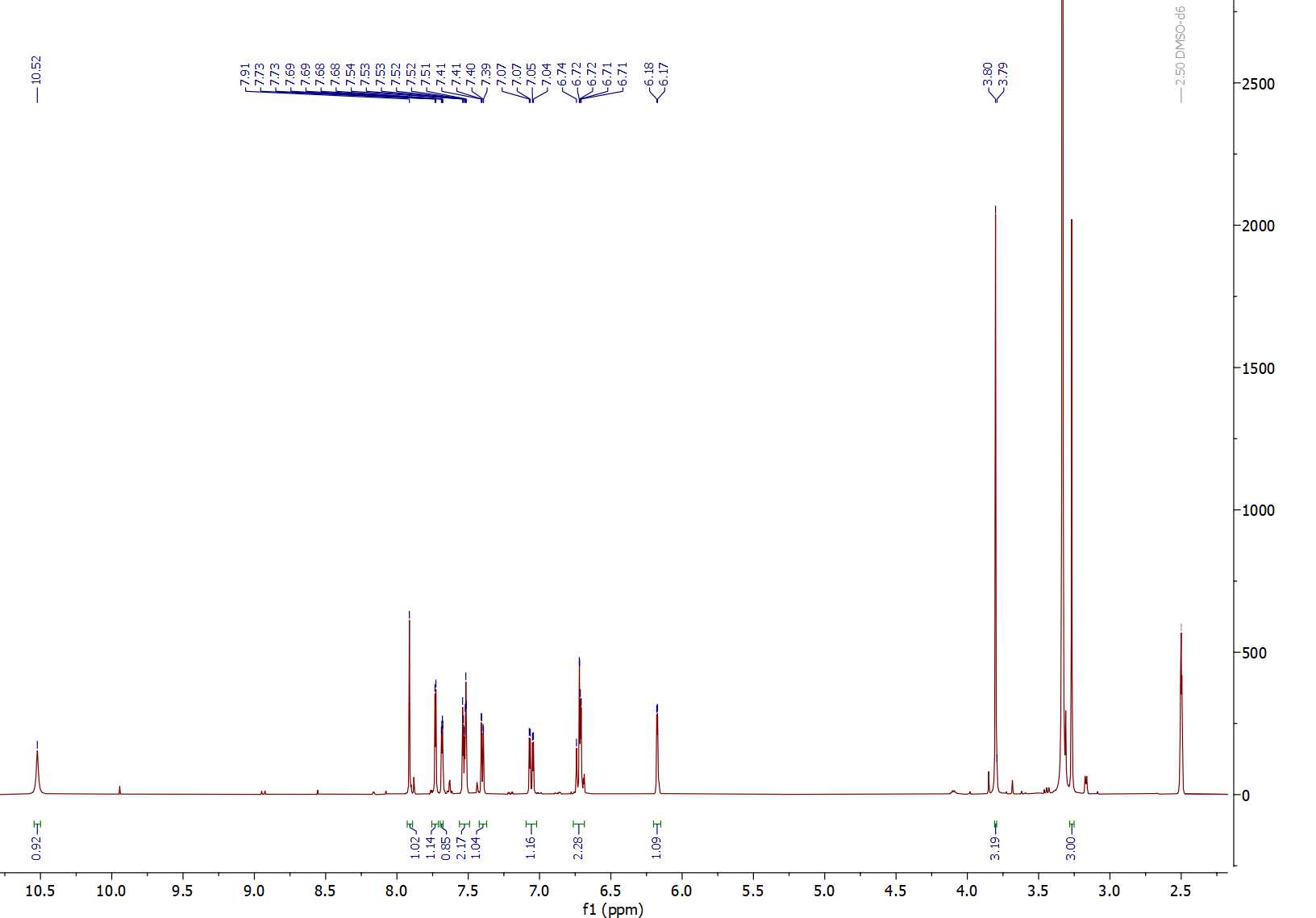


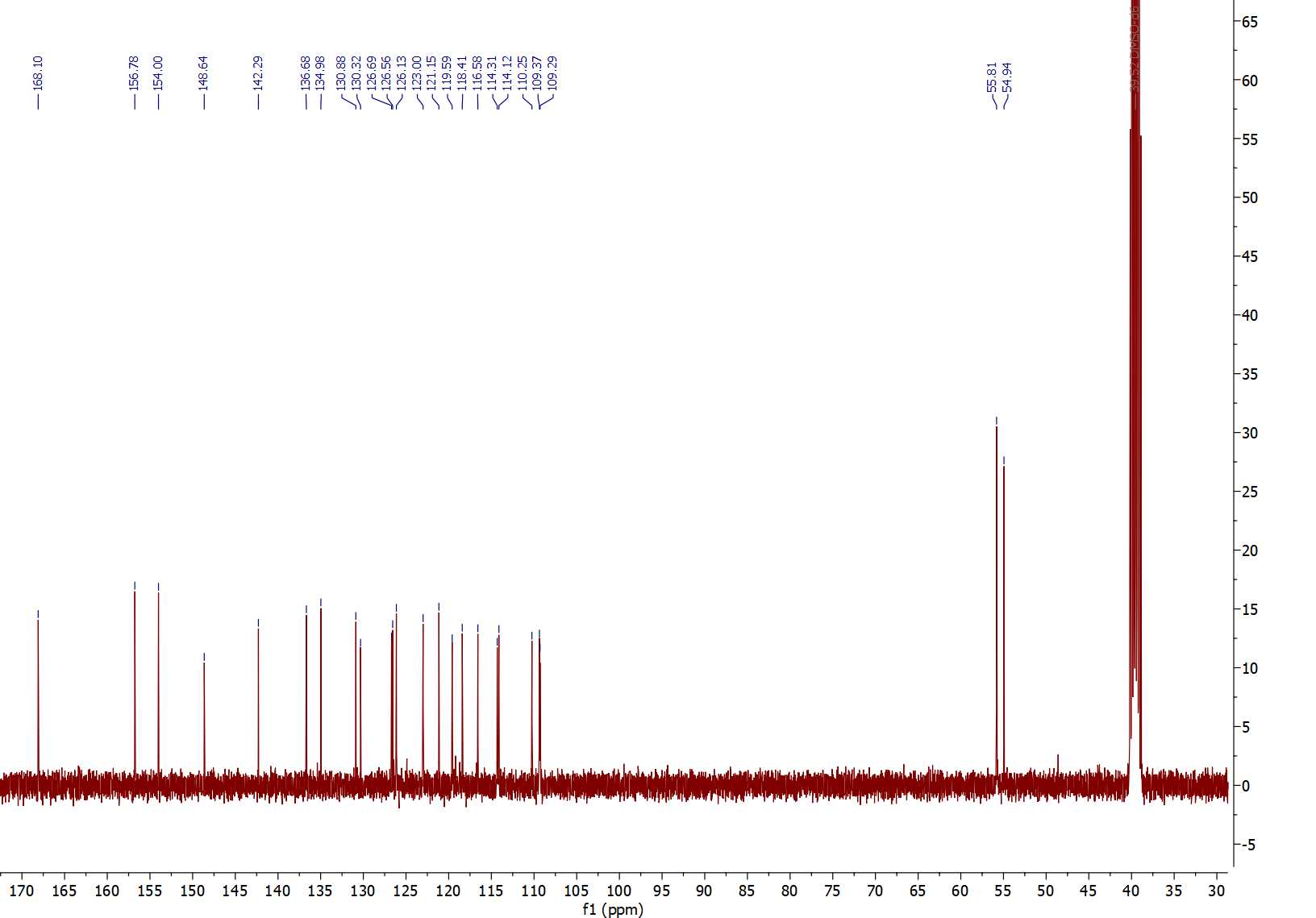


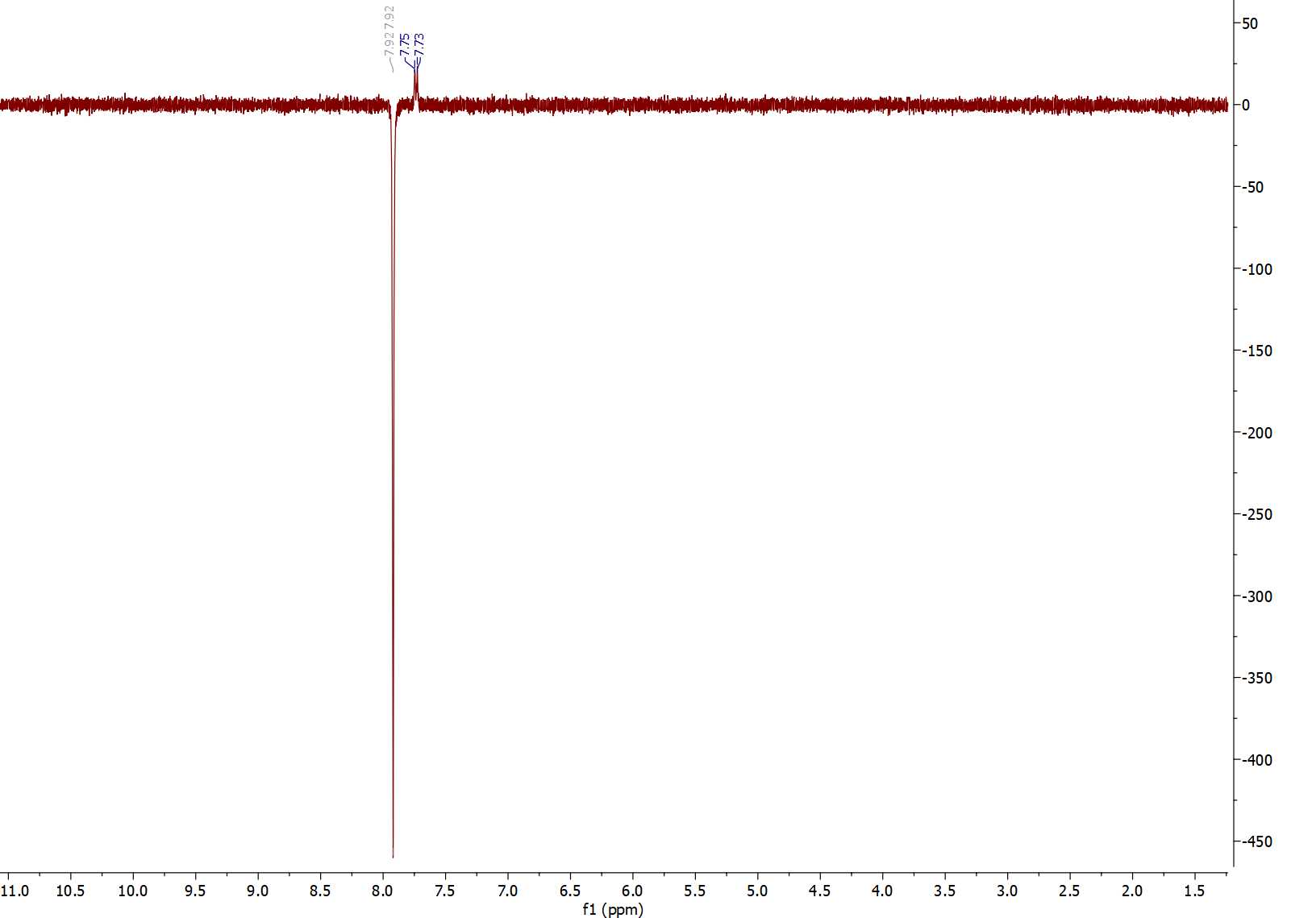


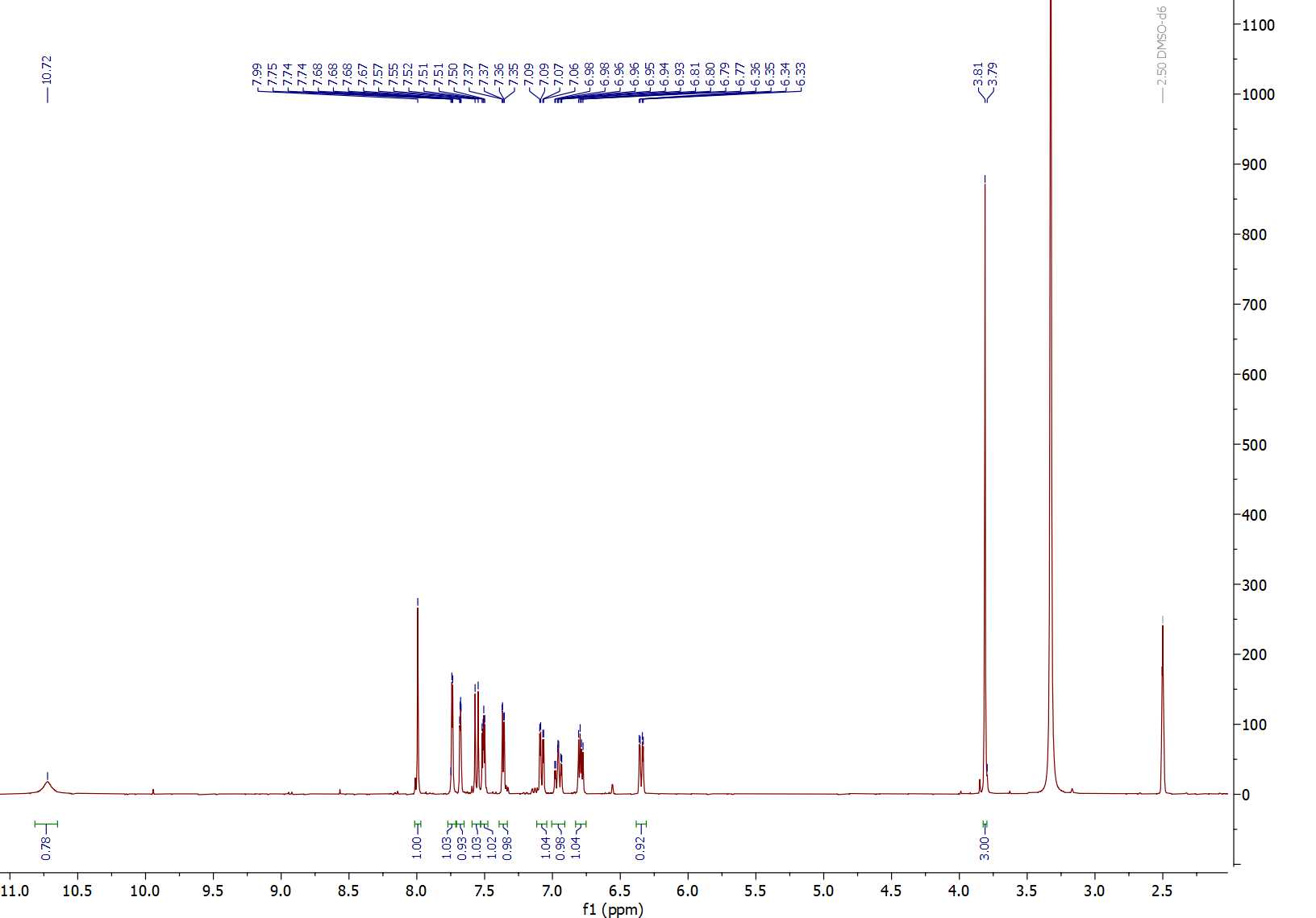


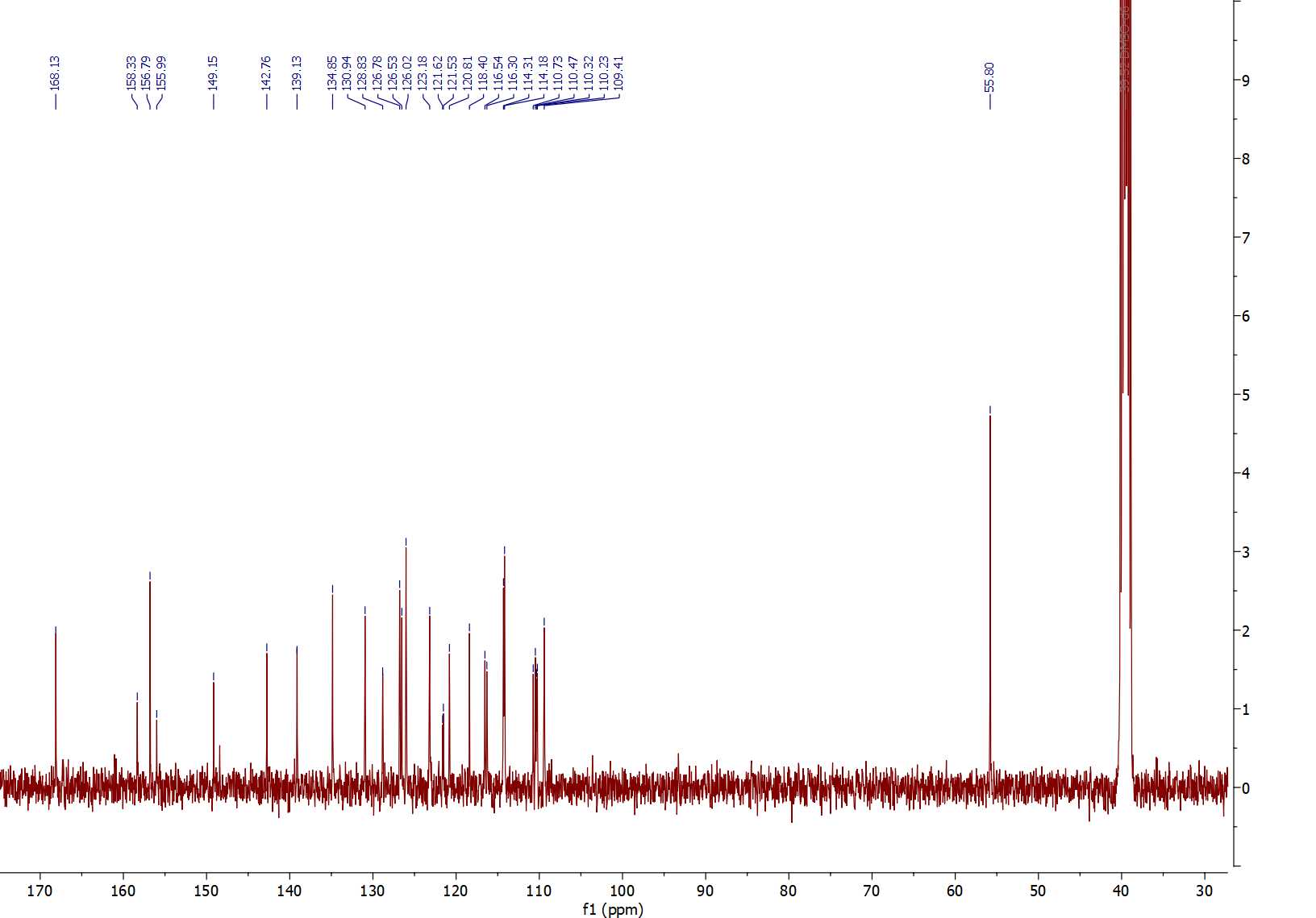


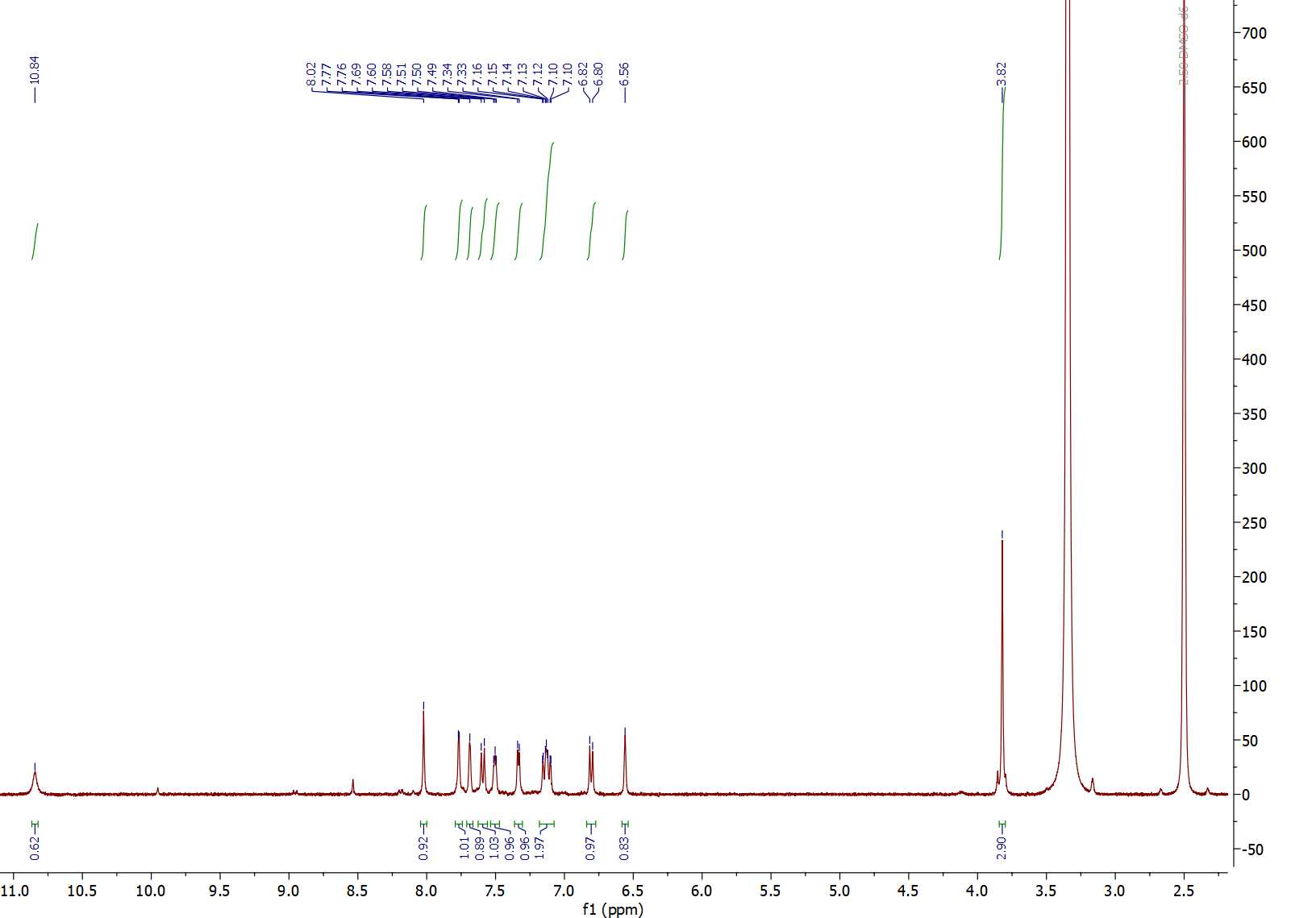


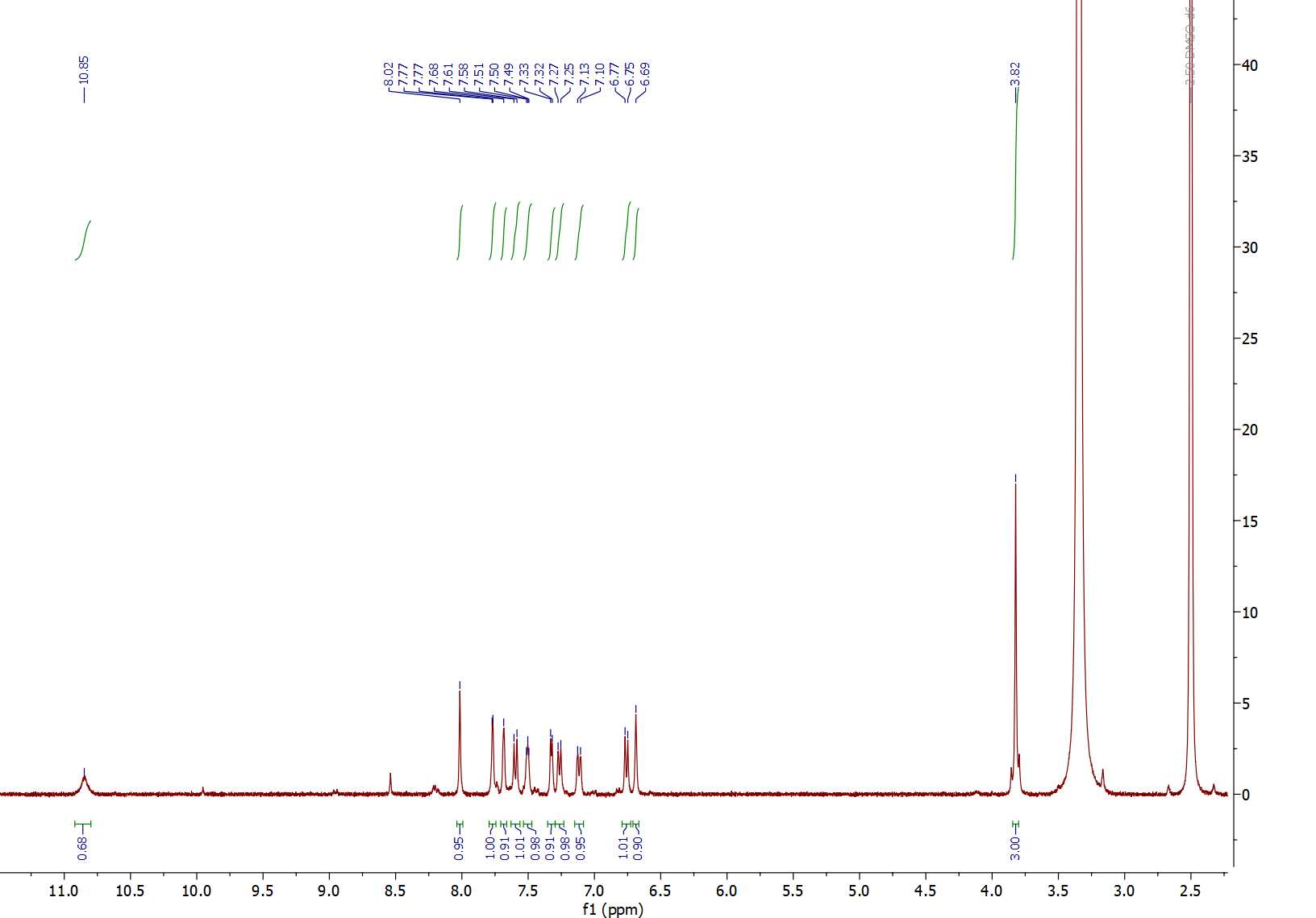


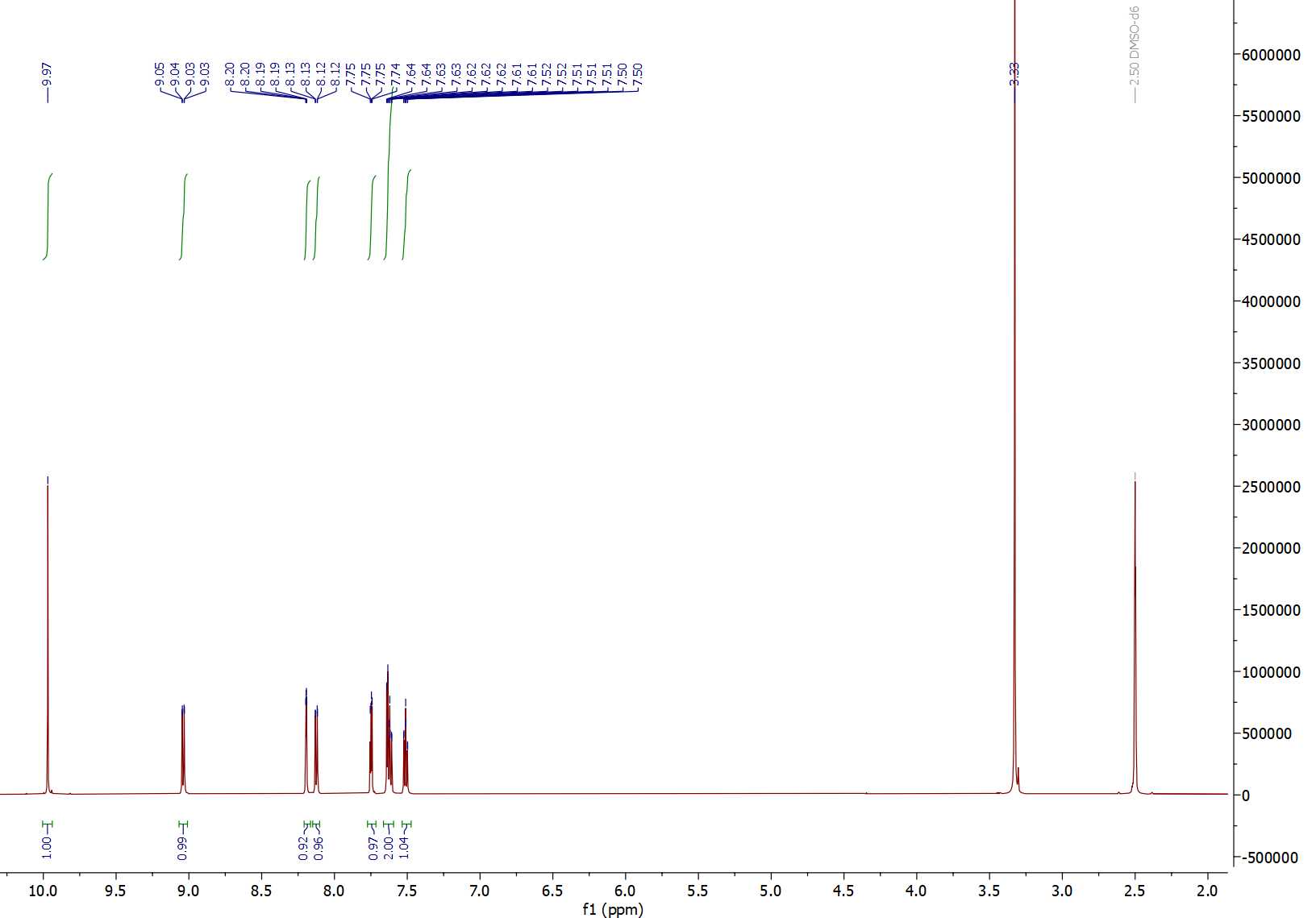

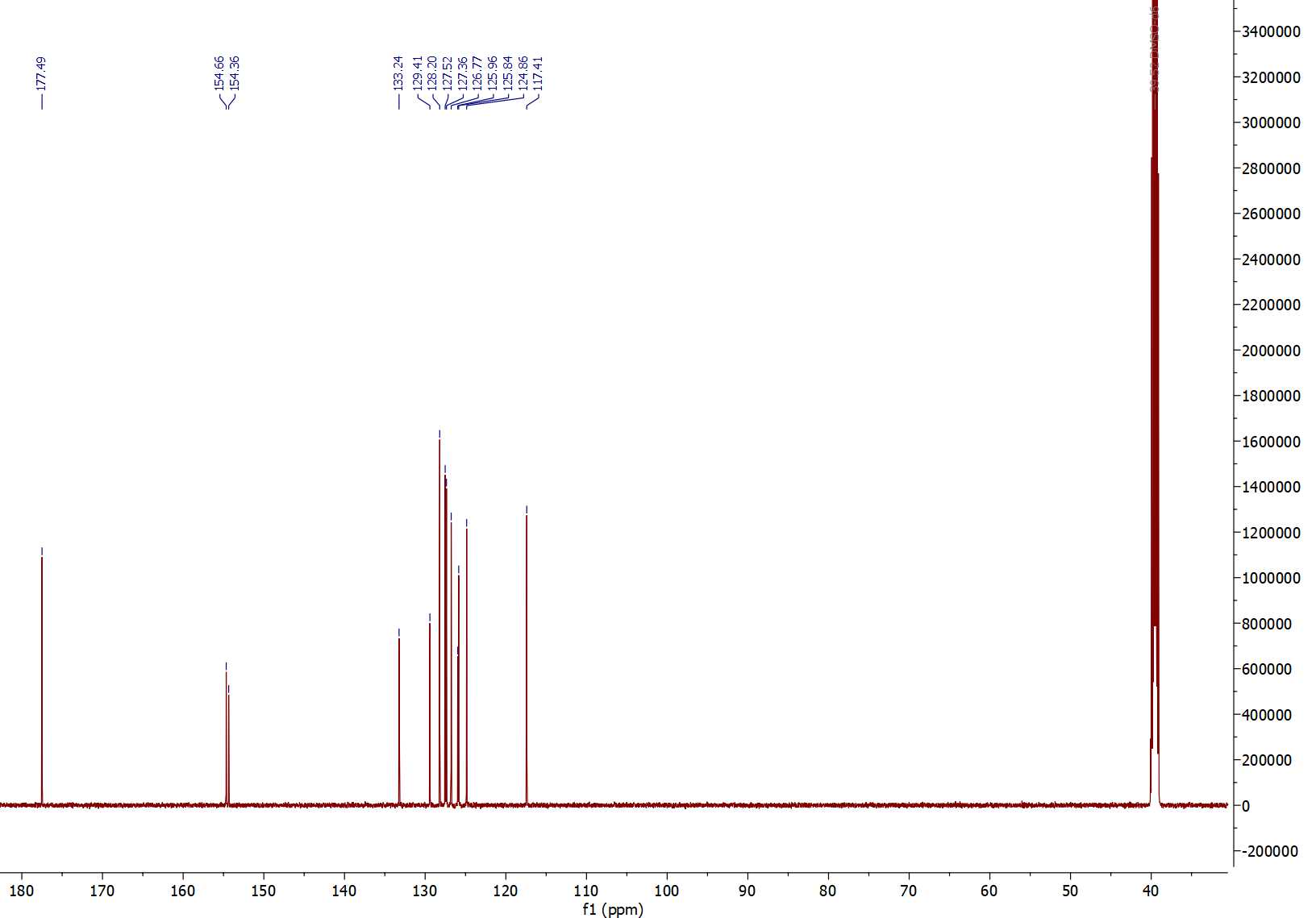

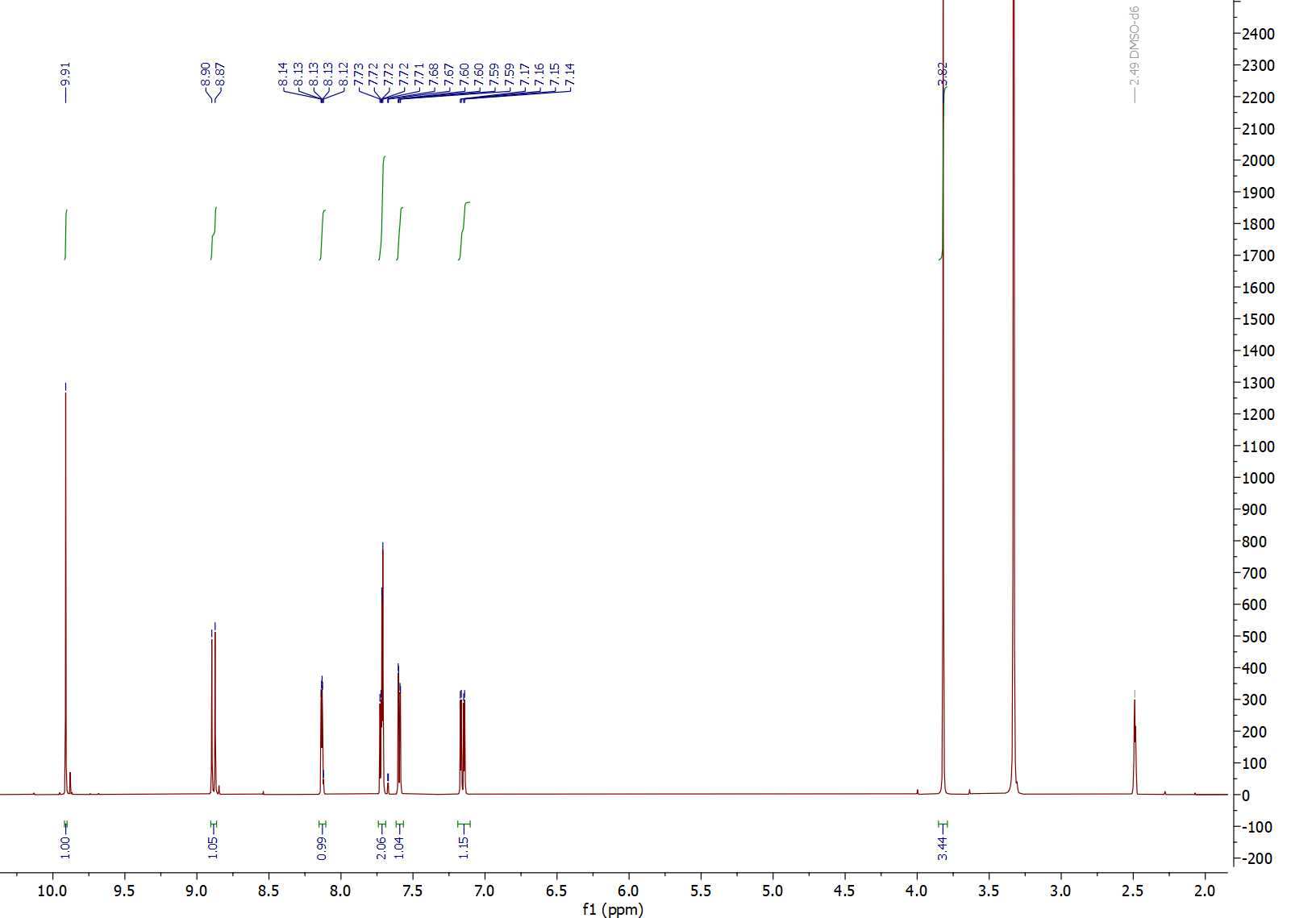

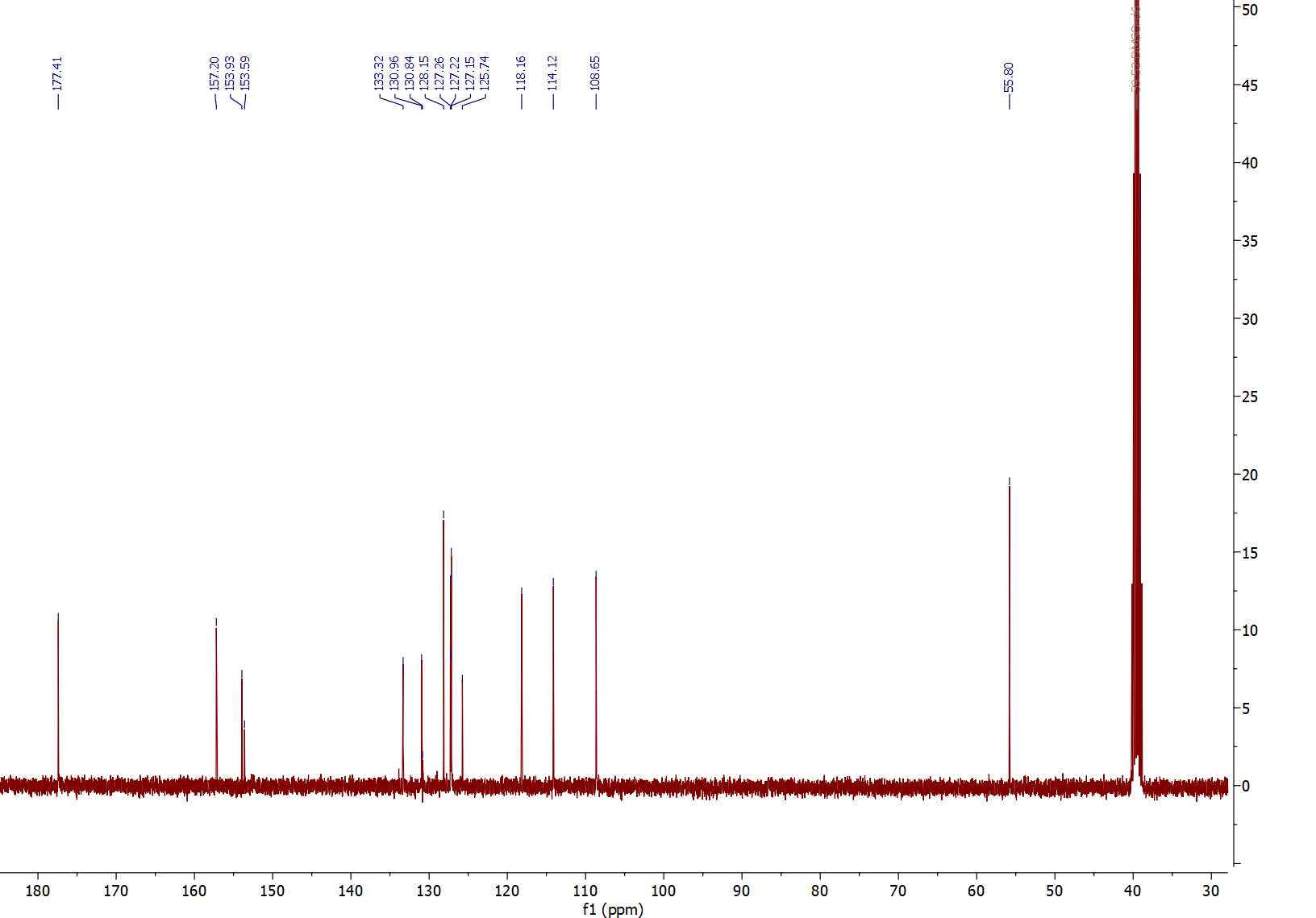

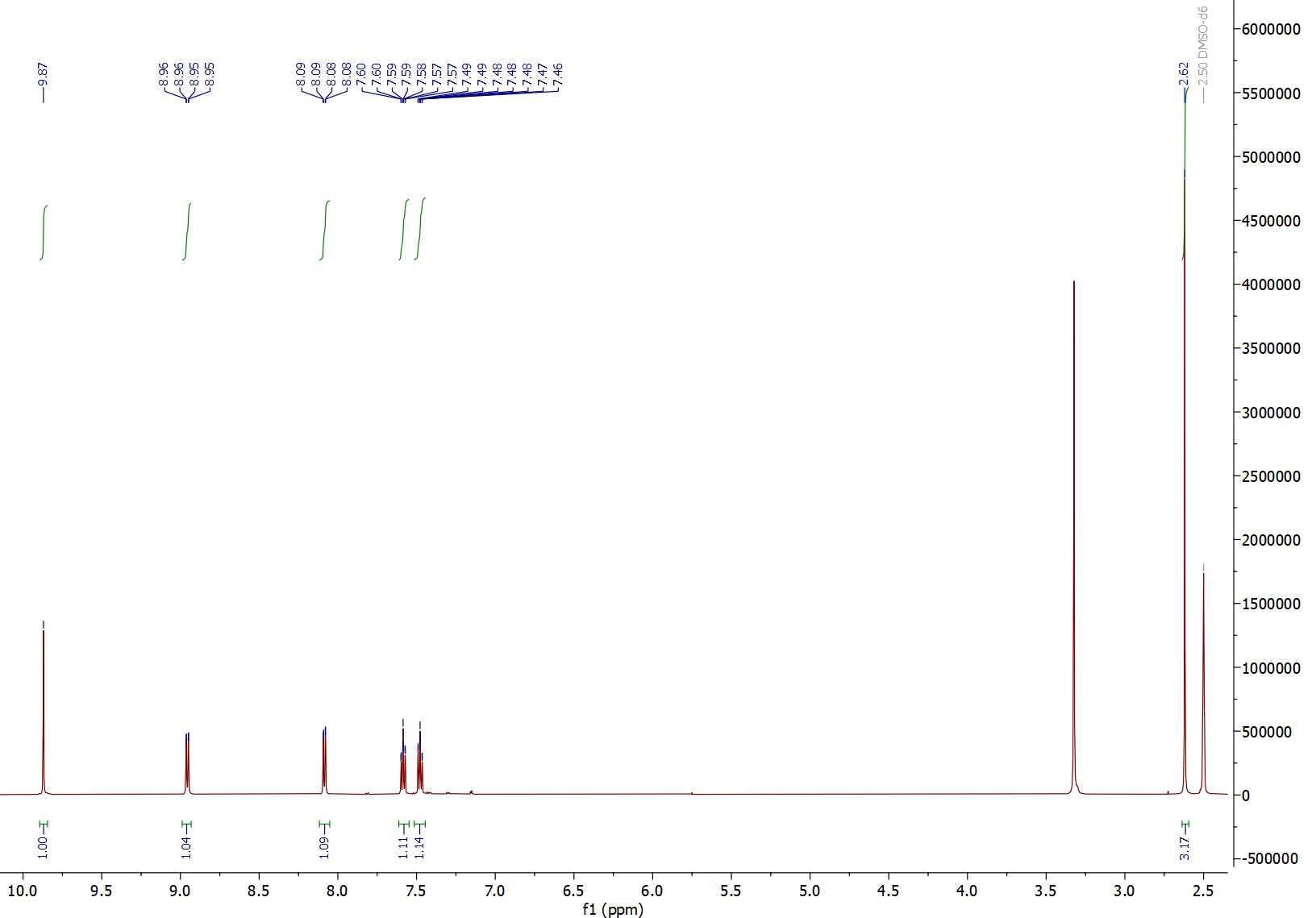


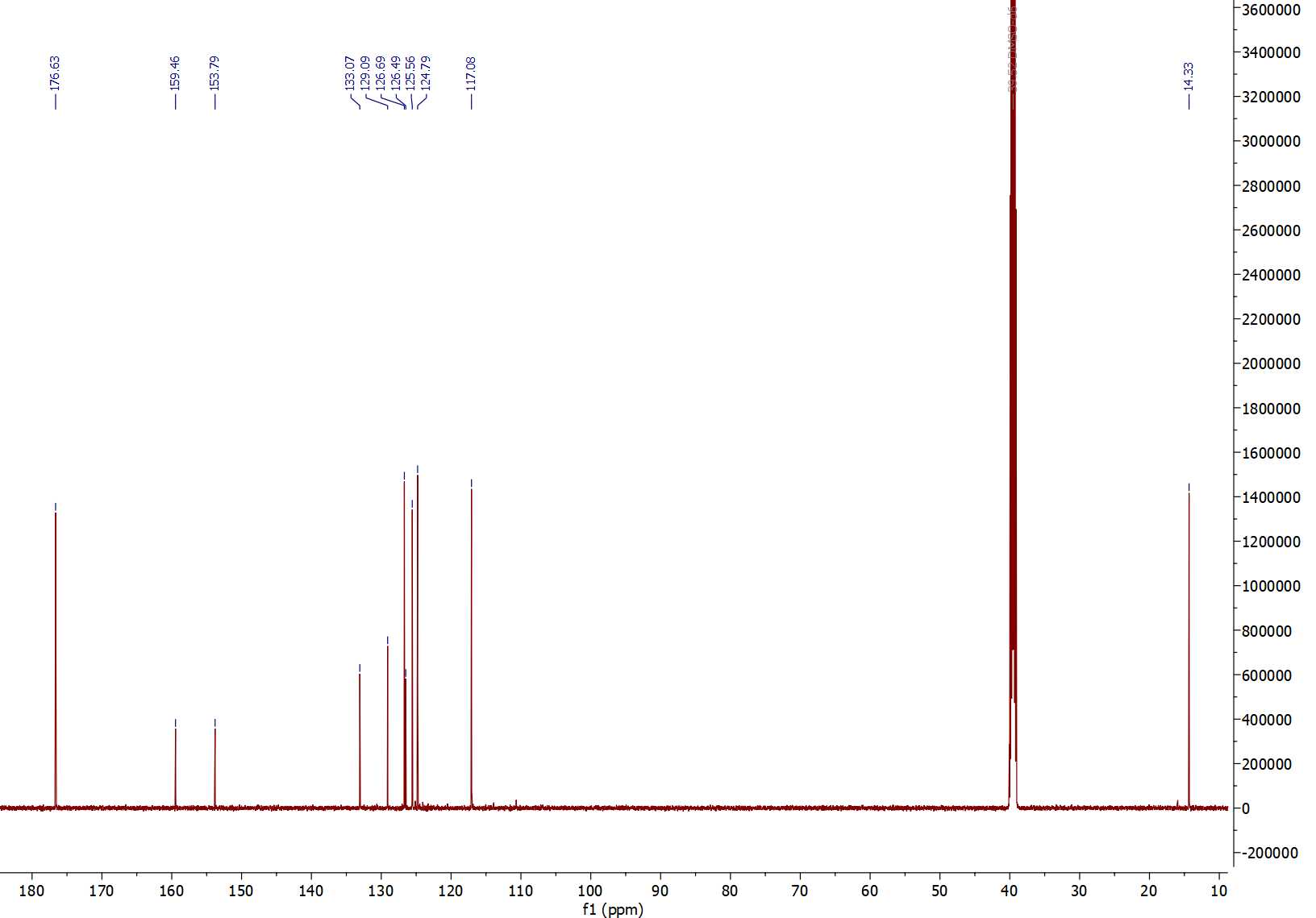


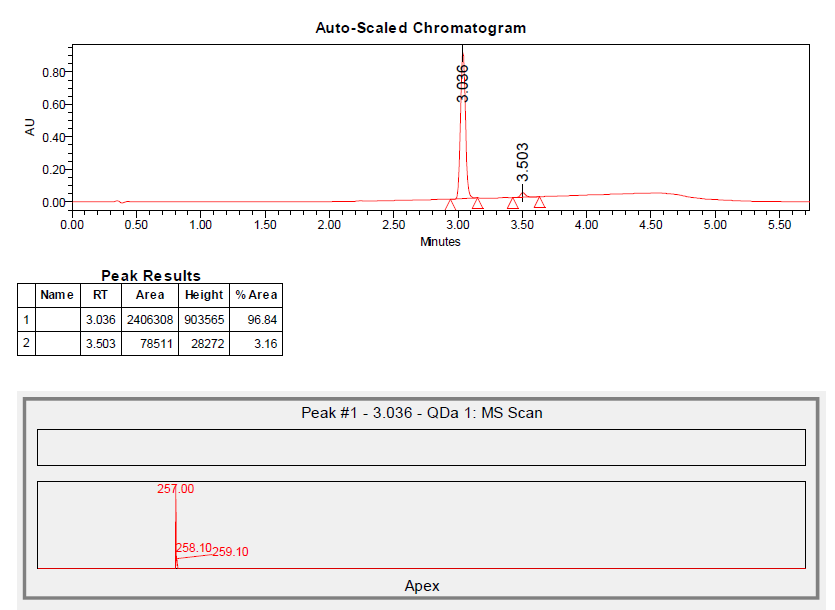


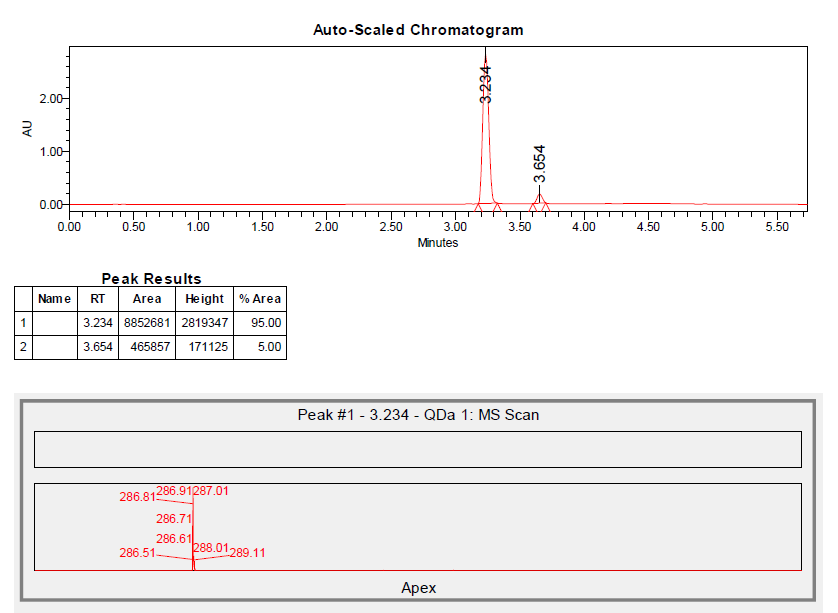


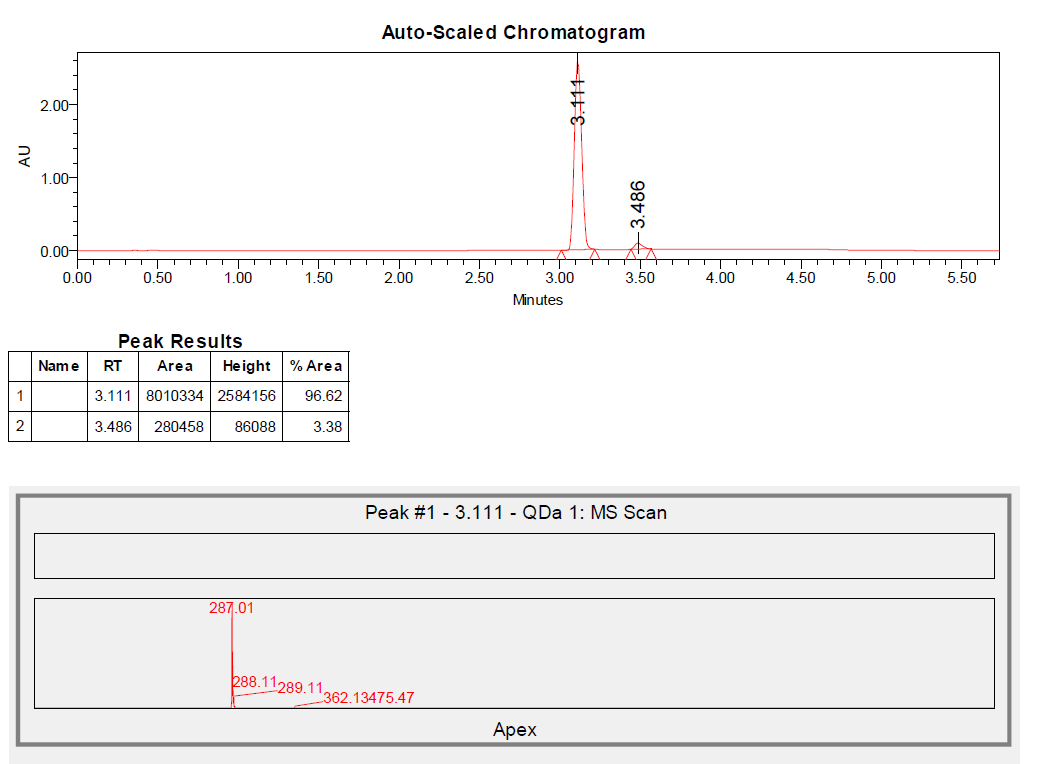


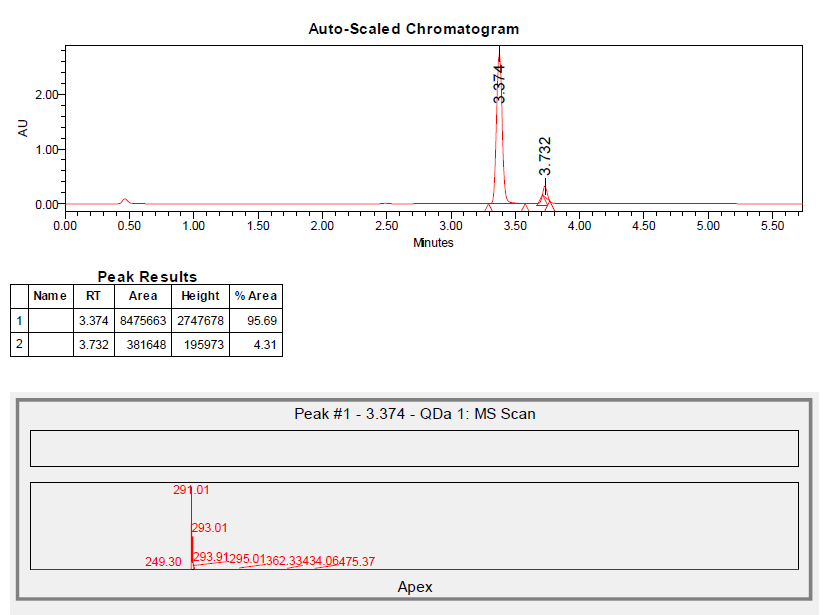


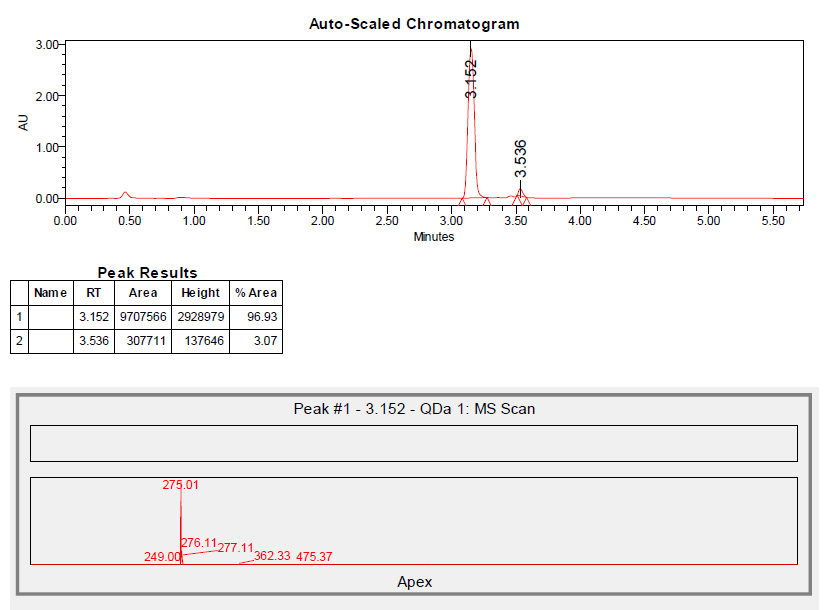


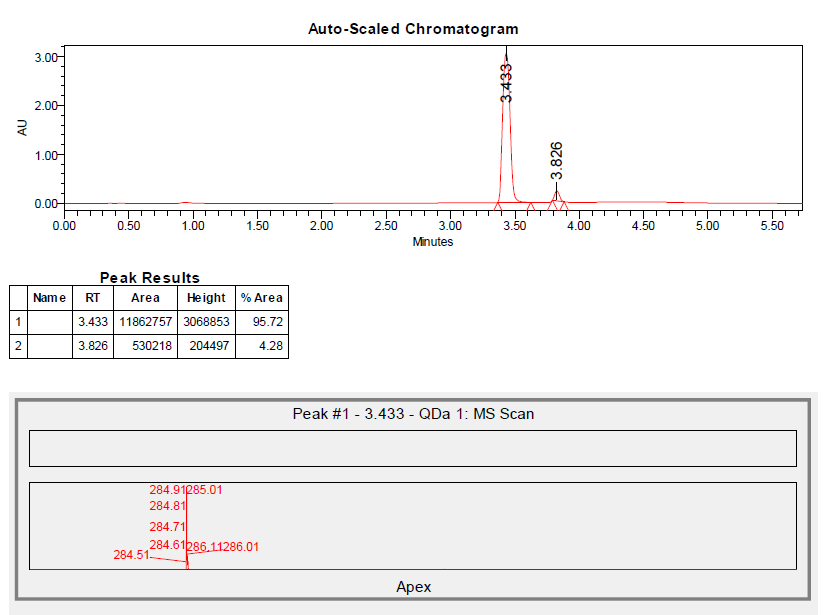


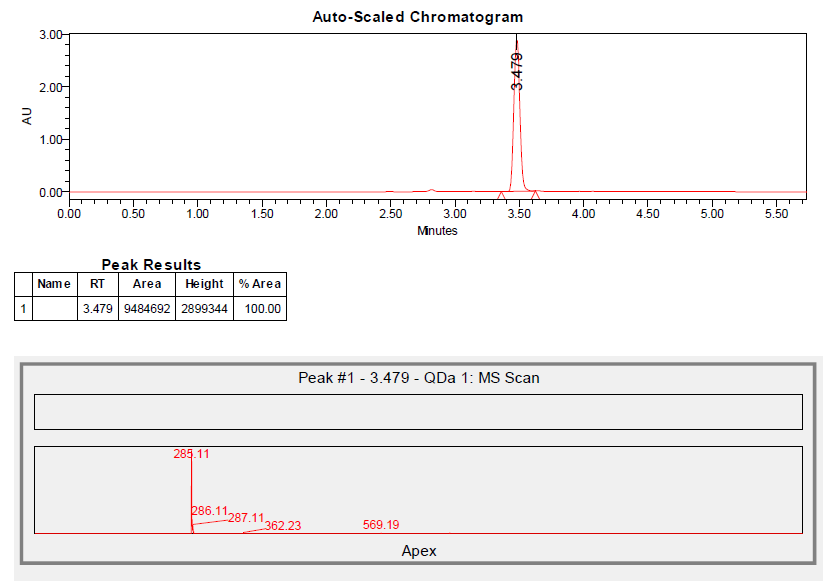


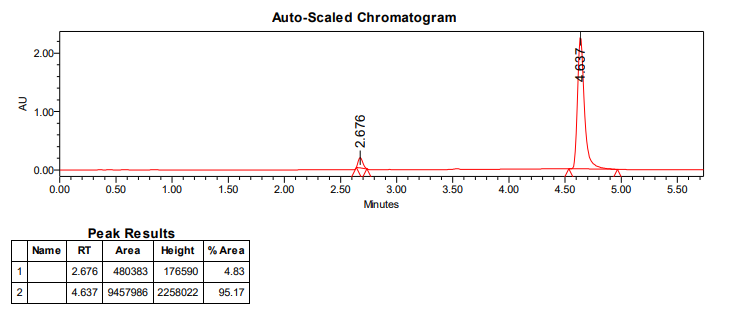


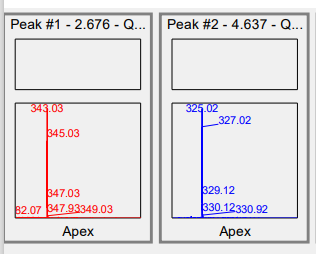


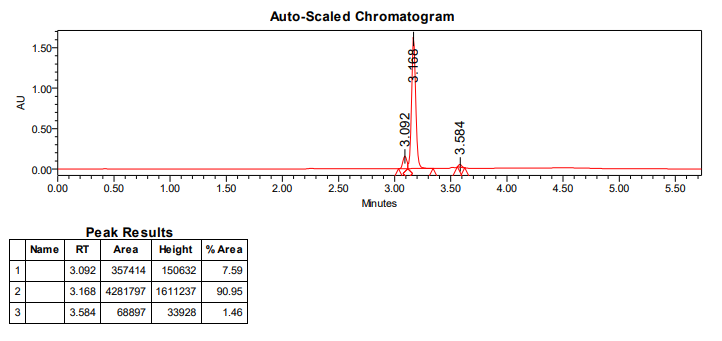


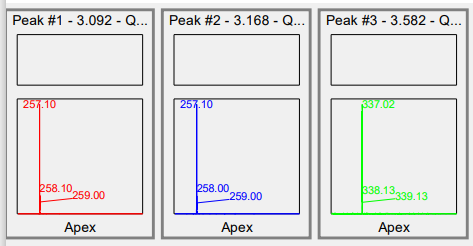


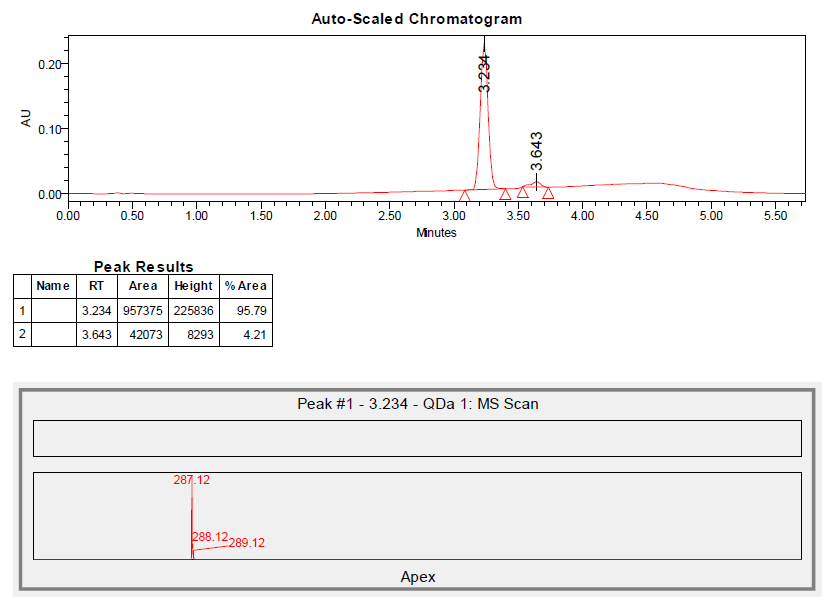


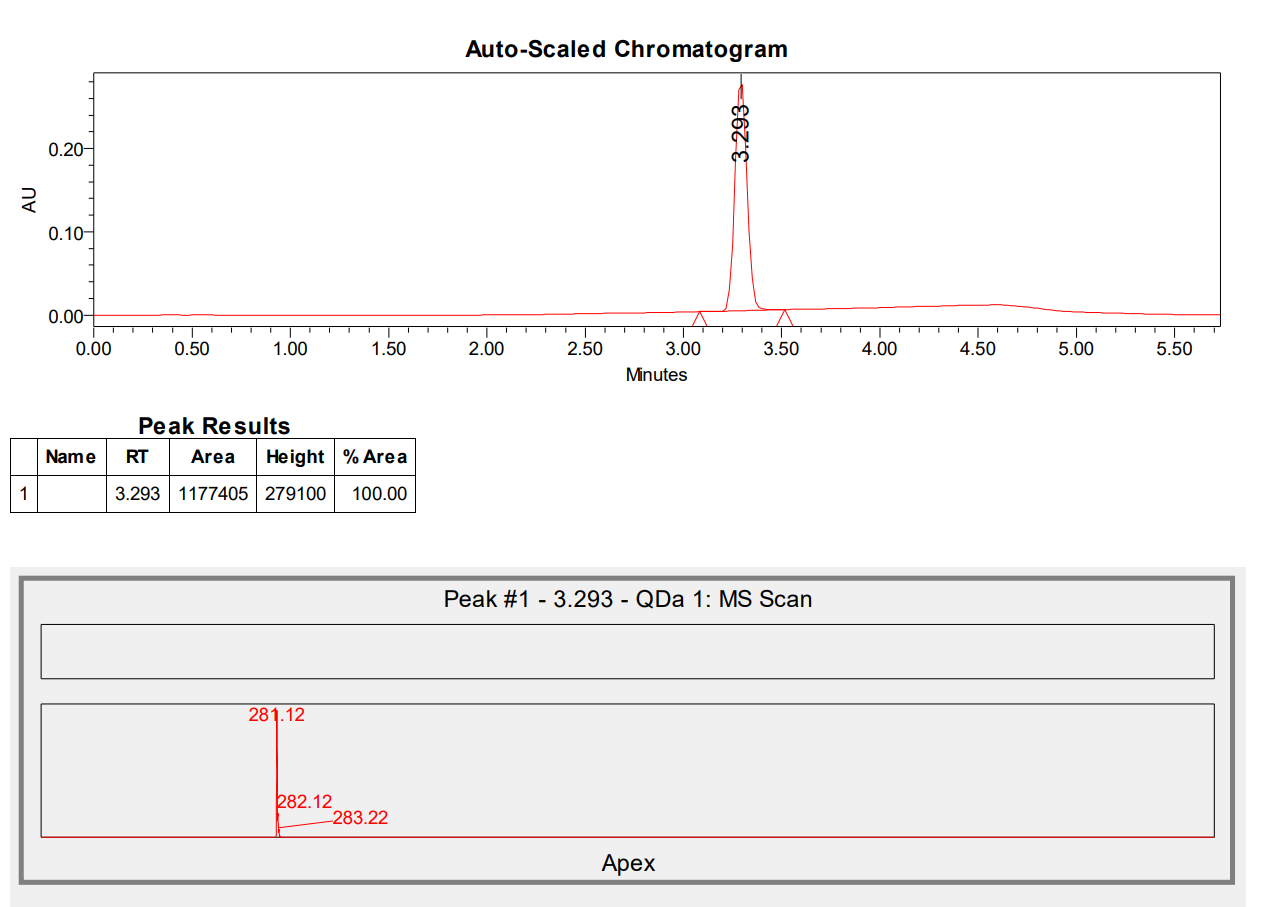


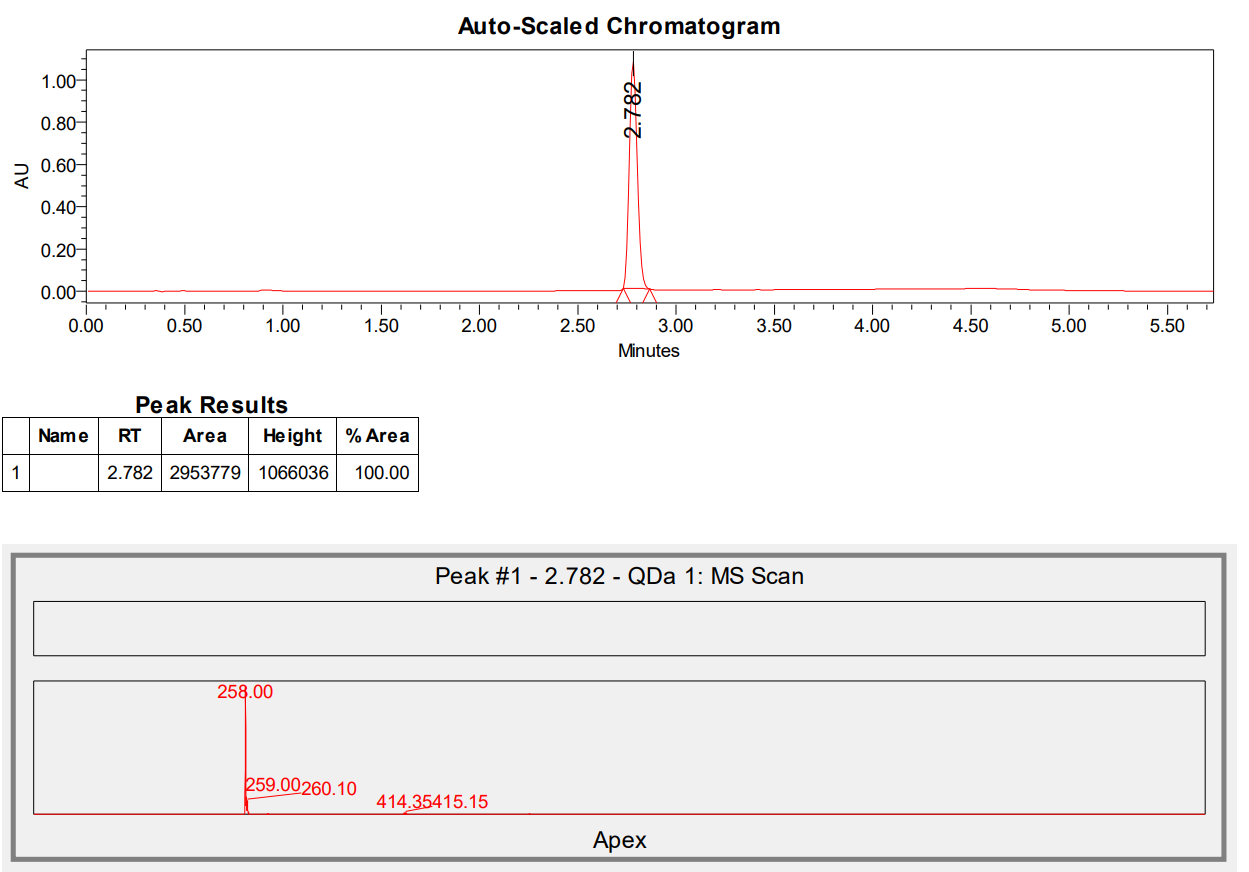


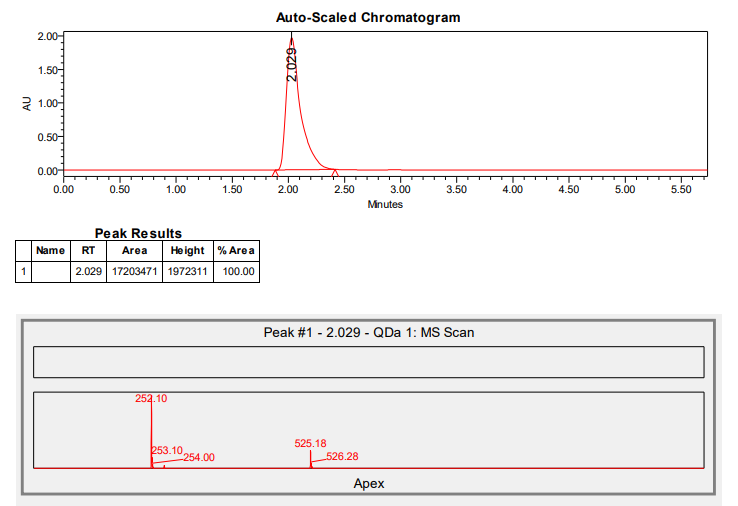


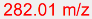

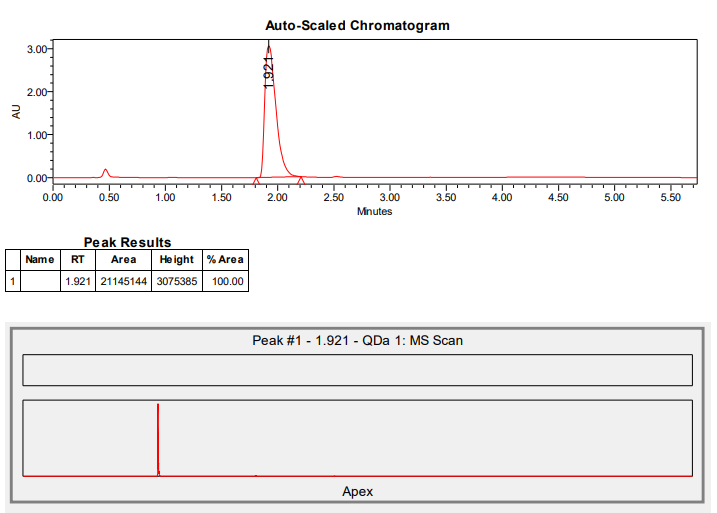


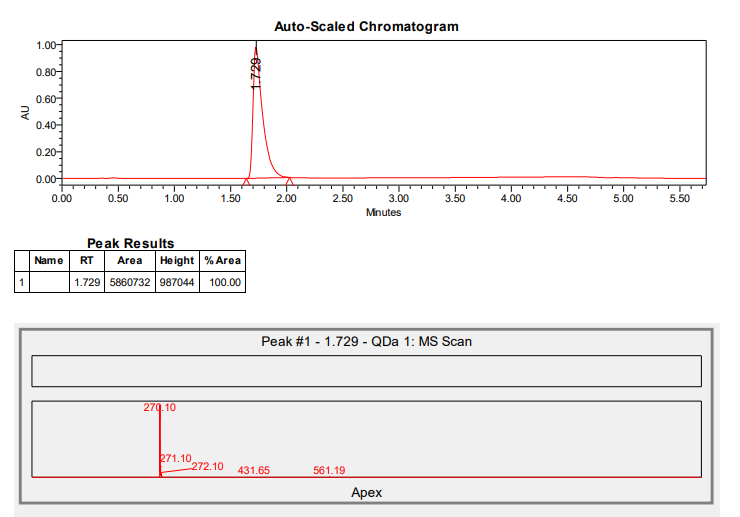


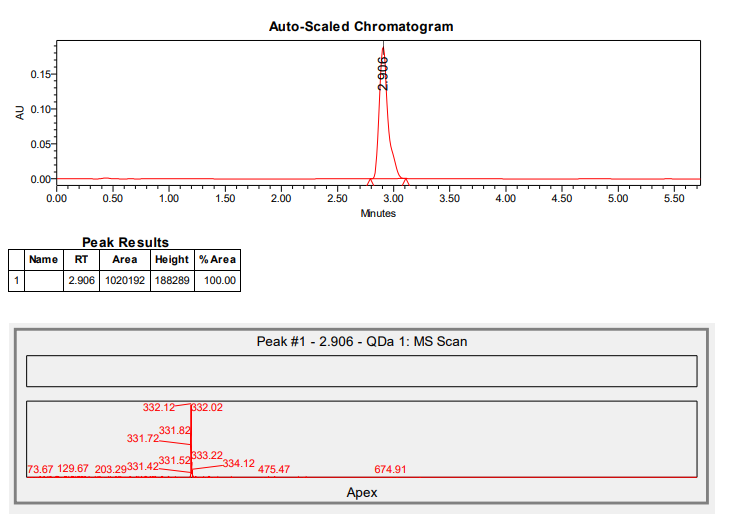


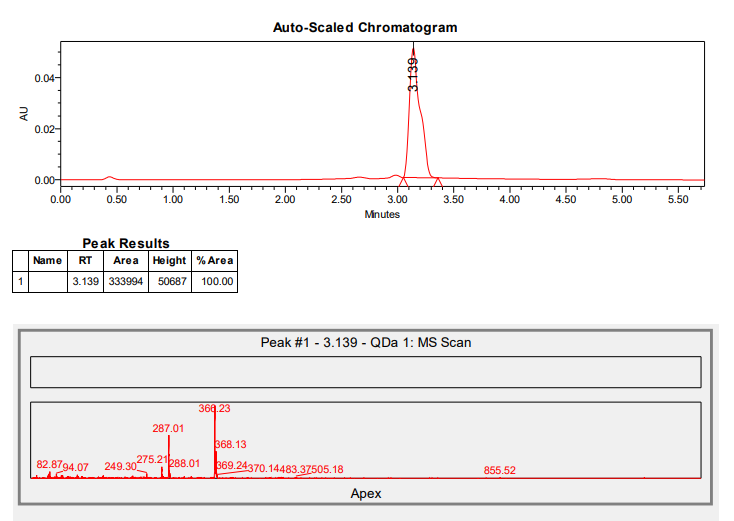


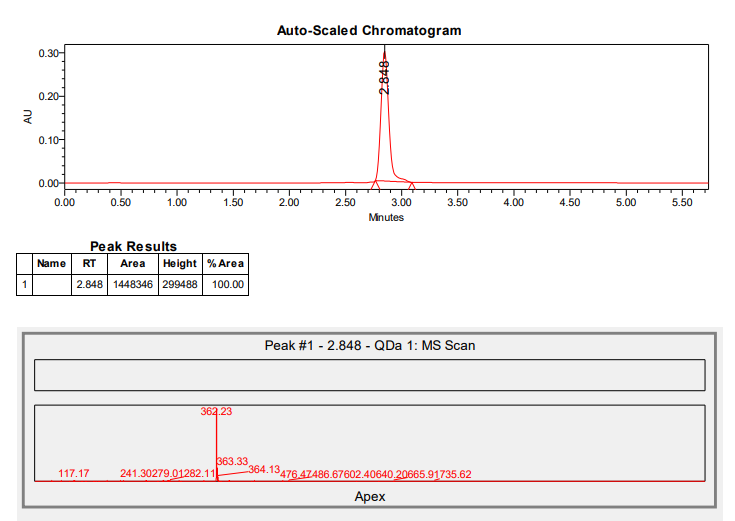


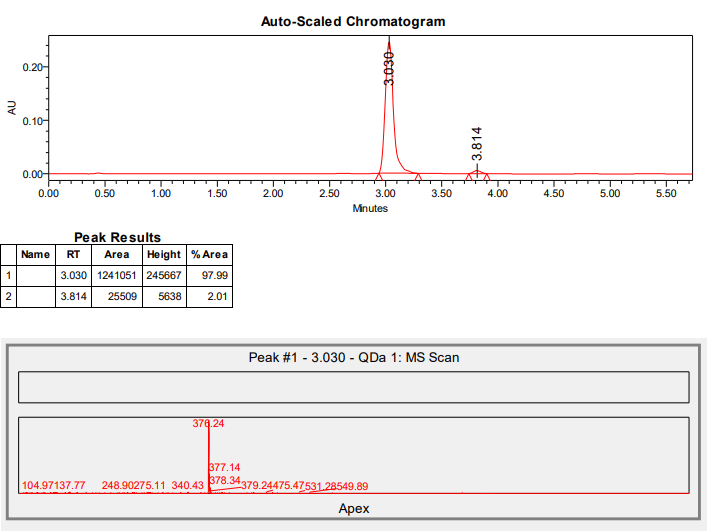


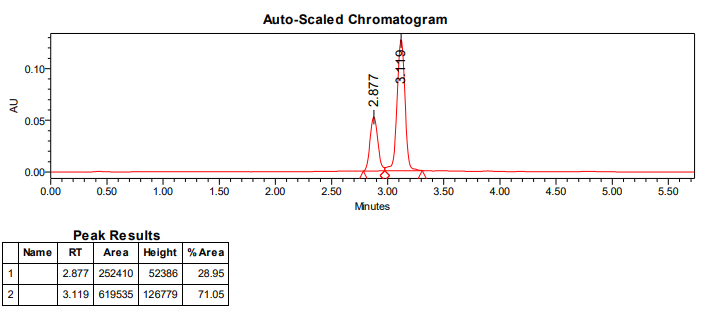


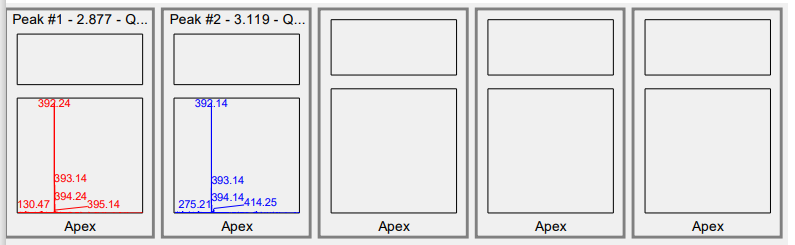


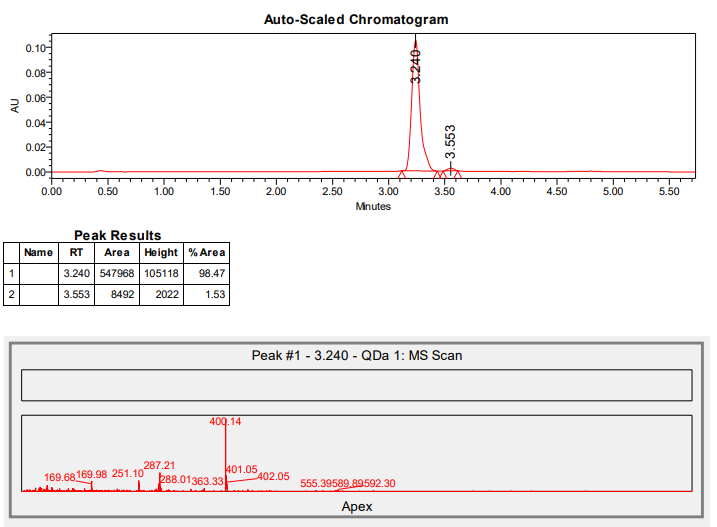

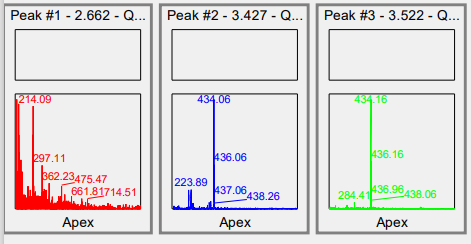


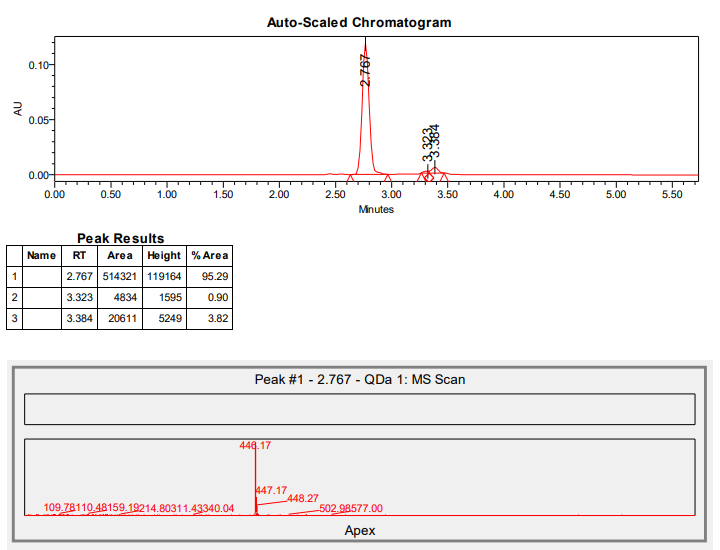


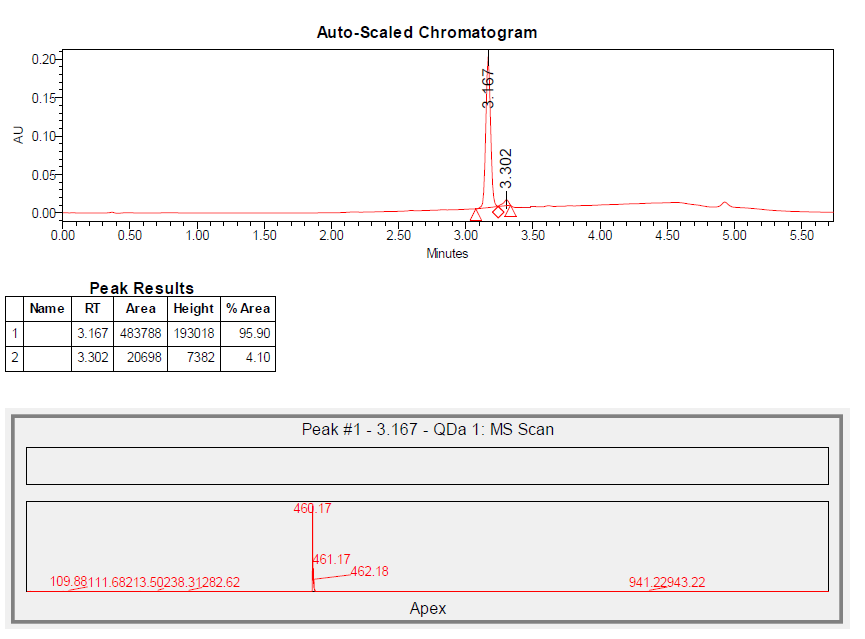


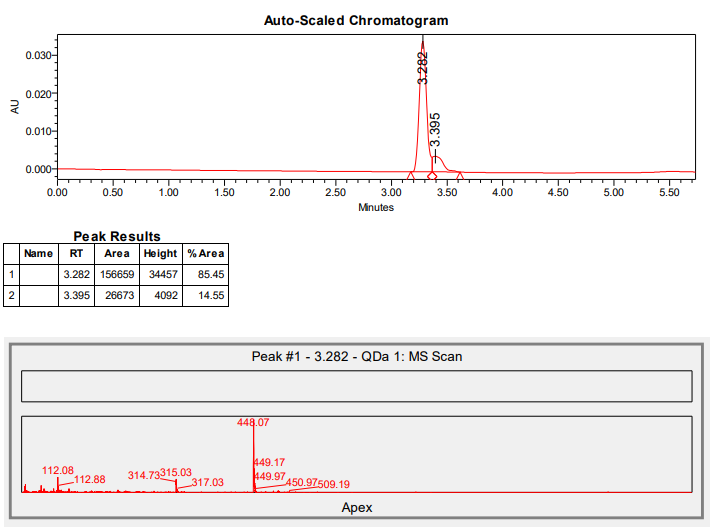


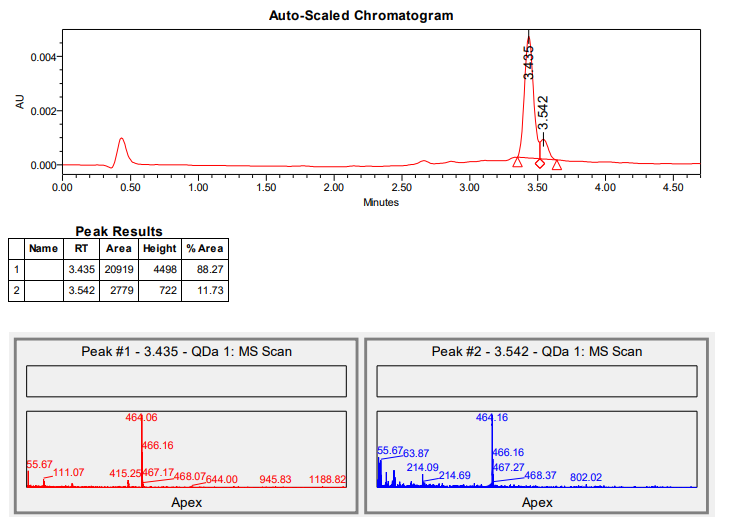


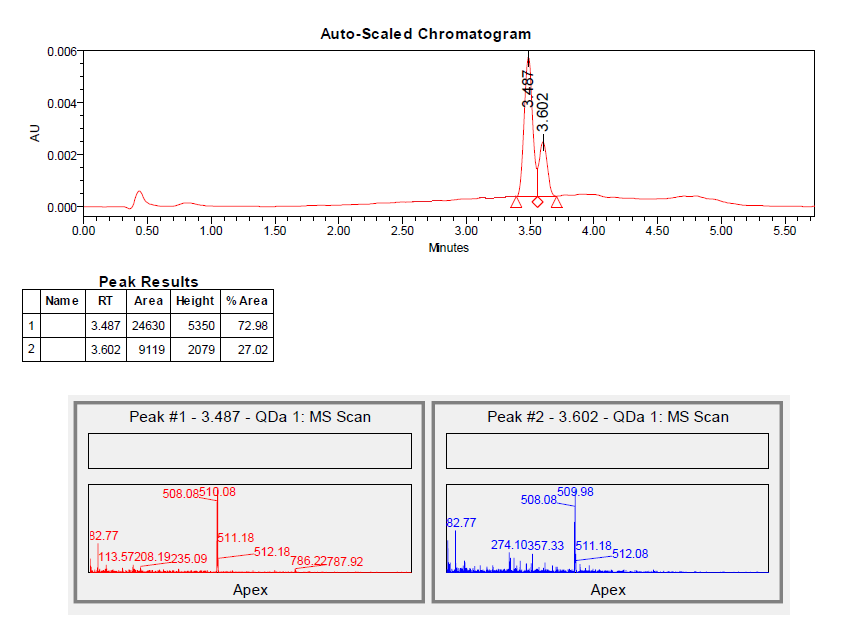


Please note that compounds **20**, **22**, **25**-**27** undergo geometrical isomerization when injected for UHPLC-MS analysis, therefore giving two chromatographic peaks showing a mass spectrum with the same m/z value. This happens because the eluent contains formic acid, that is necessary for the ionization of the molecules and subsequent obtainment of mass spectra. We also tried to use an eluent not containing acid, and no isomerization happened during the chromatographic run, but mass spectra were not clear owing to lac of ionization. Therefore, we decided to perform the UHPLC-MS analysis using the eluent containing formic acid (as reported in Materials and Methods), since we could calculate the % of isomerization for compounds **20**, **26** and **27** (the only obtained as E/Z mixtures) by analysing ^1^H-NMR spectra.

**References**

[1] A. Andreani, M. Granaiola, A. Leoni, A. Locatelli, R. Morigi, M. Rambaldi, L. Varoli, D. Lannigan, J. Smith, D. Scudiero, S. Kondapaka, R.H. Shoemaker, Imidazo[2,1-b]thiazole guanylhydrazones as RSK2 inhibitors, Eur J Med Chem 46 (2011) 4311–4323. https://doi.org/10.1016/j.ejmech.2011.07.001.

[2] P.T. Pham, D.K. Nguyen, N.T.S. Phan, M.V. Le, T.T. Nguyen, Oxidative annulation of acetophenones and 2-aminobenzothiazoles catalyzed by reusable nickel-doped LaMnO3 perovskites, RSC Adv 13 (2023) 3341–3345. https://doi.org/10.1039/d2ra08045a.

[3] S.G. Balwe, Y.T. Jeong, Iron-catalyzed unprecedented formation of benzo[d]imidazo[2,1-b]thiazoles under solvent-free conditions, RSC Adv 6 (2016) 107225–107232. https://doi.org/10.1039/c6ra24183b.

[4] M. Sevrin, C. Morel, M. Mangane, P. George, 2-thienylimidazo[2,1-b]benzothiazole-3-acetic acid derivatives, their preparation and their use, US5418248A, 1993.

[5] K. Ibe, Y.S. Hasegawa, M. Shibuno, T. Shishido, Y. Sakai, Y. Kosaki, K. Susa, S. Okamoto, Simple and practical method for selective acylation of primary hydroxy group catalyzed by N-methyl-2-phenylimidazole (Ph-NMI) or 2-phenylimidazo[2,1-b]benzothiazoles (Ph-IBT), Tetrahedron Lett 55 (2014) 7039–7042. https://doi.org/10.1016/j.tetlet.2014.10.130.

[6] T. Seki, T. Shigeyuki, H. Ryuichi, Imidazo[2,1-B]benzothiazole compounds and antiulcer compositions containing the same, US4968708A, 1989.

[7] A. Andreani, M. Rambaldi, D. Bonazzi, L. Greci, F. Andreani, Study on compounds with potential antitumor activity. III. Hydrazone derivatives of 5-substituted 2-chloro-3-formyl-6-methylindole, Farmaco Sci 34 (1979). https://doi.org/10.1002/chin.197924211.

[8] L. Sun, N. Tran, F. Tang, H. App, P. Hirth, G. McMahon, C. Tang, Synthesis and biological evaluations of 3-substituted indolin-2-ones: A novel class of tyrosine kinase inhibitors that exhibit selectivity toward particular receptor tyrosine kinases, J Med Chem 41 (1998) 2588–2603. https://doi.org/10.1021/jm980123I.

[9] C.F. Koelsch, A Synthesis of Ethyl Quininate from m-Cresol, J Am Chem Soc 66 (1944) 2019–2020. https://doi.org/10.1021/ja01240A007.

[10] A. Zakrzewska, E. Kolehmainen, B. Osmialowski, R. Gawinecki, 4-Fluoroanilines: synthesis and decomposition, J Fluor Chem 111 (2001) 1–10. https://doi.org/10.1016/S0022-1139(01)00401-8.

[11] R.J.S. Beer, H.F. Davenport, A. Robertson, 251. Some extensions of the synthesis of hydroxyindoles from p-benzoquinones, Journal of the Chemical Society (Resumed) (1953) 1262–1264. https://doi.org/10.1039/jr9530001262.

[12] L.K. Mehta, J. Parrick, F. Payne, Preparation of 3-Ethyloxindole-4,7-quinone, J Chem Res Synop (1998) 190–191. https://doi.org/10.1039/a707530h.

[13] P. Singh, A.K.S. Chauhan, R.J. Butcher, A. Duthie, Tellurium derivatives of 3-acetyl-2,5-dimethylthiophene: Synthetic and structural aspects, J Organomet Chem 728 (2013) 44–51. https://doi.org/10.1016/j.jorganchem.2012.12.038.

[14] B.K. Sarojini, B.G. Krishna, C.G. Darshanraj, B.R. Bharath, H. Manjunatha, Synthesis, characterization, in vitro and molecular docking studies of new 2,5-dichloro thienyl substituted thiazole derivatives for antimicrobial properties, Eur J Med Chem 45 (2010) 3490–3496. https://doi.org/10.1016/j.ejmech.2010.03.039.

[15] Y. Hu, C. Hu, G. Pan, C. Yu, M.F. Ansari, R.R. Yadav Bheemanaboina, Y. Cheng, C. Zhou, J. Zhang, Novel chalcone-conjugated, multi-flexible end-group coumarin thiazole hybrids as potential antibacterial repressors against methicillin-resistant Staphylococcus aureus, Eur J Med Chem 222 (2021) 113628. <https://doi.org/10.1016/j.ejmech.2021.113628>.

[16] E. Bellale, M. Naik, V. Vb, A. Ambady, A. Narayan, S. Ravishankar, V. Ramachandran, P. Kaur, R. Mclaughlin, J. Whiteaker, S. Morayya, S. Guptha, S. Sharma, A. Raichurkar, D. Awasthy, V. Achar, P. Vachaspati, B. Bandodkar, M. Panda, M. Chatterji, Diarylthiazole: An Antimycobacterial Scaffold Potentially Targeting PrrB-PrrA Two-Component System, (2014). https://doi.org/10.1021/jm500833f.
